# Cerebrospinal fluid biomarkers reveal transdiagnostic synaptic dysfunction across major psychiatric disorders

**DOI:** 10.1038/s41467-026-76187-y

**Published:** 2026-07-30

**Authors:** Andreas Göteson, Johanna Nilsson, Elena Camporesi, Anna Luisa Klahn, Elin Hörbeck, Robert Sigström, Lina Jonsson, Timea Sparding, Erik Pålsson, Aurimantas Pelanis, Anneli Goulding, Anniella Isgren, Sophie Erhardt, Simon Cervenka, Cynthia M. Bulik, Henrik Zetterberg, Kaj Blennow, Carl M. Sellgren, Ann Brinkmalm, Mikael Landén

**Affiliations:** 1 Department of Psychiatry and Neurochemistry, Institute of Neuroscience and Physiology, Sahlgrenska Academy, University of Gothenburg, Gothenburg, Sweden; 2 Department of Psychiatry for Affective Disorders, Sahlgrenska University Hospital, Gothenburg, Sweden; 3 Department of Psychotic Disorders, Sahlgrenska University Hospital, Gothenburg, Sweden; 4 Department of Medical Epidemiology and Biostatistics, Karolinska Institutet, Stockholm, Sweden; 5 Department of Physiology and Pharmacology, Karolinska Institutet, Stockholm, Sweden; 6 Department of Medical Sciences, Psychosis Research and Preventive Psychiatry, Uppsala University, Uppsala, Sweden; 7 Centre for Psychiatry Research, Department of Clinical Neuroscience, Karolinska Institutet & Stockholm Health Care Services, Stockholm, Sweden; 8 Department of Psychiatry, University of North Carolina at Chapel Hill, Chapel Hill, NC USA; 9 Department of Nutrition, University of North Carolina at Chapel Hill, Chapel Hill, NC USA; 10 Clinical Neurochemistry Laboratory, Sahlgrenska University Hospital, Mölndal, Sweden; 11 Department of Neurodegenerative Disease, Dementia Research Centre, UCL Institute of Neurology, Queen Square, London, UK; 12 UK Dementia Research Institute, University College London, London, UK; 13 Hong Kong Center for Neurodegenerative Diseases, Hong Kong, China; 14 Wisconsin Alzheimer’s Disease Research Center, University of Wisconsin-Madison, Madison, WI USA; 15 Department of Pathology and Laboratory Medicine, University of Wisconsin School of Medicine and Public Health, Madison, WI USA; 16 Centre for Brain Research, Indian Institute of Science, Bangalore, India

**Keywords:** Diagnostic markers, Prognostic markers

## Abstract

Synaptic dysfunction is increasingly recognized as a core feature of psychiatric disorders, yet fluid biomarkers that reflect such changes in vivo are lacking. Here, we applied targeted mass spectrometry to quantify low-abundant synaptic proteins in cerebrospinal fluid from 672 individuals with anorexia nervosa, attention-deficit/hyperactivity disorder (ADHD), bipolar disorder (BD), schizophrenia spectrum disorders (SCZ + ), and healthy controls. Synaptic protein levels were markedly reduced in SCZ + , with intermediate reductions in BD and ADHD. Using a data-driven approach to model transdiagnostic contrasts—psychotic experience, cognitive and functional impairment—a shared two-biomarker signature emerged: elevated LAMP1, a phagolysosomal marker, and reduced NPTX2, a synaptic activity marker which inhibits complement-dependent synapse elimination. Combining this ratio with polygenic scores improved diagnostic classification. Key findings were extended to an independent cohort at first-episode psychosis. These results support synaptic pathology as a measurable and transdiagnostic feature across several psychiatric disorders and highlight the potential of integrating fluid and genetic biomarkers.

## Abstract (web summary)

Changes in cerebrospinal fluid synaptic proteins were observed across several psychiatric disorders, including at schizophrenia illness onset. The LAMP1:NPTX2 ratio was linked to psychotic experience, cognitive deficits, and functional impairment.

## Introduction

Synaptic dysfunction is increasingly recognized as a core feature in the pathophysiology of major psychiatric disorders. This is most evident in schizophrenia (SCZ), where the developmental hypothesis proposes that excessive synaptic pruning during adolescence contributes to the onset of clinical symptoms^1,2^. Supporting this theory, (i) genetic risk variants for SCZ map to a diverse set of pre- and postsynaptic genes^3,4^, (ii) patient-derived cell models show increased synaptic pruning^5^, (iii) lower synaptic protein levels have been observed at illness onset via molecular imaging^6–8^ and cerebrospinal fluid (CSF)^9^, with more consistent findings in chronic stages^10–13^, (iv) synaptic proteins and transcripts at both bulk and single-cell resolution are downregulated in prefrontal cortex^14–16^, and (v) meta-analyses support lower levels of synaptophysin in the hippocampus, frontal, and cingulate cortex^17^, and lower density of postsynaptic elements in frontal cortex^18^. The mechanisms underlying this proposed synaptic loss remain unclear, but may involve increased complement-mediated microglial elimination^5^.

Beyond SCZ, evidence increasingly suggests that synaptic dysfunction is shared across multiple psychiatric disorders. A study of purified synaptic fractions from dorsolateral prefrontal cortex revealed similar proteomic changes in SCZ and bipolar disorder (BD)^15^. Similarly, transcriptomic profiling of postmortem human brain tissue has reported downregulated synaptic components in the shared pathology underlying a range of psychiatric conditions^14,19,20^. Moreover, genetic risk across several major psychiatric disorders appears to converge on synapse-related genes^4,21–23^.

Current methods to quantify synaptic changes in living humans remain limited. While molecular imaging offers regional specificity, CSF profiling offers high molecular resolution, enabling the detection of various synaptic proteins at low concentrations (picomolar). Proteins shed or released into lumbar CSF can thus reveal specific alterations across pre- and postsynaptic compartments. In previous work, we reported reduced CSF levels of synaptic proteins in BD across two independent cohorts^24^, and similar reductions have been described in smaller studies of other psychiatric conditions compared with controls^9,10,25–27^.

In this study, we quantified synaptic proteins, complement components, and established neurodegeneration biomarkers in CSF from a large and clinically diverse cohort (total *n* = 672) to identify candidate diagnostic biomarkers. Moreover, participants were characterized along key clinical dimensions—including psychotic experience, cognitive and functional impairments—to identify biomarker correlates of transdiagnostic impairments. Key findings were extended to an independent cohort of individuals with first–episode psychosis (FEP) and controls. Our results demonstrate that synaptic pathology is detectable in CSF across multiple psychiatric conditions, and that synaptic protein concentrations correlate with clinically relevant dimensions that transcend diagnostic boundaries.

## Results

### Demographics

The study included 505 cases diagnosed with major psychiatric disorders, comprising 73 with attention-deficit/hyperactivity disorder (ADHD), 40 with anorexia nervosa (AN), 319 within the BD spectrum, and 73 within the SCZ spectrum (SCZ + ), along with 167 healthy controls assessed at baseline (T1). CSF samples were collected during a clinically stable phase. The BD spectrum group was further stratified into bipolar type 1 (BD1, *n* = 140) and type 2 and related conditions (BD2 + , *n* = 179), which also included unspecified BD, cyclothymia, and recurrent depression. Demographics and clinical characteristics are presented in Table 1.

**Table 1:** Demographic and clinical characteristics of the study participants

	ADHD	AN	BD1	BD2 +	SCZ +	CON
*n*, T1	73	40	140	179	73	167
Age at CSF sampling, mean (sd)	35.1 (10.2)	27.3 (5.9)	39.6 (12.4)	38.7 (12.2)	35.7 (10.9)	39.5 (13.2)
Female sex, *n* (%)	34 (47%)	40 (100%)	74 (53%)	120 (67%)	24 (34%)	91 (54%)
Body mass index, mean (sd)	24.2 (4.5)	17.4 (1.7)	26.9 (5.0)	25.2 (4.7)	26.8 (4.1)	24.3 (3.7)
Diagnostic subtype, *n* (%)	ADD (*n* = 18, 25%); ADHD (*n* = 52, 71%), ADHD unspecified (*n* = 3, 4%)	Anorexia nervosa (*n* = 40, 100%)	BD1 (*n* = 140, 100%)	BD2 (*n* = 157, 87%); BD not otherwise specified (*n* = 10, 6%); cyclothymia (*n* = 7, 4%); recurrent depression (*n* = 5, 3%)	Schizophrenia (*n* = 36, 49%), schizoaffective (*n* = 11, 15%), substance-realated psychotic disroders (*n* = 2, 3%), other primary psychotic disorders (*n* = 22, 30%), other related disorders (*n* = 2, 3%)	N/A
Years of illness, median (range)	21.0 (1.0-50.0)	10.5 (0.0–31.0)	16.0 (0.0-50.0)	16.0 (0.0-52.0)	7.0 (0.0-36.0)	N/A
Number of hospitalizations, mean (sd)	0.0 (0.0, 1.0)	N/A^a^	3.0 (1.0, 5.0)	1.0 (0.0, 2.0)	2.0 (1.0, 4.0)	N/A
Lifetime psychotic experience, *n* (%)	1 (1%)	N/A^a^	71 (51%)	19 (11%)	71 (97%)	
Comorbid substance abuse, *n* (%)	4 (5.6%)	N/A^a^	13 (9.3%)	16 (8.9%)	12 (17%)	N/A
Current medication at CSF sampling, *n* (%)		N/A^a^				N/A
Antidepressants	22 (31%)		44 (31%)	105 (59%)	21 (30%)	
Antiepileptic mood stabilizers	1 (1.4%)		46 (33%)	87 (49%)	2 (2.9%)	
Central stimulants	57 (79%)		8 (5.7%)	16 (8.9%)	0 (0%)	
First-generation antipsychotics	0 (0%)		10 (7.1%)	3 (1.7%)	3 (4.3%)	
Lithium	0 (0%)		102 (73%)	79 (44%)	10 (14%)	
Second-generation antipsychotics	4 (5.6%)		55 (39%)	32 (18%)	54 (77%)	

*AN* anorexia nervosa, *ADHD* attention-deficit/hyperactivity disorder, *BD1* bipolar disorder type 1, *BD2+* bipolar disorder type 2 and related disorders, *BMI* body mass index, *CSF* cerebrospinal fluid, *SCZ+* schizophrenia spectrum disorders.

^a^Data was not systematically collected

### CSF synaptic protein panels

We quantified 37 synaptic proteins in CSF using liquid chromatography-tandem mass spectrometry (LC-MS/MS), following previously established protocols^28–30^. The proteins covered a broad range of synaptic processes, including the presynaptic vesicle cycling and release machinery (e.g., SNAP25, VAMP2, SYT1), the neurexin-neuroligin complex involved in transsynaptic adhesion (e.g., NRX1A, NLGN2, CSTN3), neuronal cell adhesion at synapses (e.g., NCAM2, THY1), postsynaptic receptor signaling and modulation (e.g., GRIA4, NEUG, NPTX2), neuropeptide signaling (VGF, SCG2), miscellaneous synaptic proteins (e.g., APP, SYUB, 1433Z), and synapse-expressed endolysosomal proteins (CTSD, LAMP1).

### Non-disease covariance of synaptic proteins in healthy controls

**Fig. 1:**
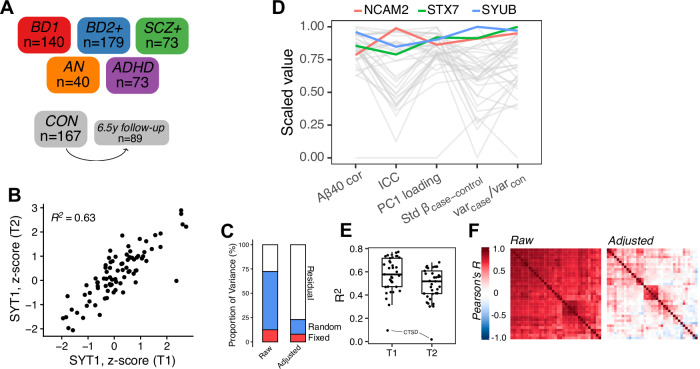
Overview of the study design and longitudinal analyses in controls. **A** Graphical representation of the study cohort. **B** The correlation of synaptotagmin-1 (SYT1) CSF concentrations at baseline (T1) and the 6.5-year follow-up (T2) in controls. SYT1 was chosen as a demonstrative example. **C** Proportion of variance explained by fixed effects (protein assay, age, sex, timepoint), random effects (individual), and residual variance, based on raw CSF concentrations and adjusted for the non-disease covariance reference (cov_nd_). **D** Five score ranking of synaptic proteins for the cov_nd_ reference, scaled by min–max scaling. The top-ranked proteins (NCAM2, STX7, and SYUB) by the averaged five scores were combined to a composite reference (i.e., cov_nd_) and were represented in color. **E** Boxplot of the variance explained (R^2^) by cov_nd_ in synaptic protein concentrations at T1 and T2, accounted for age and sex. Box plots show the median with interquartile range, and whiskers extend to the smallest and largest values within 1.5 × interquartile range across the *n* = 37 synapse-expressed proteins; points show individual values. CTSD is labeled because this endolysosomal protein is not uniquely expressed in synapses. **F** Heatmaps of protein-protein correlations in healthy controls at T1, based on raw CSF concentrations (left) and values adjusted for non-disease covariance (right). Source data are provided as a Source Data file.

The aim of this study was to identify fluid biomarkers for major psychiatric disorders. However, CSF protein levels vary substantially across healthy individuals^31^, introducing noise that may obscure disease-related effects. To characterize such non-disease variability in synaptic protein levels, we analyzed CSF from a subset of the healthy controls (*n* = 89; median age=36) with repeated sampling after a median of 6.5 years (range 5–9; Fig. 1A). Synaptic protein concentrations were remarkably stable within individuals, with baseline concentrations explaining on average 60% of the variance at follow-up (mean [SD] R² = 0.60 [0.12]; e.g., SYT1, R^2^ = 0.63; Fig. 1B). Moreover, synaptic proteins were strongly intercorrelated at both timepoints (average Pearson’s *r* = 0.73; Fig. S1). Consistent with these observations, a linear mixed-effects model used to partition sources of variance in this repeated-measures dataset from controls showed that the individual term accounted for 60% of the total variance, whereas assay differences, age, sex, and timepoint combined explained only 12% (Fig. 1C). Together, these findings indicate that synaptic protein concentrations in CSF are largely defined by stable, individually determined covariance in the absence of disease and advanced age.

Previous studies have shown that adjusting for non-disease covariance using pairwise biomarker ratios or reference proteins can improve signal-to-noise and reduce false positives in CSF biomarker studies^32,33^. To adapt this strategy, we defined criteria for an ideal reference protein and ranked the measured synaptic proteins accordingly (Fig. 1D). Based on these criteria, the three top-ranked proteins (SYUB, STX7, and NCAM2) were combined using singular value decomposition to define a composite reference approximating non-disease covariance in this dataset, hereafter termed cov_nd_.

The cov_nd_ reference (calculated at T1) explained a substantial proportion of variance in synaptic protein concentrations at both T1 and T2 (mean [sd] R² at T1 = 0.58 [0.15] and T2 = 0.49 [0.14]; Fig. 1E). Adjusting protein levels for cov_nd_ attenuated protein-protein correlations (average *R* = 0.21, Fig. 1F) but preserved biologically relevant relationships (Fig. S1). Reducing the overall CSF covariance should also improve the genetic prediction of specific protein levels. To test this, we extracted expression quantitative trait loci (eQTLs) derived from human cortical brain tissue (post-mortem)^34^ and evaluated their effects in CSF protein levels in controls (*n* = 132 with genetic data). While several reported eQTLs had no detectable effect in the raw CSF data, adjusting for cov_nd_ revealed more of the expected genetic associations with stronger effect sizes (Table S5).

### Case-control comparisons of CSF synaptic proteins across disorders

Synaptic protein concentrations were compared between each diagnostic group and baseline controls, both with and without adjustment for cov_nd_. Using raw CSF levels, we identified 30 significantly altered synaptic proteins in SCZ + , 18 in BD1, 8 in ADHD, and none in BD2+ or AN (1% false discovery rate; Table S3; Fig. S2). The strongest effect sizes were observed in SCZ + , characterized by generally lower concentrations across the synapse biomarker panel, most notably for NPTX2 (β [95% CI] = –1.0 [–1.3, –0.8]) and GRIA4 (–0.9 [–1.2, –0.6]).

**Fig. 2:**
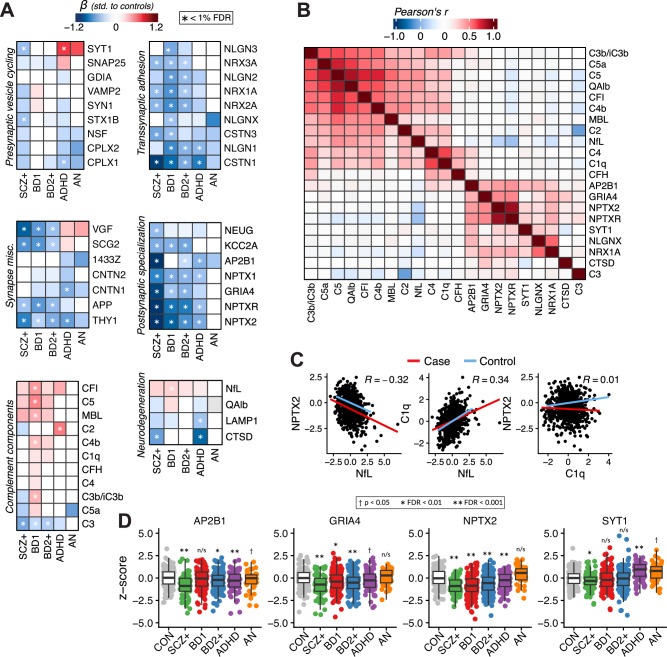
Results from main case-control analyses. Group sizes: controls, *n* = 167; schizophrenia spectrum (SCZ + ), *n* = 73; bipolar disorder type 1 (BD1), *n* = 140; bipolar disorder type 2 and related conditions (BD2 + ), *n* = 179; attention-deficit/hyperactivity disorder (ADHD), *n* = 73; anorexia nervosa (AN), *n* = 40. **A** Heatmaps of effect sizes (β-values, standardized to controls) for each disorder compared with controls, showing associations at <20% false discovery rate; significant associations at 1% false discovery rate (FDR) are marked by an asterisk. The color scale reflects association strength. **B** Protein–protein correlation matrix of complement components, NfL, CSF/serum albumin quotient (QAlb), and selected synaptic proteins. Color scale reflects correlation strength (Pearson’s r). **C** Pairwise correlation plots showing: NfL—a marker of neuroaxonal degeneration; C1q—activator of the classical complement pathway; NPTX2—a secreted synaptic protein and regulator of C1q activity in the brain. Regression lines are shown separately for cases (red) and controls (blue). **D** Boxplots representing four selected synaptic proteins. Box plots show the median with interquartile range, and whiskers extend to the smallest and largest values within 1.5 × interquartile range; points show individual samples. For all panels, synaptic proteins were regressed on cov_nd_. Two-sided statistics are from linear regression models; † nominal *P* < 0.05, * FDR < 0.01, ** FDR < 0.001. Abbreviations: AP2B1 (AP-2 complex subunit beta), GRIA4 (glutamate receptor subunit A4), NPTX2 (neuronal pentraxin 2), and SYT1 (synaptotagmin-1). Source data are provided as a Source Data file.

The strongest associations were more clearly differentiated in analyses adjusted for cov_nd_ (Fig. 2A). In SCZ + , large reductions were observed for proteins involved in glutamate signaling, including the α-Amino-3-hydroxy-5-methyl-4-isoxazolepropionic acid (AMPA) receptor subunit GRIA4, neuronal pentraxins (NPTX2, NPTXR), and AP2B1. In ADHD, lower levels of endolysosomal proteins (CTSD, LAMP1) and higher levels of SYT1 were observed (Fig. 2D). BD1 and BD2+ shared lower levels of neurexin–neuroligin family proteins, involved in transsynaptic adhesion. However, most associations were present in more than one disorder (e.g., CSTN1, NPTX2, NPTXR), with minor variations in effect size rather than categorical diagnostic specificity. Certain associations identified using raw CSF abundances (e.g., CNTN2, CPLX2) were attenuated after cov_nd_ adjustment, suggesting limited specificity beyond the shared covariance structure.

To assess the case-control discrimination of individual proteins, we calculated area under the receiver operating characteristic curve (AUC) statistics adjusting for age, sex, and cov_nd_. The top-performing biomarkers for each diagnostic group was NPTX2 for SCZ+ (AUC [95% CI] = 0.78 [0.72–0.85]), NPTX2 for BD1 (0.73 [0.67–0.78]), NPTXR for BD2+ (0.71 [0.66–0.77]), SYT1 for ADHD (0.73 [0.66–0.81]), and NLGNX for AN (0.69 [0.60–0.79]).

### Complement components and neurodegeneration biomarkers as correlates of synaptic dysfunction

Complement-mediated synapse elimination may underlie synaptic loss. We therefore analyzed CSF concentrations of complement components, alongside biomarkers of neuroaxonal degeneration (neurofilament light chain, NfL), and blood-CSF barrier integrity (CSF/serum albumin quotient), to contrast with synaptic proteins. Results are shown in Fig. 2 and Table S3.

Compared with controls, CSF NfL concentrations were significantly higher in BD1 (0.25 [0.10, 0.40], *P* = 0.001), with similar effect size estimates in SCZ+ (β [95% CI] = 0.24 [0.01, 0.48], *P* = 0.042), BD2+ (0.19 [0.06, 0.32], *P* = 0.004), and AN (0.25 [0.02, 0.48], *P* = 0.03), but only BD1 remained statistically significant after multiple-testing correction. Cases with ADHD did not differ from controls (0.15 [−0.02, 0.31], *P* = 0.078). Regarding the CSF/serum albumin quotient, no significant case-control associations were observed, as previously reported from a subset of these cohorts^35^.

With respect to complement components, we found significantly lower levels of complement C3 in individuals with SCZ + , BD1, and BD2+ compared with controls, and higher levels of complement C2 in ADHD compared with controls (β [95% CI] = 0.52 [0.25, 0.78], *P* = 1.6e-4). While total C4 was not associated with any diagnostic group, activated C4 (C4b) was significantly higher in BD1 compared with controls (β [95% CI] = 0.32 [0.10, 0.53], *P* = 0.004), with concordant effect direction in SCZ+ and BD2 + . C4 isoproteins (C4A and C4B) could not be distinguished in the available assays.

Synaptic proteins and complement components were not correlated in CSF (Fig. 2B). However, NfL concentrations correlated negatively with NPTX2 and NPTXR regressed on cov_nd_ (*R* = −0.32 and −0.40, respectively; Fig. 2C shows NPTX2). This correlation was consistent across both cases and controls and may reflect a low-grade neuroaxonal and synaptic loss that is more pronounced in case groups.

### Data-driven models identify a CSF biomarker ratio reflecting psychotic liability, cognitive and functional impairment

Psychiatric disorders are notably comorbid and diagnostic boundaries lack clear biological validity. Therefore, we aimed to identify biomarker associations of transdiagnostic dimensions—specifically psychotic experience, cognitive and functional impairments—using a data-driven approach. First, we trained L1-penalized logistic regression models (LASSO) within a repeated nested cross-validation for feature selection. Second, we employed recursive feature elimination to simplify each model for clinical utility, retaining the two most informative biomarkers. These models used raw CSF concentrations as multivariate models can inherently adjust for non-disease covariance. Participants with AN were excluded due to missing clinical data, and most individuals with SCZ+ had not undergone cognitive testing.

**Fig. 3:**
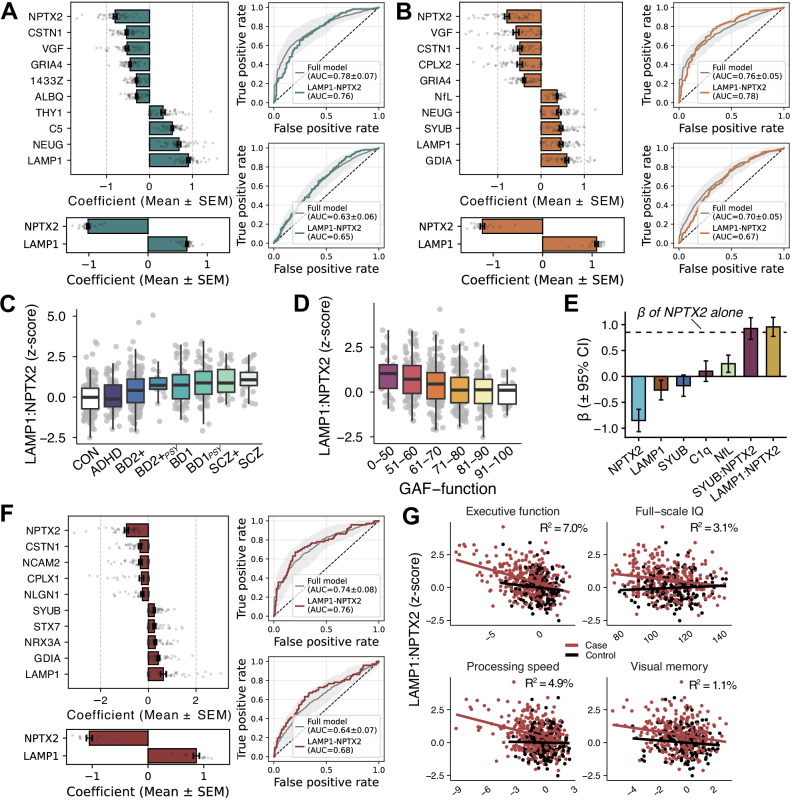
Data-driven biomarker models for psychotic experience (total *n* = 634), cognitive impairment (total *n* = 477), and functional impairment (total *n* = 588). **A**, **B**, **F** Coefficients from the L1-regularized models (top) and the recursive feature elimination (RFE) models (bottom), along with ROC curves illustrating model performance. Each panel compares cases with psychotic experience (**A**), functional impairment (**B**), or cognitive impairment (**F**) to controls (top) and to cases without the respective condition (bottom). Summary statistics are based on 50 held-out validation sets. **C** LAMP1:NPTX2 ratio across diagnostic groups, stratified by history of psychotic experience. Cases with BD1 or BD2+ and psychotic experience are marked with ‘PSY’. **D** LAMP1:NPTX2 ratio in relation to clinician-rated global functioning (GAF-F, *n* = 560 with available data). **E** Case-control association statistics for the combined group SCZ+ and BD1 (*n* = 213) compared with controls (*n* = 167). Showing β coefficients standardized to controls with 95% confidence intervals for NPTX2, LAMP1, and the ratio LAMP1:NPTX2. This is contrasted with SYUB—the highest ranked protein for non-disease-related covariance—and its ratio to NPTX2, NfL—a marker of neuroaxonal injury, and C1q—activator of the classical component pathway. **G** Correlations between LAMP1:NPTX2 ratio and composite scores for cognitive domains. Box plots show the median with interquartile range, and whiskers extend to the smallest and largest values within 1.5 × interquartile range; points show individual samples. Source data are provided as a Source Data file.

Psychotic experience was defined as the lifetime experience of psychotic symptoms based on study interview and national register data. Cases with psychotic experience were distinguished from controls (mean [SD] AUC_case-con_ = 0.78 [0.07]) and cases without psychotic experience (AUC_case-case_ = 0.63 [0.06]), with models influenced by NPTX2, LAMP1, CSTN1, and NEUG (Table S6). The simplified two-biomarker models consistently featured LAMP1 and NPTX2, which contributed with similar but opposite weights (Table S7). Hence, we calculated the LAMP1:NPTX2 ratio as a proxy biomarker, which retained the performance of the full models (Fig. 3A). In the full cohort, this ratio showed a progressive increase along the ADHD-BD-SCZ spectrum, with and without psychotic experience (*P* for trend<0.05; Fig. 3C), suggesting relevance to psychotic liability that extends beyond diagnostic categories.

Cognitive performance was assessed in 310 cases and 167 controls, with cognitive impairment defined by scores <1.96 SD below the control mean (*n* = 73). Cognitive impairment was predicted mainly by NPTX2, LAMP1, GDIA, and CSTN1 (mean [SD] AUC_case-con_ = 0.74 [0.08], AUC_case-case_ = 0.64 [0.07]; Table S6). Again, LAMP1 and NPTX2 frequently selected in the simplified models, and the LAMP1:NPTX2 ratio retained the performance of the full model (Fig. 3F; Table S7). The LAMP1:NPTX2 ratio correlated with composite scores of executive function (R^2^ = 0.070, *P* = 4.2e-9), processing speed (R^2^ = 0.049, *P* = 7.9e-7), visual memory (R^2^ = 0.011, *P* = 0.022), and general cognitive ability (R^2^ = 0.031, *P* = 9.1e-5; Fig. 3G). These associations were not significant in controls alone, indicating that the LAMP1:NPTX2 ratio captures cognitive deficits beyond normal variability.

Functional impairment was defined as recurrent long-term sick leave ( > 60 days/year) during the year of CSF sampling and the four subsequent years, based on longitudinal data from national insurance registers^36^. The functionally impaired subgroup (*n* = 155) was mainly predicted by NPTX2, GDIA, VGF, and LAMP1 (mean [SD] AUC_case-con_ = 0.76 [0.05], AUC_case-case_ = 0.70 [0.05]; Table S6). The simplified models again consistently featured LAMP1 and NPTX2 with the ratio retaining the accuracy of the full model (Fig. 3B; Table S7). The LAMP1:NPTX2 ratio showed a negative correlation with clinician-rated global functioning (GAF-function; *P* for trend<0.05; Fig. 3D). For the combined group of BD1 and SCZ + , the LAMP1:NPTX2 ratio improved case-control association over NPTX2 alone (β [95% CI] = 0.96 [0.77, 1.14] vs. −0.85 [−0.63, −1.07]; Fig. 3E).

Together, these results suggest a shared underlying biology across psychotic liability, cognitive deficits and functional impairments, implicating synaptic dysfunction. Moreover, our results indicate that the CSF LAMP1:NPTX2 ratio reflects these disabilities in a clinical population.

### Estimating the influence of psychotropic medications on candidate biomarkers

To evaluate the effect of medication on case–control-associated proteins, we used linear regression models to estimate the proportion of variance in protein concentrations attributable to medication. Among the top-performing biomarkers, only GRIA4 showed a modest association with antipsychotics (R^2^ = 0.018), suggesting that medication is unlikely to be a major driver of the observed case–control differences. The most prominent medication-related effects involved lithium and presynaptic vesicle cycling proteins (e.g., CPLX1, CPLX2; Table S4), which were not central to the primary case–control results.

### Combining fluid biomarkers with polygenic scores to augment predictive performance

**Fig. 4:**
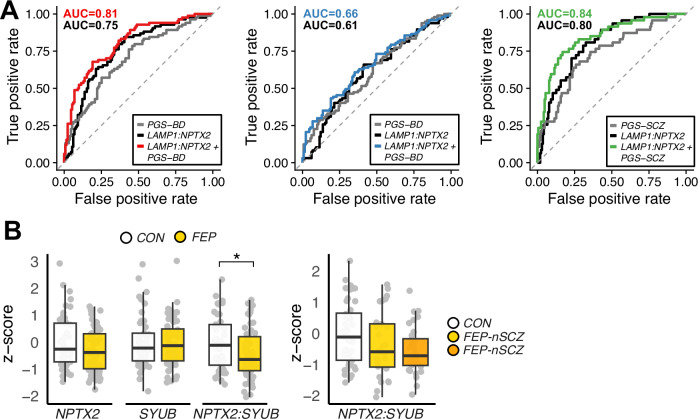
Extending NPTX2 as a biomarker for bipolar disorder (BD) and schizophrenia (SCZ). **A** Diagnostic performance of the CSF ratio LAMP1:NPTX2, polygenic scores (PGS) for BD or SCZ, and their combination. Colors indicate different comparisons: red—BD1 vs. control; blue—BD2+ vs. control; green: SCZ vs. control. **B** Boxplots of NPTX2, SYUB, and the NPTX2:SYUB ratio in individuals with first-episode psychosis (FEP, *n* = 60), including 35 individuals later diagnosed with schizophrenia (FEP-SCZ), and controls (*n* = 45). Box plots show the median with interquartile range, and whiskers extend to the smallest and largest values within 1.5 × interquartile range; points show individual samples. Two-sided statistics are from linear regression models; * nominal *P* < 0.05. Source data are provided as a Source Data file.

Psychiatric disorders are highly heritable, and we hypothesized that polygenic scores (PGSs) may improve the discriminative capacity of CSF biomarkers. Genetic data were available for 228 cases (47 SCZ, 84 BD1, 97 BD2 + ) and 166 controls. The LAMP1:NPTX2 ratio was evaluated as a candidate biomarker and was not correlated with PGS-SCZ or PGS-BD after adjustment for diagnosis (*P* > 0.05). However, combining LAMP1:NPTX2 with PGS-BD or PGS-SCZ modestly improved case–control discrimination, although AUC improvements were not statistically significant (*P* = 0.07–0.11): AUCs increased from 0.80 to 0.84 for SCZ, from 0.75 to 0.81 for BD1, and from 0.61 to 0.66 for BD2+ (Fig. 4A). These findings support further development of combined genetic and fluid biomarkers for diagnostic stratification in psychiatric disorders.

### Synaptic dysfunction is detectable at first-episode psychosis

Finally, to evaluate the relevance of our findings at psychotic illness onset, we validated synaptic biomarkers in an independent cohort of individuals with first-episode psychosis (FEP; *n* = 60 (25 female); mean age = 28) and controls (*n* = 45 (23 female); mean age=26). Cases were followed clinically for 1.5 years to determine diagnostic outcomes. A subset of the synaptic biomarkers had previously been analyzed in this cohort using the same LC-MS/MS protocol^37^. Although LAMP1 was not available in this dataset, we were able to evaluate NPTX2. To account for non-disease covariance, NPTX2 was expressed as a ratio to SYUB, the top-ranked protein for non-disease covariance (Fig. 1A).

Comparing FEP individuals with controls, we found no significant difference in raw CSF concentrations of NPTX2 (β [95% CI] = −0.30 [−0.65, 0.05], *P* = 0.10). However, NPTX2:SYUB levels were significantly lower in FEP compared to controls (β [95% CI] = −0.41 [−0.77, −0.04], *P* = 0.03). This effect seemed primarily driven by individuals later diagnosed with schizophrenia (FEP-SCZ; β [95% CI] vs controls = −0.56 [−0.97, −0.14], *P* < 0.01), rather than those with non-schizophrenia outcomes (FEP-nSCZ; β [95% CI] vs controls = −0.25 [−0.73, 0.24], *P* = 0.31; Fig. 4B), although FEP-SCZ and FEP-nSCZ did not differ statistically (FEP-SCZ vs FEP-nSCZ: β [95% CI] = −0.27 [−0.79, 0.25]; p = 0.30). Notably, SYUB levels did not differ between FEP and controls (β [95% CI] = −0.09 [−0.46, 0.28], *P* = 0.63).

## Discussion

Using targeted LC-MS/MS, we quantified low-abundant synaptic proteins in CSF revealing that synaptic pathology is detectable in vivo across a large and clinically heterogeneous cohort of individuals with major psychiatric disorders. The most pronounced reductions relative to controls were observed in individuals with SCZ + , followed by those with BD1, BD2 + , and ADHD. Very few biomarkers were disorder-specific. Moving beyond diagnostic categories, we modelled key transdiagnostic clinical dimensions—psychotic experience, cognitive and functional impairments—and identified a shared synaptic signature, with the LAMP1:NPTX2 ratio emerging as a robust proxy biomarker. These findings indicate that synaptic dysfunction represents a measurable and clinically relevant pathology in several psychiatric disorders.

The biomarker associations cut across diagnostic categories and appear to reflect underlying symptom dimensions rather than diagnostic specificity. Some proteins nevertheless showed stronger signals in certain groups; for example, GRIA4 was most clearly reduced in SCZ + , whereas SYT1 was mainly elevated in ADHD. Whether such patterns reflect clinically meaningful subdimensions remains to be determined. Notably, while reductions in synaptic proteins were pronounced in several groups, NfL showed only modest and less consistent increases. This contrasts with most neurodegenerative disorders, where NfL changes are typically more prominent in chronic stages^38,39^.

The LAMP1:NPTX2 ratio correlated with chronic and severe clinical states, including psychotic experience, cognitive deficits and functional impairment. NPTX2 is an immediate-early gene secreted upon synaptic activity for postsynaptic specialization at excitatory synapses. Specifically, NPTX2 interacts with NPTXR and GRIA4 on parvalbumin-expressing interneurons to regulate excitation-inhibition balance^40–42^, which is critical for cognitive processes and potentially disrupted in certain neuropsychiatric disorders^43^. Further, NPTX2 inhibits complement C1q activity in the brain^44^, and whereas CSF C1q and total C4 were unaltered, activated C4 (C4b) was increased in BD1 with concordant effect direction in SCZ + , BD2 + , and ADHD, consistent with findings in Nptx2^KO^ mouse brains^44^. NPTX2 CSF levels are reduced in various cognitive disorders (including SCZ) and are likely not disease-specific^9,38,45–47^. While several biomarker ratios featuring NPTX2 have been proposed^45,46^, the ratio to LAMP1 is notable as LAMP1 is a ubiquitous lysosomal membrane protein upregulated by activated phagocytotic cells such as microglia^48^. Thus, our data combined with previous experimental data indicate that elevated LAMP1:NPTX2 could represent a CSF biomarker for phagocytic synaptic elimination. This requires experimental validation. Notably, while LAMP1 was not associated with SCZ+ or BD in our data (only a moderate decrease in ADHD), the LAMP1:NPTX2 ratio improved case-control stratification relative to NPTX2 alone. A similar improvement with SYUB:NPTX2 suggests that LAMP1 mainly contributes through shared covariance rather than mechanism-specific effects.

Longitudinal analyses indicate that individually determined covariance is a stable and dominant source of variability in CSF synaptic proteins in the absence of disease and advanced age. Accounting for such non-disease covariance may enhance signal-to-noise in CSF biomarker studies^32,33^. Notably, in our cohort sampled at illness onset—a critical window for biomarker-based diagnostics—NPTX2 reductions were only detectable after such adjustment, with unadjusted models narrowly missing statistical significance. However, the specific reference constructed in this study (cov_nd_) was not intended for general use. Further work will be needed to define broadly applicable reference strategies.

This study represents an early stage of biomarker discovery, with limitations to consider. The data arise from naturalistic clinical cohorts, where factors such as medication exposure and other clinical characteristics cannot be fully disentangled from disease-related biology. Independent validation in external cohorts—and potentially translation to less invasive modalities—will therefore be necessary before clinical application can be considered. Diagnostic group sizes were uneven, with a majority of cases in the BD spectrum. Further, cases with AN and SCZ+ were missing certain data modalities. However, the broad symptom profile of BD may render it a useful proxy for psychiatric illness more generally. The AN group was small (*n* = 40), younger than controls, and compared only to female controls. Strict multiple-testing correction may have reduced power in this subgroup, as several associations were notable (e.g., higher SYT1, lower NLGNX) but did not reach the strict 1% false discovery rate threshold. All case-control results are reported in the Supplementary Tables.

In conclusion, this study shows that synaptic dysfunction is detectable in CSF across several major psychiatric disorders and relates to clinically relevant outcomes. The LAMP1:NPTX2 ratio emerged as a robust proxy biomarker for such pathology, potentially reflecting excessive synaptic elimination. Key results were extended to FEP and corroborate prior evidence that synaptic dysfunction is detectable already at illness onset in SCZ. Our results further suggest that fluid and genetic biomarkers combined may have potential to advance biologically informed patient stratification in psychiatry, but replication is warranted.

## Methods

The included study cohorts were all approved by the Stockholm Regional Ethics Committee and conducted in accordance with the Declaration of Helsinki. All participants provided informed consent.

### Study cohorts

#### The St Göran project

Participants were primarily recruited from the St. Göran Project, a longitudinal naturalistic cohort study. The design and cohort characteristics have been described in detail elsewhere^49^. In brief, cases were recruited from psychiatric outpatient clinics and underwent comprehensive clinical profiling, including structured diagnostic interviews, neuropsychological testing, and blood and CSF sampling. While the initial inclusion criterion was a diagnosis within the bipolar spectrum, the project was later expanded to include individuals with attention-deficit/hyperactivity disorder (ADHD) and primary psychotic disorders (SCZ + ), each forming separate but structurally similar study arms. Minor protocol adaptations were made to align with clinical routines at each outpatient site. For example, the SCZ+ group (also referred to as the GRIP cohort^37^) included individuals with recent-onset psychotic symptoms or those requiring further diagnostic evaluation, and was not systematically assessed with the same neurocognitive battery.

Controls were recruited from the general Swedish population via random sampling by Statistics Sweden (www.scb.se). Eligibility required no personal or first-degree family history of major psychiatric conditions^49^. Controls received a small financial reimbursement.

#### The Anorexia Nervosa Genetics Initiative

The Anorexia Nervosa Genetics Initiative is an international collaboration focused on identifying genetic factors contributing to AN^50^. In Sweden, a subset of enrolled participants also underwent lumbar puncture for CSF collection (*n* = 40). These cases were recruited through the Stockholm Centre for Eating Disorders, and medical history data were collected via a study questionnaire.

#### The Karolinska Schizophrenia Project

The Karolinska Schizophrenia Project investigates the development and early course of schizophrenia. Participants were young adults (ages 18–40) presenting for clinical care during their first psychotic episode. Exclusion criteria included extensive prior antipsychotic treatment, serious somatic or neurological illness, substance use disorders (except nicotine), and autism. Participants underwent clinical and diagnostic assessments and drug screening. Baseline diagnoses were made according to DSM-IV criteria, and diagnostic outcomes were determined after 1.5 years of follow-up, resulting in classification as either schizophrenia (FEP-SCZ) or other psychotic disorders (FEP-nSCZ)

### Cerebrospinal fluid sampling

Lumbar punctures were performed during stable mood states, between 9:00 and 11:00 a.m., with participants adhering to their prescribed medication regimen. A fine, disposable spinal needle was used to extract 12 mL of CSF from the L3/L4 or L4/L5 interspace. The sample was gently inverted to avoid concentration gradients, aliquoted, and stored at –80 °C until analysis.

### Experimental procedures

The CSF biomarkers were analyzed using distinct protocols, as outlined below.

#### LC-MS/MS analysis of synapse biomarkers

Two LC-MS/MS panels were utilized for biomarker quantification. Panel 1 included a mixed panel of 28 synaptic proteins (Table S1). Panel 2 included seven proteins from the two transsynaptic binding protein families of neurexins (NRXN) and neuroligins (NLGNs). For both biomarker panels, the same sample preparation protocol was followed, previously described in detail^29,30^. In summary, 25 µL of a mix of heavy standard peptides serving as an internal standard (JPT Peptide Technologies (Berlin, Germany; SpikeTides L), for concentration see Table S1) was added to 100 µL of CSF sample. Samples were then reduced, alkylated, digested, and desalted using Oasis 30 µm HLB 96-well µElution plates (Waters Co., Milford, MA, USA).

The quantification of Panel 1 was performed using liquid chromatography tandem mass-spectrometry (LC-MS/MS) with a micro-high-performance LC-MS/MS system (6495 Triple Quadrupole LC/MS system, Agilent Technologies) equipped with a Hypersil Gold reversed-phase column (100 × 2.1 mm, 1.9 µm particle size, Thermo Fisher Scientific). Panel 2 was quantified on a Q Exactive hybrid quadrupole–orbitrap mass spectrometer (Thermo Fisher Scientific) coupled to a Vanquish UHPLC (Thermo Fisher Scientific). Peptides were separated on a 2.1 mm Hypersil Gold reversed-phase column. Further details on instrument settings are provided in Table S2. Skyline 20.1 (MacCoss Lab Software) was utilized to analyze the mass spectrometric data.

The FEP cohort was analyzed using a very similar technique but with minor modifications, as previously described^37^.

#### LC-MS/MS analysis of SNAP25 and SYT1

For SNAP-25 and SYT1 measurements, an in-house assay was used where the sample preparation consisted of enrichment with immunoprecipitation (KingFisher™ Flex System) of 200 µL CSF, followed by digestion, and the addition of heavy isotope-labeled standards (Thermo Fisher Scientific, conc 100 pmol/L). Quantification was then performed using a micro-high-performance LC-MS/MS system (6495 Triple Quadrupole LC/MS system, Agilent Technologies) equipped with a Hypersil Gold reversed-phase column (100 × 2.1 mm, 1.9 µm particle size, Thermo Fisher Scientific). For details on sample preparation and MS settings, refer to previously published work^28^ and Table S2.

#### Immunoassay analyses of complement components, neurofilament light chain and albumin

Complement proteins were measured using a multiplexed Luminex MAGPIX panel according to the manufacturer’s instructions (Luminex Corp., Austin TX). CSF NfL concentration was measured using an in-house enzyme-linked immunosorbent assay, as previously described^51^. Albumin concentrations were analyzed by immunonephelometry on a Beckman Immage Immunochemistry system (Beckman Instruments, Beckman Coulter, Brea, CA, USA).

### Statistics and reproducibility

Protein concentrations were log₁₀-transformed to meet normality assumptions and standardized to z-scores based on control participants at T1 (mean=0, SD = 1). No statistical method was used to predetermine sample size. No available data were excluded from the analyses.

#### Adjusting for non-disease covariance

Candidate reference proteins were evaluated through a multi-step ranking approach, based on a composite of five criteria. First, we ranked proteins by standardized beta absolute values from the primary case-control comparisons, lower values preferred. Second, we compared the variance of each biomarker in cases and controls, favoring proteins with similar variance across groups. Third, we assessed loadings on the first principal component in female and male controls at T1 and T2 to identify proteins with strong and robust correlation to the main covariance structure of synapse biomarkers. Fourth, we used linear mixed models in longitudinal control data to estimate the intraclass correlation coefficient, identifying proteins with high within-individual stability over time. Fifth, we calculated Pearson’s correlation coefficients between synaptic proteins and amyloid-beta 40, previously suggested as a reference marker^32^ and measured in a subset of the participants (*n* = 121 cases and 71 controls)^52^. Each score was min-max scaled, and final composite rank was computed by averaging across all five criteria. The top three proteins (SYUB, STX7, and NCAM2) were used to construct a non-disease covariance reference (cov_nd_) through singular value decomposition using the NIPALS algorithm. When referenced in the main text, synaptic protein concentrations were regressed on cov_nd_ and re-standardized to z-scores. Extended details are provided in the Supplementary Material.

#### Statistical analyses

Protein–protein correlations were calculated using Pearson’s correlation. Longitudinal correlations were estimated using linear regression models. The variance in T2 protein levels explained by T1 protein levels was estimated by first fitting a model including age and sex only, and then comparing it to a full model that also included baseline protein abundance. The difference in R² between these models was interpreted as the variance attributable to the baseline biomarker.

Case–control comparisons were performed using linear regression for each diagnostic group versus controls at T1, adjusting for age and sex. ROC curves were derived from predicted probabilities, and AUCs were compared using DeLong’s test. For comparisons involving AN, only female controls were included. Statistical significance was defined using an adjusted *P*-value threshold corresponding to a 1% false discovery rate, accounting for all five diagnostic groups. Variance explained by medication was estimated in linear models adjusting for age, sex, diagnostic group, and cov_nd_ (for synaptic proteins). Tests for trend across ordered groups were performed using linear regression models. Variance explained in cognitive scores was estimated using linear regression models after residualizing both biomarker and cognitive variables for age and sex based on the control group.

#### Definition of transdiagnostic contrasts

Psychotic experience was defined using combined interview and national register data, requiring either concordant evidence or multiple hospitalizations with discharge diagnoses of psychotic episodes. Cognitive impairment was assessed in a subset of participants who completed a comprehensive neuropsychological test battery, as described previously^49^. Cognitive test data from the Wechsler Adult Intelligence Scale, the Delis-Kaplan Executive Function System, and the Rey Complex Figure Test were clustered, and data from controls were used to estimate the mean and standard deviation of cognitive performance. Cases scoring more than 1.96 standard deviations below the control mean on the first two principal components were classified as cognitively impaired. Extended details are available in the Supplementary Material and Fig. S3. Functional impairment was defined as described above, requiring long-term sick leave in at least 80% of the observation period. Individuals receiving old-age pension were excluded from this analysis.

#### Modeling transdiagnostic contrasts

Multivariate logistic regression models penalized by L1 regularization were trained to predict binary transdiagnostic contrasts (psychotic experience, cognitive impairment, functional impairment) in cases and controls combined. The dataset included all synaptic proteins, NfL, CSF/serum albumin quotient, and complement components previously related to synaptic remodeling (C1q, C3, C3b/iC3c, C4, C4b)^5,48,53^ or previously reported altered in CSF in related disorders (C5)^24,54^. A repeated (*n* = 10) five-by-five-fold nested cross-validation framework was used to maximize generalizability. The penalty parameter (*λ*) was optimized for AUC with equal weight across classes in the inner loop. Model performance was evaluated in the outer loop and summarized as mean (sd) AUC statistics across 50 validation sets (10 repeats x 5 outer folds), with case–case and case–control predictions reported separately. Within each outer loop, recursive feature elimination was applied to the top 25 predictors from the L1-regularized model to reduce the predictor set to two biomarkers. Selection frequency and mean coefficients were extracted across folds. The final LAMP1:NPTX2 ratio was informed by the cross-validation feature selection, but evaluated on the full dataset. AUC estimates should therefore be interpreted with caution.

#### Association with polygenic scores

A subset of participants was genotyped as part of other cohorts using various genotyping arrays (Illumina GSA-MD, PsychChip, OmniExpress, and Affymetrix 6.0). Polygenic scores (PGS) were calculated using the PRS-CS algorithm, as previously described^55^.

### Reporting summary

Further information on research design is available in the Nature Portfolio Reporting Summary linked to this article.

## Supplementary information

Supplementary Information

Transparent Peer Review file

Reporting Summary

## Source data

Source Data

## Data Availability

Source data are provided with this paper. Summarized data are provided in the Supplementary Tables. Access to individual-level research data is regulated by specific national legislation in addition to general data protection laws and is controlled by the University of Gothenburg, which oversees all data access requests. Qualified academic researchers may request access to pseudonymized data for replication purposes, subject to legal review by the University of Gothenburg. Requests for data access should be directed to the principal investigator (ML). Source data are provided with this paper.

## Code availability

Analysis code is available on GitHub^56^.
