# Supplementary material for: Cerebrospinal fluid biomarkers reveal transdiagnostic synaptic dysfunction across major psychiatric disorders
Source: Nat Commun. 2026 Jul 30;17:7604. doi: 10.1038/s41467-026-76187-y (PMC13424668; doi:10.1038/s41467-026-76187-y)
Supplement: Supplementary file 1 — Supplementary Information [file 41467_2026_76187_MOESM1_ESM.pdf]

Supplementary Information

Cerebrospinal fluid biomarkers reveal transdiagnostic synaptic dysfunction across major psychiatric disorders

Andreas Göteson*[1,2], Johanna Nilsson[1], Elena Camporesi[1], Anna Luisa Klahn[1], Elin Hörbeck[1,3,9], Robert Sigström[1,2], Lina Jonsson[1], Timea Sparding[1], Erik Pålsson[1], Aurimantas Pelanis[1], Anneli Goulding[1,3], Anniella Isgren[1,3], Sophie Erhardt[4], Simon Cervenka[5,6], Cynthia M. Bulik[7,8,9], Henrik Zetterberg[1,10,11,12,13,14,15,16], Kaj Blennow[1,10], Carl M. Sellgren[4,6], Ann Brinkmalm[1,10], Mikael Landén[1,9]

*Corresponding author

[1]Department of Psychiatry and Neurochemistry, Institute of Neuroscience and Physiology, Sahlgrenska Academy, University of Gothenburg, Gothenburg, Sweden
[2]Department of Psychiatry for Affective Disorders, Sahlgrenska University Hospital, Gothenburg, Region Västra Götaland, Sweden
[3]Department of Psychotic Disorders, Sahlgrenska University Hospital, Gothenburg, Sweden
[4]Department of Physiology and Pharmacology, Karolinska Institutet, Stockholm, Sweden
[5]Department of Medical Sciences, Psychosis Research and Preventive Psychiatry, Uppsala University, Uppsala, Sweden
[6]Centre for Psychiatry Research, Department of Clinical Neuroscience, Karolinska Institutet & Stockholm Health Care Services, Stockholm, Region Stockholm, Sweden
[7]Department of Psychiatry, University of North Carolina at Chapel Hill, Chapel Hill, NC, USA
[8]Department of Nutrition, University of North Carolina at Chapel Hill, Chapel Hill, NC, USA
[9]Department of Medical Epidemiology and Biostatistics, Karolinska Institutet, Stockholm, Sweden
[10]Clinical Neurochemistry Laboratory, Sahlgrenska University Hospital, Mölndal, Sweden
[11]Department of Neurodegenerative Disease, Dementia Research Centre, UCL Institute of Neurology, Queen Square, London, UK
[12]UK Dementia Research Institute, University College London, London, UK
[13]Hong Kong Center for Neurodegenerative Diseases, Hong Kong, China
[14]Wisconsin Alzheimer's Disease Research Center, University of Wisconsin-Madison, Madison, WI, USA
[15]Department of Pathology and Laboratory Medicine, University of Wisconsin School of Medicine and Public Health, Madison, WI, USA
[16]Centre for Brain Research, Indian Institute of Science, Bangalore, India

Table of Contents

Supplementary Methods 3

Adjusting for non-disease covariance (Figure S1) 3

Definition of cognitive impairment (Figure S3) 4

Supplementary Tables 6

Table S1. Concentration of internal standard, repeatability, and intermediate precision for each peptide from panel 1 and 2 7

Table S2. LC-MS/MS settings for panel 1 and 2, as well as the analyses of SNAP25 and SYT1 8

Table S3. Summary statistics from main case-control analyses 10

Table S4. Variance explained (R2) for each drug adjusted for age, sex, and diagnostic group (and cov_{nd} for synaptic proteins) 16

Table S5. Results from expression quantitative trait loci associations, comparing summary statistics from MetaBrain with results in CSF 20

Table S6. Results from L1-penalized logistic regression models 21

Table S7. Results from recursive feature elimination models 24

Supplementary Figures 25

Figure S2 25

References 26

Supplementary Methods

Adjusting for non-disease covariance (Figure S1)

To account for inter-individual variability in CSF synaptic protein levels, we sought to identify proteins that could serve as a reference for CSF dynamics. We calculated a rank based on five scores. First, a reference protein should not be associated with case-control status. Therefore, we ranked proteins by results from the primary case-control linear regression models, retaining the highest absolute standardized beta value from the five case–control comparisons, where a lower score was preferable. Second, a reference protein should have similar variance in cases and controls. Therefore, we estimated the variance for each protein separately in cases and controls and created a ratio where values close to 1 are preferable, indicating that the variance in cases was similar to controls. Third, a reference protein should closely resemble the main covariance. We captured the main covariance using principal component analyses based on four subgroups: female and male controls at time point 1 and 2. We then averaged the loadings for the first component across the four models to obtain an indicator of proteins with a high degree of correlation to the main covariance in females and males at both time points. Fourth, in the absence of disease, a reference protein should be shed or released into CSF at a stable rate over time within one individual—i.e., most of the variance should originate from between-individual variability rather than within-individual variability over time. We quantified this contrast, commonly referred to as the intraclass correlation coefficient (ICC), in a linear mixed effects model based on longitudinal data from controls. A higher ICC yielded a higher rank. Fifth, we calculated Pearson's correlation coefficients between synapse proteins and amyloid-beta 40, which has previously been suggested to have the characteristics of a reference protein[1]. Amyloid-beta 40 was previously measured in a subset of the participants (n=121 cases and 71 controls)[2]. In a final step, we scaled all five scores using min-max scaling transformed such that a high score always indicates optimal characteristics for a reference protein (i.e., low std. beta, high PC1 loading). We then averaged the five scores to create a rank. The reference for non-disease covariance was computed as a singular value decomposition from the top three most highly ranked candidates (SYUB, STX7, NCAM2) using the NIPALS algorithm. The composite score was highly correlated with the included proteins.

Regressing protein concentrations on this reference and calculating protein-protein correlations revealed that the main covariance was attenuated, yet closely related proteins maintained a high degree of correlation (Figure S1).

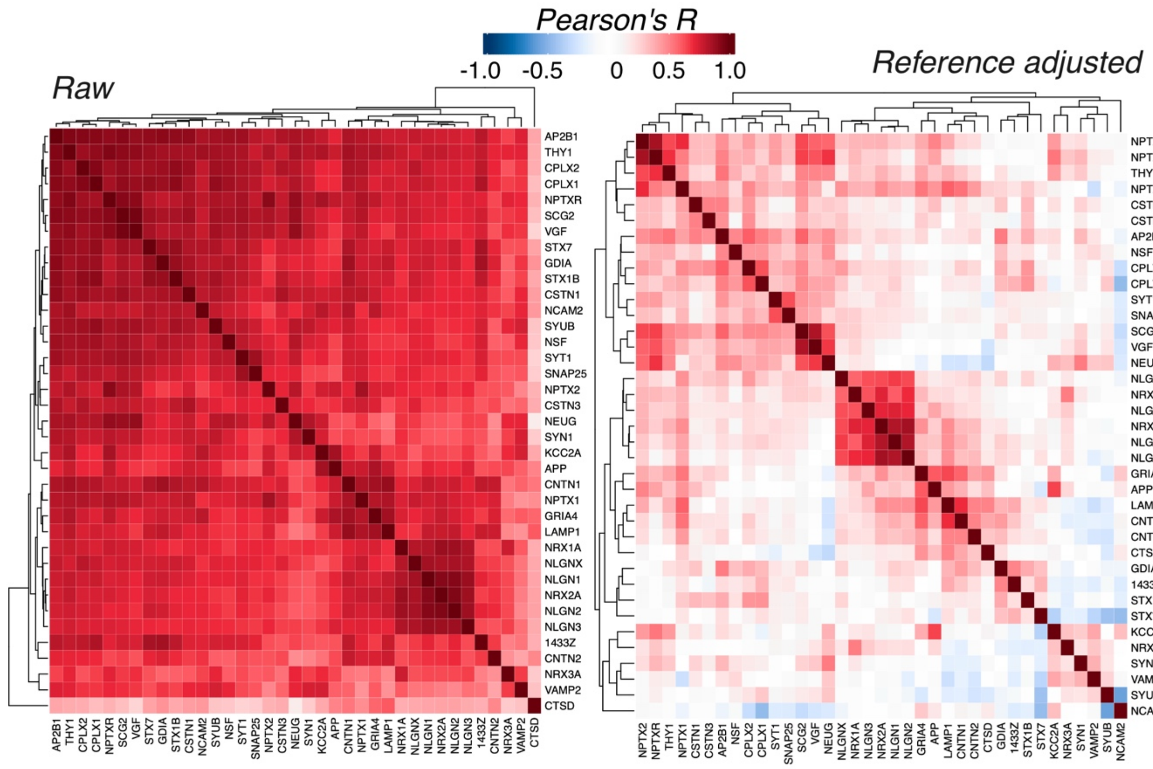

Figure S1. Adjusting for the CSF synapse reference. Heatmaps showing the correlation coefficient for each pairwise protein-protein correlation using raw CSF levels (left) and CSF levels adjusted for the non-disease covariance reference (right).

The effect of expression quantitative trait loci (eQTL's) on CSF protein concentrations was evaluated using a set of high-confidence eQTL's (< 5% false discovery rate) reported by the MetaBrain initiative, using the frontal cortex subset[3]. For each protein available in the synapse panels, we extracted the lead *cis* eQTL variant for the subset of the baseline controls with individual-level genetic data available (n=132). Linear regression models were fitted to evaluate the additive effect of each respective eQTL variant, adjusting for age and sex. This analysis was done using both raw CSF concentrations and adjusted for non-disease covariance. Results are presented in Table S5.

Definition of cognitive impairment (Figure S3)

To identify distinct cognitive domains, data from the cognitive test battery in control participants were subjected to hierarchical clustering using the Ward D2 linkage method. The resulting dendrogram was cut at a height selected by the authors to yield a broad representation of cognitive domains. From each resulting cluster, one representative test score was selected (Figure S3A). These representative tests were then used to perform principal component analysis (PCA) on control data, with unit variance scaling applied. The first two principal components (PC1 and PC2) together explained 45% of the total variance among controls. Euclidean distances in the PC1–PC2 space were computed for controls, and the mean and standard deviation of these distances were used to define a normative range. Data from case participants were projected onto this dimensionality-reduced space, and cases whose projections fell more than 1.96 standard deviations below the control distribution were classified as cognitively impaired (Figure S3B). Scores for each individual cognitive test in participants classified as cognitively normal or cognitively impaired are shown in Figure S3C.

For associations with specific cognitive domains, the scaled FSIQ summary score from WAIS (version 3 or 4) was used to represent full-scale IQ, and executive function, processing speed,

and visual memory were calculated as composite scores using the first principal component of raw scores from representative tests, including:

Executive function: TMT condition 4 (number–letter switching), CWIT conditions 3 (inhibition) and 4 (inhibition/switching), and VFT condition 3 (category switching accuracy).

Processing speed: RCFT time to copy, TMT conditions 2 (number sequencing) and 5 (motor speed), and CWIT conditions 1 (color naming) and 2 (word reading).

Visual memory: RCFT immediate recall and delayed recall.

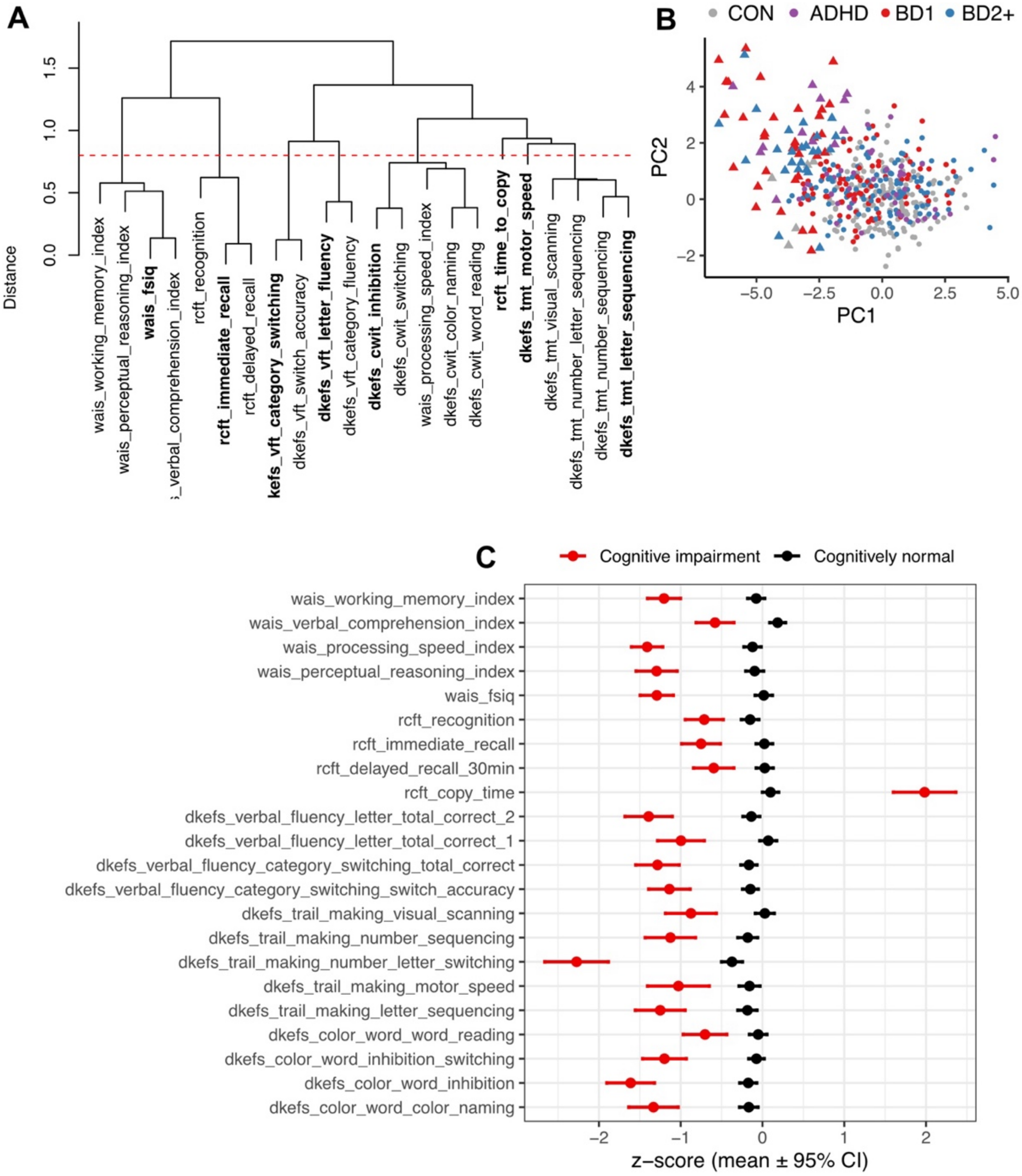

Figure S3. Definition of cognitive impairment. Statistics are based on participants with available cognitive data: n=310 cases, including n=73 classified with 'cognitive impairment', and n=167 controls. A) A dendrogram representation of the hierarchical clustering of cognitive tests in controls. One representative test score was chosen from each cluster, labeled in bold. B) A score plot of the first two principal components (PC1 and PC2) of cognitive scores. Cases labeled 'cognitively impaired' are labeled with triangles. C) Forest plot showing mean values (±95% confidence intervals) for each cognitive test in individuals classified as cognitively impaired or cognitively normal.

Supplementary Tables

Table S1. Concentration of internal standard, repeatability, and intermediate precision for each peptide from panel 1 and 2.

Panel	Protein	Description	Sequence	Internal standard [fmol/µL]	Repeatability (%CV*)	Intermediate precision (%CV*)	Comment
Panel 1	CTSD	Cathepsin D	LSPEDYTLK	1.5	1.5	2.8	
Panel 1	LAMP1	Lysosome-associated membrane glycoprotein 1	ALQATVGNSYK	1.4	1.4	3.3	
Panel 1	VGF	Neurosecretory protein VGF	AYQGVAAPFPK	1.6	1.6	4.1	
Panel 1	SCG2	Secretogranin-2	VLEYLNQEK	1.3	1.3	4.4	
Panel 1	AP2B1	AP-2 complex subunit beta	AVWLPAVK	2.4	2.4	4.6	
Panel 1	CPLX2	Complexin-2	MLGGEEEKDPDAQK	2.6	2.6	4.5	
Panel 1	NPTX1	Neuronal pentraxin-1	LTPGEVYNLATCSTK	4.5	4.5	7.9	
Panel 1	GRIA4	Glutamate receptor 4	IQGLTGNVQFDHYGR	5.2	5.2	9.9	
Panel 1	CNTN2	Contactin-2	VISDTEADIGSNLR	2.2	2.2	4.1	
Panel 1	CSTN1	Calsyntenin-1	GNLAGLTLR	3	3	6.3	
Panel 1	THY1	Thy-1 membrane glycoprotein	VLYLSAFTSK	2.8	2.8	5.7	
Panel 1	APP	Amyloid-beta precursor protein	VESLEQEAANER	6.8	6.8	11.8	
Panel 1	NCAM2	Neural cell adhesion molecule 2	LTIYNANIEDAGIYR	5.6	5.6	12.1	
Panel 1	CSTN3	Calsyntenin-3	GHQPPPEMAGHSLASSHR	6.1	6.1	9.1	
Panel 1	CNTN1	Contactin-1	FIPLIPIPER	3	3	4.5	
Panel 1	SYN1	Synapsin-1	GSHGQTPSPGALPLGR	14.2	14.2	20.9	
Panel 1	NSF	Vesicle-fusing ATPase	AENSSLNLIGK	7.6	7.6	15.4	
Panel 1	CPLX1	Complexin-1	MLGGDEEKDPDAAK	5.1	5.1	7.6	
Panel 1	NPTX2	Neuronal pentraxin-2	VAELEDEK	2.3	2.3	4.7	
Panel 1	NPTXR	Neuronal pentraxin receptor	NNYMYAR	2.1	2.1	3.6	
Panel 1	1433Z	14-3-3 protein zeta/delta	VVSSIEQK	1.8	1.8	3	
Panel 1	STX7	Syntaxin-7	EFGSLPTTPSEQR	6.3	6.3	9.6	
Panel 1	VAMP2	Vesicle-associated membrane protein 2	SATAATAPPAAPAGEGGPPAPPPNLTSNR	9.5	9.5	19.6	
Panel 1	GDIA	Rab GDP dissociation inhibitor alpha	QLICDPSYIPDR	4.8	4.8	8.5	
Panel 1	STX1B	Syntaxin-1B	QHSAILAAPNPDEK	5	5	9.3	
Panel 1	NEUG	Neurogranin	KGPGPGGPGGAGVAR	3.3	3.3	6.7	
Panel 1	SYUB	Beta-synuclein	EGVVQGVASVAEK	5.7	5.7	12.5	
Panel 1	KCC2A	Calcium/calmodulin-dependent protein kinase type II subunit alpha	ITQYLDAGGIPR	7.4	7.4	13.2	
Panel 2	NRX1A	Neurexin-1 α	LTVDDQQAMTGQMAGDHTR	6	6	10	
Panel 2	NRX2A	Neurexin-2 α	LGERPPALLGSQGLR	3.3	3.3	5.1	
Panel 2	NRX3A	Neurexin-3 α	ANDGEWYHVDIQR	10.8	10.8	20.6	
Panel 2	NLGN1	Neuroligin-1	LDDVDPLVATNFGK	2.4	2.4	5.1	
Panel 2	NLGN2	Neuroligin-2	ELVDQDVQPAR	5	5	12	
Panel 2	NLGN3	Neurologin-3	VGCNVLDTVDMVDCLR	5.7	5.7	10.9	
Panel 2	NLGNX	Neuroligin-4	TGPEDTTVLIETK	5.4	5.4	8.6	Common peptides for the two NLGN4 isoforms

*CV coefficient of variation

Table S2. LC-MS/MS settings for panel 1 and 2, as well as the analyses of SNAP25 and SYT1.

Table S2A. LC-MS/MS settings for the analysis of panel 1.

	Parameter	Setting
**LC**	Sample injection volume	40 µL
	Flow-rate	0.3 mL/min
	Gradient	Linear; 5–32%B (42.4 min)
	Total cycle time	45 min
	Mobile phase A	0.1% formic acid in water (v/v)
	Mobile phase B	0.1% formic acid/84% acetonitrile in water (v/v/v)
**Electrospr ay**	Mode	Positive
	Gas temperature	220 °C
	Gas flow	15 L/min
	Nebulizer pressure	40 psi
	Sheath gas temperature	200 °C
	Sheath gas flow	11 L/min
	Capillary voltage	3500 V
	Nozzle voltage	500 V
**iFunnel**	Mode	Positive
	High-pressure radio frequency	200 V
	Low-pressure radio frequency	160 V
**MRM method**	Retention time window	0.8 min
	Collision energies	Individually optimized per transition
	Cell accelerator voltage	Individually optimized per transition

Table S2B. LC-MS/MS settings for the analysis of panel 2.

	Parameter	Setting
**LC**	Sample injection volume	45 µL
	Flow-rate	0.3 mL/min
	Gradient	Linear; 0–32%B (24 min)
	Total cycle time	32 min
	Mobile phase A	0.1% formic acid in water (v/v)
	Mobile phase B	0.1% formic acid/84% acetonitrile in water (v/v/v)
**LC column**	Hypersil Gold reversed-phase column	Particle size 1.9 mm
		Length 100 mm
**Electrospr ay**	Source type	HESI II
	Mode	Positive
	Capillary temperature	320 °C
	Probe heater temperature	300 °C

	Sheath gas flow	25
	Aux gas flow	10
	Spray voltage	4100 V
**PRM method**	Retention time window	2 min
	Collision energies	Individually optimized per transition

Table S2C. LC-MS/MS settings for the analysis of SNAP-25 and synaptotagmin-1.

	**Parameter**	**Setting**
**LC**	Sample injection volume	40 µL
	Flow-rate	0.3 mL/min
	Gradient	Broken; 0–16%B (0.1 min), 16–19%B (11 min), 19–100%B (0.4 min)
	Total cycle time	12.5 min
	Mobile phase A	0.1% formic acid in water (v/v)
	Mobile phase B	0.1% formic acid/84% acetonitrile in water (v/v)
**Electrospray**	Mode	Positive
	Gas temperature	220 °C
	Gas flow	15 L/min
	Nebulizer pressure	30 psi
	Sheath gas temperature	200 °C
	Sheath gas flow	11 L/min
	Capillary voltage	4000 V
	Nozzle voltage	300 V
**iFunnel**	Mode	Positive
	High-pressure radio frequency	120 V
	Low-pressure radio frequency	100 V
**MRM method**	Retention time window	1 min
	Collision energies	Individually optimized per transition
	Cell accelerator voltage	2 V

Table S3. Summary statistics from main case-control analyses

Based on linear regression models adjusted for age, sex, and cov_nd where indicated. P-values are two-sided. FDR indicates the Bejamini-Hochberg implementation of false discovery rate.

		Raw CSF concentrations							*Adjusted for cov_nd*						
**Protein**	**Contrast**	**Beta**	**Std.error**	**95% CI lower**	**95% CI higher**	**t-statistic**	**p-value**	**FDR**	**Beta**	**Std.error**	**95% CI lower**	**95% CI higher**	**t-statistic**	**p-value**	**FDR**
1433Z	ADHD - CON	-0.365	0.124	-0.609	-0.122	-2.937	0.004	0.018	-0.354	0.133	-0.615	-0.094	-2.666	0.008	0.019
1433Z	AN - CON	-0.148	0.192	-0.525	0.229	-0.769	0.444	0.836	-0.576	0.186	-0.941	-0.210	-3.089	0.003	0.038
1433Z	BD1 - CON	-0.249	0.107	-0.459	-0.038	-2.317	0.021	0.035	-0.137	0.124	-0.379	0.105	-1.109	0.268	0.327
1433Z	BD2+ - CON	-0.012	0.100	-0.209	0.184	-0.125	0.901	0.919	-0.145	0.111	-0.362	0.073	-1.300	0.194	0.249
1433Z	SCZ+ - CON	-0.289	0.138	-0.560	-0.019	-2.099	0.037	0.046	0.045	0.161	-0.271	0.361	0.281	0.779	0.795
AP2B1	ADHD - CON	-0.389	0.147	-0.676	-0.101	-2.649	0.009	0.025	-0.514	0.122	-0.753	-0.276	-4.226	3.45e-05	2.61e-04
AP2B1	AN - CON	0.047	0.229	-0.402	0.496	0.205	0.838	0.992	-0.414	0.169	-0.745	-0.083	-2.449	0.016	0.090
AP2B1	BD1 - CON	-0.246	0.117	-0.477	-0.016	-2.098	0.037	0.057	-0.123	0.103	-0.325	0.079	-1.197	0.232	0.290
AP2B1	BD2+ - CON	-0.041	0.113	-0.262	0.181	-0.359	0.720	0.837	-0.317	0.088	-0.489	-0.145	-3.611	3.52e-04	0.001
AP2B1	SCZ+ - CON	-0.755	0.146	-1.040	-0.469	-5.183	4.85e-07	4.04e-06	-1.026	0.131	-1.282	-0.770	-7.858	1.59e-13	2.66e-12
APP	ADHD - CON	-0.294	0.135	-0.558	-0.030	-2.185	0.030	0.057	-0.230	0.128	-0.480	0.021	-1.795	0.074	0.109
APP	AN - CON	0.349	0.208	-0.058	0.757	1.682	0.095	0.581	0.302	0.191	-0.072	0.676	1.585	0.116	0.270
APP	BD1 - CON	-0.553	0.114	-0.777	-0.329	-4.846	2.04e-06	2.04e-05	-0.621	0.114	-0.845	-0.397	-5.437	1.14e-07	8.16e-07
APP	BD2+ - CON	-0.248	0.107	-0.458	-0.038	-2.319	0.021	0.098	-0.474	0.102	-0.674	-0.273	-4.626	5.36e-06	3.83e-05
APP	SCZ+ - CON	-0.560	0.136	-0.826	-0.293	-4.117	5.38e-05	1.79e-04	-0.440	0.131	-0.696	-0.183	-3.362	9.10e-04	0.003
CNTN1	ADHD - CON	-0.439	0.130	-0.694	-0.185	-3.385	8.40e-04	0.006	-0.532	0.140	-0.806	-0.258	-3.805	1.83e-04	0.001
CNTN1	AN - CON	0.003	0.202	-0.393	0.399	0.016	0.987	0.992	-0.360	0.205	-0.763	0.042	-1.757	0.081	0.200
CNTN1	BD1 - CON	-0.314	0.110	-0.529	-0.099	-2.863	0.005	0.009	-0.270	0.118	-0.501	-0.039	-2.292	0.023	0.042
CNTN1	BD2+ - CON	-0.063	0.102	-0.263	0.137	-0.621	0.535	0.743	-0.270	0.104	-0.475	-0.066	-2.589	0.010	0.021
CNTN1	SCZ+ - CON	-0.334	0.127	-0.583	-0.085	-2.627	0.009	0.014	-0.012	0.145	-0.297	0.272	-0.085	0.932	0.932
CNTN2	ADHD - CON	-0.415	0.130	-0.670	-0.160	-3.191	0.002	0.009	-0.388	0.133	-0.649	-0.126	-2.909	0.004	0.012
CNTN2	AN - CON	0.225	0.197	-0.161	0.610	1.141	0.256	0.787	0.108	0.205	-0.294	0.510	0.527	0.599	0.762
CNTN2	BD1 - CON	-0.276	0.107	-0.486	-0.065	-2.571	0.011	0.019	-0.185	0.108	-0.397	0.027	-1.707	0.089	0.135
CNTN2	BD2+ - CON	-0.071	0.103	-0.273	0.131	-0.690	0.491	0.743	-0.182	0.105	-0.387	0.024	-1.735	0.084	0.127
CNTN2	SCZ+ - CON	-0.239	0.127	-0.489	0.010	-1.881	0.061	0.071	0.037	0.130	-0.219	0.292	0.281	0.779	0.795
CPLX1	ADHD - CON	-0.350	0.134	-0.613	-0.087	-2.607	0.010	0.026	-0.429	0.128	-0.680	-0.179	-3.361	9.11e-04	0.004
CPLX1	AN - CON	-0.002	0.215	-0.424	0.420	-0.010	0.992	0.992	-0.484	0.184	-0.845	-0.122	-2.623	0.010	0.080
CPLX1	BD1 - CON	-0.230	0.111	-0.447	-0.013	-2.078	0.039	0.058	-0.112	0.113	-0.334	0.109	-0.993	0.321	0.358
CPLX1	BD2+ - CON	-0.025	0.107	-0.236	0.185	-0.236	0.814	0.888	-0.251	0.096	-0.438	-0.063	-2.622	0.009	0.021
CPLX1	SCZ+ - CON	-0.490	0.138	-0.760	-0.220	-3.560	4.52e-04	9.82e-04	-0.377	0.134	-0.639	-0.115	-2.820	0.005	0.011
CPLX2	ADHD - CON	-0.358	0.145	-0.642	-0.075	-2.476	0.014	0.035	-0.376	0.133	-0.636	-0.117	-2.838	0.005	0.013
CPLX2	AN - CON	-0.051	0.243	-0.526	0.424	-0.210	0.834	0.992	-0.565	0.199	-0.955	-0.175	-2.839	0.005	0.052
CPLX2	BD1 - CON	-0.221	0.125	-0.467	0.025	-1.762	0.079	0.101	-0.055	0.122	-0.295	0.185	-0.450	0.653	0.653
CPLX2	BD2+ - CON	0.026	0.116	-0.202	0.253	0.220	0.826	0.888	-0.119	0.103	-0.321	0.083	-1.157	0.248	0.298
CPLX2	SCZ+ - CON	-0.523	0.150	-0.817	-0.229	-3.488	5.85e-04	0.001	-0.364	0.142	-0.643	-0.086	-2.566	0.011	0.022
CSTN1	ADHD - CON	-0.552	0.152	-0.850	-0.254	-3.627	3.54e-04	0.005	-0.733	0.132	-0.993	-0.474	-5.539	8.43e-08	2.11e-06
CSTN1	AN - CON	0.018	0.240	-0.452	0.489	0.076	0.940	0.992	-0.366	0.205	-0.768	0.036	-1.783	0.077	0.200
CSTN1	BD1 - CON	-0.587	0.127	-0.835	-0.339	-4.633	5.43e-06	3.70e-05	-0.792	0.112	-1.012	-0.572	-7.052	1.27e-11	2.11e-10

CSTN1	BD2+ - CON	-0.191	0.119	-0.424	0.041	-1.611	0.108	0.259	-0.544	0.101	-0.742	-0.345	-5.364	1.53e-07	1.91e-06
CSTN1	SCZ+ - CON	-0.836	0.148	-1.127	-0.545	-5.628	5.38e-08	6.73e-07	-0.965	0.138	-1.236	-0.695	-7.001	2.86e-11	3.58e-10
CSTN3	ADHD - CON	-0.417	0.151	-0.713	-0.120	-2.750	0.006	0.021	-0.375	0.128	-0.626	-0.123	-2.920	0.004	0.012
CSTN3	AN - CON	-0.063	0.232	-0.518	0.392	-0.272	0.786	0.992	-0.402	0.191	-0.776	-0.027	-2.102	0.038	0.142
CSTN3	BD1 - CON	-0.484	0.124	-0.727	-0.241	-3.903	1.18e-04	3.27e-04	-0.473	0.105	-0.678	-0.267	-4.516	9.13e-06	3.26e-05
CSTN3	BD2+ - CON	-0.087	0.120	-0.322	0.148	-0.725	0.469	0.732	-0.256	0.094	-0.440	-0.072	-2.724	0.007	0.016
CSTN3	SCZ+ - CON	-0.697	0.151	-0.993	-0.401	-4.618	6.50e-06	4.06e-05	-0.565	0.130	-0.819	-0.310	-4.346	2.10e-05	1.05e-04
CTSD	ADHD - CON	-0.816	0.134	-1.079	-0.553	-6.075	5.21e-09	2.61e-07	-0.785	0.138	-1.055	-0.515	-5.695	3.82e-08	1.91e-06
CTSD	AN - CON	-0.350	0.203	-0.748	0.049	-1.721	0.088	0.581	-0.492	0.202	-0.889	-0.095	-2.430	0.017	0.090
CTSD	BD1 - CON	-0.191	0.151	-0.486	0.105	-1.262	0.208	0.231	-0.100	0.151	-0.396	0.196	-0.660	0.510	0.531
CTSD	BD2+ - CON	-0.109	0.119	-0.343	0.126	-0.908	0.364	0.634	-0.162	0.121	-0.398	0.075	-1.339	0.182	0.239
CTSD	SCZ+ - CON	-0.586	0.140	-0.861	-0.311	-4.171	4.32e-05	1.54e-04	-0.441	0.148	-0.732	-0.150	-2.972	0.003	0.007
GDIA	ADHD - CON	-0.291	0.133	-0.551	-0.031	-2.192	0.029	0.057	-0.234	0.128	-0.485	0.017	-1.825	0.069	0.105
GDIA	AN - CON	0.183	0.215	-0.237	0.604	0.854	0.395	0.787	-0.024	0.179	-0.376	0.327	-0.137	0.892	0.910
GDIA	BD1 - CON	-0.142	0.110	-0.357	0.073	-1.295	0.196	0.223	0.105	0.101	-0.093	0.303	1.039	0.300	0.357
GDIA	BD2+ - CON	0.140	0.106	-0.069	0.349	1.316	0.189	0.411	0.140	0.101	-0.058	0.339	1.388	0.166	0.224
GDIA	SCZ+ - CON	-0.319	0.138	-0.590	-0.048	-2.304	0.022	0.029	0.065	0.138	-0.206	0.335	0.470	0.639	0.720
GRIA4	ADHD - CON	-0.366	0.140	-0.640	-0.092	-2.616	0.009	0.026	-0.330	0.148	-0.620	-0.040	-2.227	0.027	0.048
GRIA4	AN - CON	0.242	0.216	-0.181	0.665	1.122	0.264	0.787	0.109	0.212	-0.305	0.524	0.516	0.607	0.762
GRIA4	BD1 - CON	-0.433	0.122	-0.672	-0.195	-3.566	4.23e-04	0.001	-0.432	0.126	-0.679	-0.186	-3.442	6.61e-04	0.002
GRIA4	BD2+ - CON	-0.238	0.111	-0.455	-0.020	-2.141	0.033	0.118	-0.508	0.109	-0.720	-0.295	-4.671	4.36e-06	3.63e-05
GRIA4	SCZ+ - CON	-0.883	0.140	-1.157	-0.610	-6.328	1.32e-09	3.30e-08	-0.940	0.153	-1.239	-0.640	-6.149	3.49e-09	2.91e-08
KCC2A	ADHD - CON	-0.254	0.140	-0.528	0.021	-1.810	0.072	0.112	-0.172	0.126	-0.419	0.075	-1.367	0.173	0.222
KCC2A	AN - CON	0.205	0.204	-0.194	0.604	1.009	0.315	0.787	0.078	0.182	-0.278	0.434	0.430	0.668	0.780
KCC2A	BD1 - CON	-0.469	0.116	-0.696	-0.241	-4.042	6.77e-05	2.15e-04	-0.523	0.112	-0.742	-0.303	-4.667	4.65e-06	1.94e-05
KCC2A	BD2+ - CON	-0.276	0.109	-0.489	-0.063	-2.539	0.012	0.097	-0.564	0.102	-0.763	-0.365	-5.551	5.83e-08	9.71e-07
KCC2A	SCZ+ - CON	-0.606	0.144	-0.888	-0.323	-4.203	3.80e-05	1.46e-04	-0.535	0.138	-0.806	-0.264	-3.873	1.41e-04	5.57e-04
LAMP1	ADHD - CON	-0.432	0.125	-0.676	-0.188	-3.465	6.33e-04	0.005	-0.443	0.135	-0.707	-0.179	-3.291	0.001	0.004
LAMP1	AN - CON	0.107	0.197	-0.279	0.492	0.541	0.589	0.963	-0.092	0.201	-0.486	0.301	-0.460	0.647	0.774
LAMP1	BD1 - CON	-0.220	0.108	-0.431	-0.009	-2.045	0.042	0.060	-0.096	0.111	-0.313	0.122	-0.863	0.389	0.422
LAMP1	BD2+ - CON	-0.076	0.097	-0.266	0.115	-0.777	0.438	0.730	-0.224	0.100	-0.420	-0.028	-2.239	0.026	0.048
LAMP1	SCZ+ - CON	-0.319	0.121	-0.556	-0.081	-2.630	0.009	0.014	-0.039	0.133	-0.300	0.222	-0.293	0.770	0.795
NCAM2	ADHD - CON	-0.238	0.126	-0.485	0.009	-1.886	0.061	0.098	-0.163	0.126	-0.410	0.084	-1.291	0.198	0.247
NCAM2	AN - CON	0.157	0.187	-0.209	0.523	0.842	0.402	0.787	-0.085	0.154	-0.387	0.218	-0.549	0.584	0.762
NCAM2	BD1 - CON	-0.280	0.108	-0.491	-0.069	-2.596	0.010	0.018	-0.262	0.106	-0.470	-0.054	-2.473	0.014	0.027
NCAM2	BD2+ - CON	-0.019	0.099	-0.213	0.174	-0.198	0.843	0.888	-0.255	0.091	-0.434	-0.076	-2.798	0.005	0.014
NCAM2	SCZ+ - CON	-0.382	0.128	-0.633	-0.131	-2.984	0.003	0.005	-0.125	0.121	-0.361	0.112	-1.033	0.303	0.369
NEUG	ADHD - CON	-0.305	0.148	-0.594	-0.015	-2.064	0.040	0.069	-0.236	0.109	-0.450	-0.022	-2.157	0.032	0.053
NEUG	AN - CON	0.079	0.228	-0.368	0.526	0.348	0.728	0.991	-0.155	0.164	-0.477	0.168	-0.940	0.349	0.534
NEUG	BD1 - CON	-0.272	0.117	-0.500	-0.043	-2.328	0.021	0.035	-0.172	0.088	-0.345	9.80e-04	-1.949	0.052	0.082
NEUG	BD2+ - CON	-0.021	0.112	-0.240	0.198	-0.186	0.852	0.888	-0.147	0.082	-0.308	0.014	-1.787	0.075	0.118
NEUG	SCZ+ - CON	-0.593	0.149	-0.886	-0.301	-3.981	9.27e-05	2.73e-04	-0.474	0.117	-0.704	-0.244	-4.038	7.39e-05	3.36e-04
NLGN1	ADHD - CON	-0.458	0.132	-0.717	-0.199	-3.461	6.44e-04	0.005	-0.496	0.146	-0.782	-0.210	-3.396	8.08e-04	0.004

NLGN1	AN - CON	-0.091	0.217	-0.517	0.334	-0.421	0.674	0.991	-0.371	0.201	-0.766	0.024	-1.842	0.068	0.196
NLGN1	BD1 - CON	-0.611	0.121	-0.848	-0.374	-5.052	7.71e-07	1.28e-05	-0.678	0.131	-0.935	-0.421	-5.177	4.20e-07	2.62e-06
NLGN1	BD2+ - CON	-0.262	0.114	-0.486	-0.038	-2.295	0.022	0.098	-0.507	0.126	-0.754	-0.260	-4.027	7.02e-05	2.93e-04
NLGN1	SCZ+ - CON	-0.467	0.141	-0.744	-0.191	-3.312	0.001	0.002	-0.238	0.151	-0.534	0.059	-1.571	0.118	0.163
NLGN2	ADHD - CON	-0.379	0.132	-0.638	-0.120	-2.864	0.005	0.018	-0.371	0.148	-0.660	-0.082	-2.513	0.013	0.028
NLGN2	AN - CON	0.086	0.217	-0.341	0.512	0.394	0.695	0.991	-0.092	0.201	-0.486	0.302	-0.459	0.647	0.774
NLGN2	BD1 - CON	-0.567	0.121	-0.804	-0.330	-4.694	4.13e-06	3.44e-05	-0.607	0.133	-0.868	-0.346	-4.566	7.35e-06	2.83e-05
NLGN2	BD2+ - CON	-0.245	0.110	-0.460	-0.029	-2.226	0.027	0.103	-0.471	0.120	-0.706	-0.236	-3.922	1.07e-04	4.11e-04
NLGN2	SCZ+ - CON	-0.533	0.141	-0.810	-0.256	-3.774	2.05e-04	5.13e-04	-0.339	0.156	-0.645	-0.033	-2.171	0.031	0.056
NLGN3	ADHD - CON	-0.228	0.131	-0.484	0.028	-1.748	0.082	0.117	-0.136	0.142	-0.414	0.142	-0.958	0.339	0.385
NLGN3	AN - CON	0.028	0.214	-0.391	0.446	0.130	0.897	0.992	-0.150	0.192	-0.526	0.227	-0.779	0.437	0.649
NLGN3	BD1 - CON	-0.536	0.118	-0.767	-0.305	-4.552	7.86e-06	4.37e-05	-0.553	0.126	-0.801	-0.305	-4.375	1.70e-05	5.67e-05
NLGN3	BD2+ - CON	-0.147	0.110	-0.363	0.070	-1.330	0.185	0.411	-0.294	0.119	-0.527	-0.060	-2.468	0.014	0.027
NLGN3	SCZ+ - CON	-0.328	0.137	-0.596	-0.059	-2.392	0.018	0.024	-0.066	0.145	-0.350	0.218	-0.457	0.648	0.720
NLGNX	ADHD - CON	-0.272	0.133	-0.532	-0.012	-2.050	0.041	0.069	-0.194	0.129	-0.446	0.058	-1.505	0.134	0.186
NLGNX	AN - CON	-0.299	0.221	-0.732	0.133	-1.356	0.178	0.726	-0.692	0.195	-1.074	-0.310	-3.551	5.53e-04	0.027
NLGNX	BD1 - CON	-0.455	0.113	-0.676	-0.234	-4.039	6.88e-05	2.15e-04	-0.431	0.110	-0.647	-0.215	-3.909	1.15e-04	3.03e-04
NLGNX	BD2+ - CON	-0.065	0.109	-0.280	0.149	-0.598	0.550	0.744	-0.190	0.107	-0.400	0.019	-1.783	0.076	0.118
NLGNX	SCZ+ - CON	-0.432	0.141	-0.709	-0.155	-3.059	0.002	0.004	-0.182	0.143	-0.462	0.099	-1.267	0.207	0.258
NPTX1	ADHD - CON	-0.459	0.142	-0.738	-0.180	-3.227	0.001	0.009	-0.440	0.133	-0.701	-0.180	-3.312	0.001	0.004
NPTX1	AN - CON	0.027	0.226	-0.416	0.470	0.121	0.904	0.992	-0.245	0.209	-0.654	0.165	-1.171	0.244	0.413
NPTX1	BD1 - CON	-0.534	0.123	-0.775	-0.293	-4.340	1.96e-05	8.91e-05	-0.547	0.113	-0.768	-0.325	-4.840	2.10e-06	1.05e-05
NPTX1	BD2+ - CON	-0.252	0.120	-0.487	-0.017	-2.100	0.036	0.122	-0.502	0.110	-0.717	-0.287	-4.579	6.63e-06	4.15e-05
NPTX1	SCZ+ - CON	-0.783	0.146	-1.068	-0.497	-5.378	1.88e-07	1.88e-06	-0.701	0.142	-0.978	-0.423	-4.946	1.48e-06	9.26e-06
NPTX2	ADHD - CON	-0.530	0.149	-0.823	-0.238	-3.556	4.58e-04	0.005	-0.542	0.127	-0.790	-0.293	-4.272	2.85e-05	2.61e-04
NPTX2	AN - CON	0.209	0.223	-0.229	0.646	0.935	0.351	0.787	0.067	0.165	-0.257	0.391	0.405	0.686	0.782
NPTX2	BD1 - CON	-0.736	0.123	-0.977	-0.495	-5.978	6.52e-09	3.26e-07	-0.830	0.110	-1.046	-0.614	-7.538	5.95e-13	2.97e-11
NPTX2	BD2+ - CON	-0.394	0.115	-0.618	-0.169	-3.434	6.71e-04	0.017	-0.657	0.095	-0.843	-0.471	-6.920	2.35e-11	5.88e-10
NPTX2	SCZ+ - CON	-1.040	0.141	-1.317	-0.764	-7.385	2.90e-12	1.45e-10	-1.078	0.122	-1.318	-0.838	-8.803	3.53e-16	1.76e-14
NPTXR	ADHD - CON	-0.400	0.149	-0.692	-0.109	-2.690	0.008	0.024	-0.454	0.125	-0.699	-0.209	-3.632	3.48e-04	0.002
NPTXR	AN - CON	0.222	0.229	-0.227	0.671	0.969	0.335	0.787	0.063	0.170	-0.270	0.397	0.371	0.711	0.792
NPTXR	BD1 - CON	-0.556	0.123	-0.798	-0.315	-4.508	9.44e-06	4.72e-05	-0.766	0.108	-0.977	-0.555	-7.113	8.68e-12	2.11e-10
NPTXR	BD2+ - CON	-0.281	0.116	-0.508	-0.054	-2.421	0.016	0.098	-0.738	0.096	-0.926	-0.550	-7.701	1.58e-13	7.88e-12
NPTXR	SCZ+ - CON	-0.851	0.146	-1.138	-0.564	-5.811	2.10e-08	3.50e-07	-1.052	0.127	-1.301	-0.804	-8.295	9.81e-15	2.45e-13
NRX1A	ADHD - CON	-0.324	0.132	-0.584	-0.065	-2.449	0.015	0.036	-0.274	0.133	-0.536	-0.013	-2.055	0.041	0.066
NRX1A	AN - CON	0.017	0.216	-0.406	0.441	0.080	0.936	0.992	-0.194	0.166	-0.519	0.130	-1.174	0.243	0.413
NRX1A	BD1 - CON	-0.602	0.119	-0.835	-0.369	-5.069	7.13e-07	1.28e-05	-0.647	0.115	-0.873	-0.422	-5.621	4.44e-08	3.70e-07
NRX1A	BD2+ - CON	-0.207	0.111	-0.425	0.010	-1.866	0.063	0.166	-0.409	0.107	-0.619	-0.198	-3.808	1.67e-04	5.95e-04
NRX1A	SCZ+ - CON	-0.618	0.143	-0.898	-0.339	-4.334	2.21e-05	1.00e-04	-0.468	0.140	-0.743	-0.194	-3.349	9.51e-04	0.003
NRX2A	ADHD - CON	-0.387	0.134	-0.649	-0.124	-2.889	0.004	0.018	-0.365	0.151	-0.660	-0.069	-2.419	0.016	0.034
NRX2A	AN - CON	-0.035	0.220	-0.466	0.397	-0.157	0.875	0.992	-0.272	0.199	-0.661	0.118	-1.368	0.174	0.355
NRX2A	BD1 - CON	-0.621	0.125	-0.865	-0.377	-4.986	1.06e-06	1.32e-05	-0.667	0.133	-0.927	-0.408	-5.036	8.32e-07	4.62e-06
NRX2A	BD2+ - CON	-0.286	0.118	-0.516	-0.055	-2.427	0.016	0.098	-0.525	0.126	-0.773	-0.277	-4.155	4.15e-05	2.07e-04

NRX2A	SCZ+ - CON	-0.639	0.144	-0.921	-0.358	-4.453	1.33e-05	6.80e-05	-0.497	0.157	-0.805	-0.189	-3.159	0.002	0.005
NRX3A	ADHD - CON	-0.094	0.142	-0.372	0.184	-0.662	0.509	0.591	0.035	0.148	-0.256	0.326	0.238	0.812	0.846
NRX3A	AN - CON	0.284	0.218	-0.143	0.711	1.305	0.194	0.733	0.238	0.195	-0.145	0.621	1.219	0.225	0.413
NRX3A	BD1 - CON	-0.481	0.113	-0.703	-0.259	-4.243	2.96e-05	1.23e-04	-0.472	0.118	-0.704	-0.240	-3.986	8.51e-05	2.50e-04
NRX3A	BD2+ - CON	-0.220	0.115	-0.445	0.005	-1.917	0.056	0.156	-0.373	0.116	-0.599	-0.146	-3.222	0.001	0.004
NRX3A	SCZ+ - CON	-0.597	0.147	-0.885	-0.308	-4.057	6.84e-05	2.14e-04	-0.483	0.153	-0.783	-0.184	-3.163	0.002	0.005
NSF	ADHD - CON	-0.357	0.151	-0.653	-0.061	-2.367	0.019	0.041	-0.326	0.144	-0.607	-0.044	-2.269	0.024	0.045
NSF	AN - CON	0.004	0.216	-0.420	0.428	0.018	0.986	0.992	-0.332	0.178	-0.680	0.016	-1.869	0.064	0.196
NSF	BD1 - CON	-0.125	0.121	-0.362	0.112	-1.033	0.303	0.322	0.105	0.106	-0.102	0.312	0.992	0.322	0.358
NSF	BD2+ - CON	0.082	0.110	-0.133	0.298	0.749	0.455	0.732	0.017	0.095	-0.169	0.203	0.175	0.861	0.897
NSF	SCZ+ - CON	-0.494	0.144	-0.776	-0.213	-3.438	6.97e-04	0.001	-0.288	0.133	-0.548	-0.027	-2.164	0.032	0.056
SCG2	ADHD - CON	-0.082	0.147	-0.371	0.206	-0.559	0.577	0.655	0.199	0.127	-0.051	0.448	1.561	0.120	0.171
SCG2	AN - CON	0.273	0.219	-0.157	0.702	1.245	0.216	0.754	0.206	0.175	-0.137	0.549	1.179	0.241	0.413
SCG2	BD1 - CON	-0.461	0.114	-0.684	-0.238	-4.059	6.33e-05	2.15e-04	-0.636	0.107	-0.845	-0.427	-5.970	6.82e-09	8.53e-08
SCG2	BD2+ - CON	-0.103	0.114	-0.325	0.120	-0.902	0.368	0.634	-0.387	0.107	-0.597	-0.178	-3.621	3.39e-04	0.001
SCG2	SCZ+ - CON	-0.610	0.143	-0.891	-0.329	-4.252	3.11e-05	1.29e-04	-0.640	0.131	-0.896	-0.384	-4.900	1.83e-06	1.02e-05
SNAP25	ADHD - CON	0.070	0.137	-0.199	0.339	0.510	0.611	0.679	0.364	0.134	0.102	0.627	2.725	0.007	0.017
SNAP25	AN - CON	0.182	0.213	-0.236	0.600	0.855	0.394	0.787	0.046	0.197	-0.340	0.432	0.234	0.816	0.850
SNAP25	BD1 - CON	-0.258	0.115	-0.483	-0.033	-2.247	0.025	0.041	-0.171	0.116	-0.398	0.057	-1.471	0.142	0.192
SNAP25	BD2+ - CON	-0.077	0.115	-0.302	0.149	-0.666	0.506	0.743	-0.253	0.122	-0.492	-0.014	-2.079	0.038	0.064
SNAP25	SCZ+ - CON	-0.420	0.141	-0.695	-0.144	-2.987	0.003	0.005	-0.219	0.138	-0.489	0.051	-1.587	0.114	0.163
STX1B	ADHD - CON	-0.282	0.137	-0.552	-0.013	-2.053	0.041	0.069	-0.255	0.140	-0.528	0.019	-1.825	0.069	0.105
STX1B	AN - CON	0.141	0.211	-0.273	0.554	0.667	0.506	0.919	-0.123	0.182	-0.480	0.234	-0.674	0.501	0.723
STX1B	BD1 - CON	-0.230	0.113	-0.451	-0.009	-2.040	0.042	0.060	-0.110	0.108	-0.322	0.102	-1.015	0.311	0.358
STX1B	BD2+ - CON	0.009	0.104	-0.195	0.213	0.087	0.931	0.931	-0.162	0.095	-0.348	0.023	-1.714	0.087	0.129
STX1B	SCZ+ - CON	-0.513	0.134	-0.775	-0.252	-3.844	1.57e-04	4.14e-04	-0.431	0.129	-0.684	-0.177	-3.333	0.001	0.003
STX7	ADHD - CON	-0.300	0.127	-0.548	-0.052	-2.371	0.019	0.041	-0.375	0.136	-0.643	-0.108	-2.754	0.006	0.016
STX7	AN - CON	0.092	0.192	-0.285	0.469	0.478	0.634	0.970	-0.303	0.172	-0.641	0.035	-1.758	0.081	0.200
STX7	BD1 - CON	-0.140	0.104	-0.344	0.064	-1.346	0.179	0.209	0.141	0.109	-0.073	0.354	1.293	0.197	0.252
STX7	BD2+ - CON	0.036	0.098	-0.156	0.229	0.367	0.714	0.837	-0.118	0.099	-0.311	0.075	-1.201	0.231	0.288
STX7	SCZ+ - CON	-0.380	0.128	-0.631	-0.128	-2.956	0.003	0.006	-0.125	0.142	-0.403	0.154	-0.878	0.381	0.453
SYN1	ADHD - CON	-0.343	0.149	-0.635	-0.051	-2.305	0.022	0.046	-0.291	0.134	-0.553	-0.029	-2.173	0.031	0.053
SYN1	AN - CON	0.080	0.227	-0.364	0.524	0.355	0.723	0.991	-0.173	0.179	-0.525	0.179	-0.965	0.336	0.532
SYN1	BD1 - CON	-0.079	0.119	-0.312	0.153	-0.670	0.504	0.525	0.168	0.104	-0.035	0.371	1.623	0.106	0.155
SYN1	BD2+ - CON	0.072	0.116	-0.154	0.299	0.625	0.532	0.743	0.002	0.096	-0.187	0.190	0.019	0.985	0.985
SYN1	SCZ+ - CON	-0.525	0.157	-0.831	-0.218	-3.350	9.48e-04	0.002	-0.337	0.173	-0.676	0.003	-1.942	0.053	0.083
SYT1	ADHD - CON	0.196	0.139	-0.076	0.469	1.414	0.159	0.220	0.676	0.133	0.414	0.937	5.068	8.45e-07	1.41e-05
SYT1	AN - CON	0.503	0.220	0.071	0.936	2.283	0.024	0.395	0.631	0.209	0.222	1.040	3.021	0.003	0.038
SYT1	BD1 - CON	-0.223	0.119	-0.456	0.009	-1.883	0.061	0.082	-0.106	0.126	-0.354	0.142	-0.839	0.402	0.428
SYT1	BD2+ - CON	0.045	0.118	-0.185	0.276	0.383	0.702	0.837	-0.062	0.131	-0.318	0.194	-0.474	0.636	0.691
SYT1	SCZ+ - CON	-0.552	0.142	-0.830	-0.274	-3.895	1.30e-04	3.60e-04	-0.445	0.138	-0.715	-0.176	-3.239	0.001	0.004
SYUB	ADHD - CON	0.004	0.139	-0.268	0.276	0.031	0.975	0.983	0.510	0.121	0.273	0.747	4.212	3.65e-05	2.61e-04
SYUB	AN - CON	0.317	0.217	-0.108	0.741	1.462	0.146	0.692	0.364	0.172	0.027	0.701	2.119	0.036	0.142

SYUB	BD1 - CON	-0.134	0.114	-0.356	0.089	-1.174	0.242	0.263	0.138	0.102	-0.061	0.338	1.362	0.174	0.229
SYUB	BD2+ - CON	0.204	0.106	-0.003	0.412	1.927	0.055	0.156	0.368	0.091	0.190	0.547	4.053	6.30e-05	2.86e-04
SYUB	SCZ+ - CON	-0.244	0.139	-0.517	0.029	-1.750	0.081	0.093	0.257	0.126	0.009	0.505	2.034	0.043	0.074
THY1	ADHD - CON	-0.406	0.140	-0.680	-0.133	-2.911	0.004	0.018	-0.599	0.136	-0.866	-0.333	-4.416	1.56e-05	1.95e-04
THY1	AN - CON	0.050	0.215	-0.371	0.471	0.234	0.816	0.992	-0.393	0.185	-0.756	-0.030	-2.122	0.036	0.142
THY1	BD1 - CON	-0.325	0.116	-0.552	-0.098	-2.808	0.005	0.010	-0.363	0.118	-0.594	-0.131	-3.072	0.002	0.005
THY1	BD2+ - CON	-0.121	0.109	-0.335	0.093	-1.107	0.269	0.517	-0.534	0.102	-0.734	-0.334	-5.241	2.85e-07	2.85e-06
THY1	SCZ+ - CON	-0.611	0.137	-0.880	-0.342	-4.449	1.36e-05	6.80e-05	-0.709	0.135	-0.973	-0.445	-5.255	3.43e-07	2.45e-06
VAMP2	ADHD - CON	-0.180	0.141	-0.455	0.095	-1.281	0.202	0.265	-0.043	0.118	-0.274	0.187	-0.367	0.714	0.760
VAMP2	AN - CON	0.184	0.211	-0.230	0.599	0.872	0.385	0.787	0.037	0.157	-0.270	0.345	0.238	0.813	0.850
VAMP2	BD1 - CON	-0.025	0.117	-0.255	0.204	-0.215	0.830	0.830	0.194	0.094	0.010	0.379	2.068	0.040	0.068
VAMP2	BD2+ - CON	0.045	0.107	-0.165	0.254	0.417	0.677	0.837	-0.027	0.088	-0.199	0.146	-0.301	0.763	0.812
VAMP2	SCZ+ - CON	-0.543	0.151	-0.838	-0.247	-3.602	3.89e-04	8.84e-04	-0.382	0.135	-0.648	-0.117	-2.823	0.005	0.011
VGF	ADHD - CON	-0.031	0.143	-0.311	0.249	-0.219	0.827	0.861	0.295	0.124	0.051	0.539	2.372	0.019	0.037
VGF	AN - CON	0.354	0.218	-0.073	0.781	1.626	0.107	0.581	0.388	0.180	0.036	0.741	2.158	0.033	0.142
VGF	BD1 - CON	-0.459	0.111	-0.677	-0.242	-4.141	4.53e-05	1.74e-04	-0.626	0.109	-0.840	-0.411	-5.719	2.63e-08	2.63e-07
VGF	BD2+ - CON	-0.136	0.110	-0.352	0.080	-1.231	0.219	0.457	-0.445	0.103	-0.647	-0.244	-4.335	1.94e-05	1.08e-04
VGF	SCZ+ - CON	-0.700	0.141	-0.977	-0.424	-4.966	1.35e-06	9.63e-06	-0.838	0.133	-1.098	-0.578	-6.313	1.43e-09	1.43e-08
									The reference was developed for synaptic proteins; results below are shown for transparency						
C1q	ADHD - CON	-0.052	0.144	-0.334	0.231	-0.359	0.720	0.783	-0.005	0.149	-0.297	0.287	-0.034	0.973	0.973
C1q	AN - CON	-0.168	0.188	-0.536	0.200	-0.894	0.373	0.787	-0.224	0.190	-0.596	0.148	-1.181	0.240	0.413
C1q	BD1 - CON	0.183	0.108	-0.028	0.394	1.701	0.090	0.113	0.235	0.109	0.021	0.449	2.155	0.032	0.057
C1q	BD2+ - CON	0.196	0.102	-0.004	0.396	1.918	0.056	0.156	0.171	0.105	-0.036	0.377	1.622	0.106	0.151
C1q	SCZ+ - CON	-0.033	0.145	-0.318	0.252	-0.225	0.822	0.822	0.052	0.150	-0.242	0.346	0.346	0.730	0.793
C2	ADHD - CON	0.518	0.135	0.254	0.782	3.845	1.56e-04	0.004	0.574	0.140	0.300	0.849	4.100	5.75e-05	3.59e-04
C2	AN - CON	0.098	0.192	-0.278	0.474	0.511	0.610	0.965	0.058	0.196	-0.326	0.442	0.295	0.768	0.837
C2	BD1 - CON	0.030	0.108	-0.181	0.241	0.279	0.781	0.797	0.069	0.110	-0.147	0.286	0.626	0.532	0.543
C2	BD2+ - CON	-0.040	0.096	-0.229	0.149	-0.416	0.678	0.837	-0.066	0.099	-0.259	0.128	-0.664	0.507	0.577
C2	SCZ+ - CON	-0.302	0.141	-0.577	-0.027	-2.149	0.033	0.042	-0.253	0.143	-0.533	0.028	-1.764	0.079	0.120
C3	ADHD - CON	-0.282	0.131	-0.539	-0.024	-2.146	0.033	0.061	-0.306	0.133	-0.567	-0.045	-2.298	0.022	0.043
C3	AN - CON	-0.522	0.205	-0.923	-0.121	-2.552	0.012	0.292	-0.507	0.208	-0.914	-0.100	-2.444	0.016	0.090
C3	BD1 - CON	-0.326	0.106	-0.533	-0.119	-3.084	0.002	0.005	-0.346	0.107	-0.555	-0.137	-3.247	0.001	0.003
C3	BD2+ - CON	-0.393	0.101	-0.591	-0.194	-3.884	1.24e-04	0.006	-0.376	0.102	-0.575	-0.177	-3.698	2.55e-04	8.50e-04
C3	SCZ+ - CON	-0.551	0.151	-0.847	-0.255	-3.648	3.27e-04	7.79e-04	-0.592	0.153	-0.892	-0.292	-3.865	1.45e-04	5.57e-04
C3b/iC3b	ADHD - CON	0.039	0.120	-0.195	0.273	0.326	0.745	0.793	0.095	0.125	-0.150	0.339	0.760	0.448	0.498
C3b/iC3b	AN - CON	-0.284	0.167	-0.610	0.043	-1.701	0.092	0.581	-0.333	0.167	-0.661	-0.005	-1.990	0.049	0.159
C3b/iC3b	BD1 - CON	0.351	0.114	0.129	0.574	3.091	0.002	0.005	0.399	0.117	0.169	0.628	3.408	7.47e-04	0.002
C3b/iC3b	BD2+ - CON	0.171	0.106	-0.037	0.379	1.608	0.109	0.259	0.160	0.110	-0.056	0.377	1.453	0.147	0.204
C3b/iC3b	SCZ+ - CON	-0.316	0.132	-0.574	-0.058	-2.400	0.017	0.024	-0.266	0.136	-0.531	-1.74e-04	-1.961	0.051	0.082
C4	ADHD - CON	0.131	0.134	-0.131	0.393	0.980	0.328	0.417	0.134	0.135	-0.131	0.398	0.991	0.323	0.375
C4	AN - CON	-0.108	0.194	-0.489	0.273	-0.554	0.581	0.963	-0.114	0.194	-0.495	0.267	-0.587	0.558	0.759
C4	BD1 - CON	0.174	0.113	-0.046	0.395	1.549	0.123	0.146	0.180	0.113	-0.041	0.401	1.599	0.111	0.158
C4	BD2+ - CON	0.122	0.104	-0.081	0.326	1.177	0.240	0.480	0.112	0.104	-0.093	0.317	1.072	0.284	0.331

C4	SCZ+ - CON	-0.090	0.141	-0.366	0.187	-0.635	0.526	0.537	-0.094	0.142	-0.372	0.184	-0.662	0.509	0.592
C4b	ADHD - CON	0.100	0.134	-0.163	0.362	0.744	0.458	0.558	0.158	0.139	-0.114	0.430	1.138	0.256	0.307
C4b	AN - CON	-0.382	0.205	-0.783	0.019	-1.868	0.064	0.581	-0.431	0.212	-0.846	-0.016	-2.036	0.044	0.153
C4b	BD1 - CON	0.317	0.109	0.104	0.531	2.910	0.004	0.008	0.361	0.111	0.143	0.578	3.255	0.001	0.003
C4b	BD2+ - CON	0.239	0.105	0.033	0.444	2.276	0.023	0.098	0.222	0.107	0.013	0.431	2.077	0.039	0.064
C4b	SCZ+ - CON	0.145	0.145	-0.140	0.430	0.999	0.319	0.332	0.217	0.147	-0.071	0.504	1.479	0.141	0.190
C5	ADHD - CON	0.132	0.136	-0.135	0.399	0.969	0.334	0.417	0.158	0.140	-0.115	0.432	1.134	0.258	0.307
C5	AN - CON	-0.253	0.177	-0.600	0.094	-1.430	0.155	0.692	-0.271	0.179	-0.622	0.080	-1.513	0.133	0.296
C5	BD1 - CON	0.495	0.107	0.285	0.706	4.614	5.92e-06	3.70e-05	0.513	0.109	0.300	0.725	4.722	3.62e-06	1.64e-05
C5	BD2+ - CON	0.306	0.101	0.108	0.504	3.024	0.003	0.045	0.287	0.102	0.088	0.487	2.821	0.005	0.013
C5	SCZ+ - CON	0.271	0.141	-0.005	0.547	1.927	0.055	0.066	0.281	0.142	0.003	0.560	1.981	0.049	0.081
C5a	ADHD - CON	0.103	0.145	-0.180	0.386	0.712	0.477	0.568	0.098	0.145	-0.185	0.382	0.681	0.497	0.540
C5a	AN - CON	-0.591	0.187	-0.958	-0.224	-3.155	0.002	0.099	-0.584	0.186	-0.949	-0.218	-3.130	0.002	0.038
C5a	BD1 - CON	0.235	0.118	0.004	0.466	1.992	0.047	0.066	0.229	0.117	-0.002	0.459	1.947	0.053	0.082
C5a	BD2+ - CON	0.119	0.113	-0.102	0.340	1.058	0.291	0.538	0.128	0.111	-0.090	0.347	1.152	0.250	0.298
C5a	SCZ+ - CON	-0.379	0.140	-0.653	-0.105	-2.707	0.007	0.011	-0.428	0.138	-0.698	-0.158	-3.106	0.002	0.005
CFH	ADHD - CON	0.003	0.141	-0.274	0.280	0.021	0.983	0.983	0.014	0.142	-0.263	0.292	0.100	0.921	0.940
CFH	AN - CON	0.013	0.210	-0.399	0.425	0.062	0.951	0.992	0.015	0.210	-0.397	0.427	0.073	0.942	0.942
CFH	BD1 - CON	0.176	0.111	-0.040	0.393	1.594	0.112	0.137	0.174	0.111	-0.043	0.390	1.573	0.117	0.162
CFH	BD2+ - CON	-0.058	0.107	-0.267	0.150	-0.549	0.584	0.768	-0.054	0.107	-0.264	0.156	-0.506	0.613	0.681
CFH	SCZ+ - CON	-0.173	0.141	-0.450	0.104	-1.222	0.223	0.237	-0.183	0.142	-0.462	0.096	-1.283	0.201	0.257
CFI	ADHD - CON	0.376	0.137	0.109	0.644	2.755	0.006	0.021	0.391	0.138	0.121	0.660	2.840	0.005	0.013
CFI	AN - CON	-0.187	0.178	-0.535	0.161	-1.052	0.295	0.787	-0.191	0.178	-0.540	0.158	-1.074	0.285	0.466
CFI	BD1 - CON	0.431	0.109	0.217	0.645	3.945	1.00e-04	2.94e-04	0.434	0.109	0.220	0.649	3.969	9.09e-05	2.53e-04
CFI	BD2+ - CON	0.226	0.097	0.035	0.417	2.323	0.021	0.098	0.206	0.097	0.016	0.395	2.122	0.035	0.062
CFI	SCZ+ - CON	0.237	0.138	-0.033	0.507	1.718	0.087	0.097	0.221	0.138	-0.049	0.490	1.602	0.111	0.163
MBL	ADHD - CON	0.193	0.140	-0.081	0.467	1.381	0.169	0.228	0.198	0.140	-0.077	0.473	1.412	0.159	0.210
MBL	AN - CON	0.121	0.206	-0.282	0.524	0.589	0.557	0.963	0.128	0.205	-0.275	0.531	0.624	0.534	0.747
MBL	BD1 - CON	0.368	0.112	0.148	0.587	3.284	0.001	0.003	0.363	0.112	0.143	0.583	3.238	0.001	0.003
MBL	BD2+ - CON	0.288	0.105	0.082	0.494	2.745	0.006	0.064	0.268	0.104	0.064	0.473	2.579	0.010	0.021
MBL	SCZ+ - CON	0.360	0.153	0.061	0.659	2.357	0.019	0.026	0.335	0.154	0.034	0.636	2.181	0.030	0.056
NfL	ADHD - CON	0.148	0.084	-0.016	0.312	1.770	0.078	0.115	0.280	0.097	0.090	0.470	2.888	0.004	0.012
NfL	AN - CON	0.253	0.118	0.022	0.483	2.147	0.034	0.413	0.199	0.141	-0.077	0.475	1.410	0.161	0.343
NfL	BD1 - CON	0.252	0.078	0.100	0.404	3.249	0.001	0.003	0.395	0.093	0.212	0.577	4.232	3.08e-05	9.63e-05
NfL	BD2+ - CON	0.191	0.065	0.063	0.319	2.927	0.004	0.046	0.203	0.079	0.049	0.357	2.576	0.010	0.021
NfL	SCZ+ - CON	0.244	0.120	0.010	0.479	2.045	0.042	0.051	0.487	0.141	0.211	0.764	3.453	6.60e-04	0.002
QAlb	ADHD - CON	-0.219	0.124	-0.462	0.023	-1.772	0.078	0.115	-0.189	0.127	-0.439	0.060	-1.489	0.138	0.186
QAlb*	AN - CON	N/A	N/A	N/A	N/A	N/A	N/A	N/A	N/A	N/A	N/A	N/A	N/A	N/A	N/A
QAlb	BD1 - CON	0.209	0.114	-0.014	0.432	1.839	0.067	0.088	0.234	0.116	0.006	0.462	2.008	0.046	0.076
QAlb	BD2+ - CON	0.030	0.109	-0.183	0.244	0.278	0.781	0.888	0.007	0.110	-0.209	0.223	0.061	0.951	0.971
QAlb	SCZ+ - CON	-0.245	0.153	-0.544	0.054	-1.607	0.109	0.119	-0.216	0.155	-0.521	0.089	-1.390	0.166	0.218

*QAlb was not measured in AN

Table S4. Variance explained (R2) for each drug adjusted for age, sex, and diagnostic group (and cov_nd for synaptic proteins).
Based on linear regression models. P-values are two-sided. FDR indicates the Bejamini-Hochberg implementation of false discovery rate.

Protein	Drug	n exposed	rsq	p-value	FDR
1433Z	AC	136	7.24E-04	0.464	0.816
1433Z	AD	191	0.001	0.338	0.71
1433Z	AP	151	6.07E-04	0.483	0.816
1433Z	CS	81	5.00E-04	0.543	0.854
1433Z	Li	191	0.003	0.145	0.533
ALBQ	AC	123	0.001	0.382	0.755
ALBQ	AD	174	0.001	0.386	0.755
ALBQ	AP	139	1.93E-04	0.71	0.939
ALBQ	CS	74	3.96E-04	0.594	0.895
ALBQ	Li	165	0.01	0.007	0.111
AP2B1	AC	136	1.73E-04	0.728	0.944
AP2B1	AD	190	1.73E-04	0.728	0.944
AP2B1	AP	150	7.00E-04	0.471	0.816
AP2B1	CS	81	8.41E-04	0.442	0.796
AP2B1	Li	191	0.007	0.022	0.196
APP	AC	136	6.23E-05	0.838	0.97
APP	AD	191	7.26E-05	0.826	0.969
APP	AP	151	0.003	0.161	0.537
APP	CS	81	1.41E-08	0.998	0.998
APP	Li	191	7.59E-04	0.476	0.816
C1q	AC	136	3.21E-04	0.644	0.911
C1q	AD	188	0.001	0.381	0.755
C1q	AP	153	1.01E-04	0.789	0.953
C1q	CS	82	0.005	0.077	0.402
C1q	Li	186	0.004	0.106	0.448
C2	AC	136	0.003	0.14	0.53
C2	AD	188	0.002	0.24	0.616
C2	AP	153	0.003	0.125	0.498
C2	CS	82	2.96E-04	0.638	0.911
C2	Li	186	0.01	0.007	0.111
C3	AC	136	5.33E-04	0.554	0.854
C3	AD	188	0.003	0.158	0.537
C3	AP	153	6.37E-05	0.833	0.97
C3	CS	82	3.17E-04	0.648	0.911
C3	Li	186	0.005	0.069	0.367
C3b_iC3c	AC	136	0.001	0.386	0.755
C3b_iC3c	AD	188	0.003	0.171	0.537
C3b_iC3c	AP	153	9.20E-06	0.931	0.996
C3b_iC3c	CS	82	0.002	0.186	0.553
C3b_iC3c	Li	186	1.09E-05	0.929	0.996
C4	AC	136	2.27E-04	0.705	0.938
C4	AD	188	0.004	0.106	0.448
C4	AP	153	0.003	0.183	0.552
C4	CS	82	0.003	0.139	0.53
C4	Li	186	0.003	0.159	0.537
C4b	AC	136	0.002	0.24	0.616
C4b	AD	188	0.005	0.052	0.319
C4b	AP	153	2.26E-04	0.678	0.938
C4b	CS	82	3.55E-05	0.875	0.996
C4b	Li	186	0.017	5.27E-04	0.013
C5	AC	136	3.15E-04	0.622	0.908
C5	AD	188	0.003	0.116	0.485
C5	AP	153	0.005	0.043	0.284
C5	CS	82	2.24E-06	0.967	0.996
C5	Li	186	0.009	0.009	0.112
C5a	AC	136	0.001	0.335	0.71
C5a	AD	188	0.001	0.333	0.71
C5a	AP	153	3.16E-04	0.623	0.908
C5a	CS	82	1.15E-06	0.977	0.996
C5a	Li	186	3.07E-06	0.963	0.996
CFH	AC	136	9.27E-04	0.449	0.801

CFH	AD	188	9.80E-05	0.805	0.961
CFH	AP	153	0.002	0.203	0.576
CFH	CS	82	2.46E-04	0.696	0.938
CFH	Li	186	0.002	0.224	0.608
CFI	AC	136	1.16E-04	0.772	0.953
CFI	AD	188	1.20E-04	0.768	0.953
CFI	AP	153	0.005	0.055	0.328
CFI	CS	82	1.49E-04	0.742	0.947
CFI	Li	186	0.009	0.012	0.134
CNTN1	AC	136	4.98E-04	0.551	0.854
CNTN1	AD	190	0.002	0.295	0.661
CNTN1	AP	150	0.001	0.289	0.661
CNTN1	CS	81	0.003	0.126	0.498
CNTN1	Li	191	6.05E-04	0.511	0.844
CNTN2	AC	136	0.007	0.025	0.208
CNTN2	AD	191	6.13E-04	0.521	0.85
CNTN2	AP	151	0.004	0.1	0.44
CNTN2	CS	81	2.24E-05	0.902	0.996
CNTN2	Li	191	0.006	0.04	0.268
CPLX1	AC	136	0.008	0.026	0.208
CPLX1	AD	191	0.003	0.166	0.537
CPLX1	AP	151	2.05E-05	0.907	0.996
CPLX1	CS	81	0.002	0.31	0.685
CPLX1	Li	191	0.04	3.79E-07	3.16E-05
CPLX2	AC	136	0.006	0.047	0.298
CPLX2	AD	191	0.003	0.131	0.512
CPLX2	AP	151	1.01E-05	0.932	0.996
CPLX2	CS	81	0.004	0.081	0.407
CPLX2	Li	191	0.047	8.08E-09	2.02E-06
CSTN1	AC	136	2.43E-04	0.664	0.927
CSTN1	AD	191	0.003	0.145	0.533
CSTN1	AP	151	0.005	0.048	0.298
CSTN1	CS	81	6.31E-04	0.482	0.816
CSTN1	Li	191	0.001	0.289	0.661
CSTN3	AC	136	0.001	0.336	0.71
CSTN3	AD	191	1.25E-04	0.758	0.953
CSTN3	AP	151	0.01	0.005	0.091
CSTN3	CS	81	0.003	0.162	0.537
CSTN3	Li	191	0.009	0.008	0.112
CTSD	AC	136	7.63E-07	0.982	0.996
CTSD	AD	191	0.003	0.197	0.572
CTSD	AP	151	3.39E-07	0.988	0.996
CTSD	CS	81	0.001	0.403	0.768
CTSD	Li	191	0.009	0.012	0.134
GDIA	AC	136	6.88E-06	0.946	0.996
GDIA	AD	191	0.017	8.02E-04	0.018
GDIA	AP	151	3.00E-04	0.647	0.911
GDIA	CS	81	1.30E-05	0.926	0.996
GDIA	Li	191	5.54E-04	0.547	0.854
GRIA4	AC	136	0.002	0.296	0.661
GRIA4	AD	191	6.51E-04	0.505	0.842
GRIA4	AP	151	0.018	3.25E-04	0.009
GRIA4	CS	81	2.21E-04	0.698	0.938
GRIA4	Li	191	0.002	0.285	0.661
KCC2A	AC	136	0.002	0.239	0.616
KCC2A	AD	191	0.001	0.37	0.747
KCC2A	AP	151	1.67E-05	0.911	0.996
KCC2A	CS	81	0.001	0.37	0.747
KCC2A	Li	191	1.04E-06	0.979	0.996
LAMP1	AC	136	5.43E-04	0.53	0.854
LAMP1	AD	191	7.32E-05	0.817	0.969
LAMP1	AP	151	4.68E-04	0.548	0.854
LAMP1	CS	81	2.94E-07	0.988	0.996
LAMP1	Li	191	0.001	0.356	0.735
MBL	AC	136	8.71E-04	0.455	0.807

MBL	AD	188	1.83E-05	0.914	0.996
MBL	AP	153	0.002	0.281	0.661
MBL	CS	82	6.11E-05	0.843	0.971
MBL	Li	186	0.001	0.343	0.714
NCAM2	AC	136	2.54E-06	0.968	0.996
NCAM2	AD	190	1.68E-06	0.974	0.996
NCAM2	AP	150	0.005	0.063	0.361
NCAM2	CS	81	0.002	0.245	0.619
NCAM2	Li	191	0.007	0.039	0.267
NEUG	AC	136	4.43E-04	0.539	0.854
NEUG	AD	191	2.76E-05	0.878	0.996
NEUG	AP	151	6.49E-04	0.441	0.796
NEUG	CS	81	0.002	0.241	0.616
NEUG	Li	191	4.24E-06	0.952	0.996
NLGN1	AC	136	0.009	0.013	0.137
NLGN1	AD	190	0.002	0.215	0.591
NLGN1	AP	150	1.07E-04	0.784	0.953
NLGN1	CS	81	2.58E-04	0.681	0.938
NLGN1	Li	190	3.17E-04	0.649	0.911
NLGN2	AC	136	0.005	0.079	0.404
NLGN2	AD	190	0.005	0.066	0.365
NLGN2	AP	150	2.29E-04	0.691	0.938
NLGN2	CS	81	6.58E-04	0.513	0.844
NLGN2	Li	190	1.24E-04	0.777	0.953
NLGN3	AC	134	8.36E-06	0.942	0.996
NLGN3	AD	188	0.002	0.251	0.621
NLGN3	AP	149	7.26E-05	0.824	0.969
NLGN3	CS	80	2.43E-05	0.901	0.996
NLGN3	Li	189	0.002	0.273	0.661
NLGNX	AC	136	0.001	0.294	0.661
NLGNX	AD	190	0.001	0.286	0.661
NLGNX	AP	150	9.05E-05	0.784	0.953
NLGNX	CS	81	4.71E-07	0.985	0.996
NLGNX	Li	190	8.25E-04	0.425	0.787
NPTX1	AC	136	4.99E-04	0.564	0.859
NPTX1	AD	190	6.54E-05	0.835	0.97
NPTX1	AP	150	0.005	0.069	0.367
NPTX1	CS	81	1.55E-05	0.919	0.996
NPTX1	Li	191	1.90E-04	0.722	0.944
NPTX2	AC	136	0.001	0.312	0.685
NPTX2	AD	191	0.001	0.295	0.661
NPTX2	AP	151	0.003	0.084	0.412
NPTX2	CS	81	3.97E-05	0.856	0.982
NPTX2	Li	191	0.008	0.012	0.134
NPTXR	AC	136	0.004	0.058	0.336
NPTXR	AD	191	1.72E-04	0.701	0.938
NPTXR	AP	151	0.003	0.125	0.498
NPTXR	CS	81	8.43E-05	0.788	0.953
NPTXR	Li	191	0.009	0.007	0.111
NRX1A	AC	136	0.003	0.151	0.537
NRX1A	AD	190	8.78E-04	0.441	0.796
NRX1A	AP	150	0.003	0.178	0.542
NRX1A	CS	81	2.22E-04	0.698	0.938
NRX1A	Li	190	0.003	0.16	0.537
NRX2A	AC	136	0.003	0.164	0.537
NRX2A	AD	190	0.004	0.1	0.44
NRX2A	AP	150	0.001	0.405	0.768
NRX2A	CS	81	3.51E-06	0.962	0.996
NRX2A	Li	190	0.003	0.174	0.537
NRX3A	AC	136	3.79E-04	0.608	0.899
NRX3A	AD	190	5.41E-04	0.54	0.854
NRX3A	AP	150	0.002	0.193	0.568
NRX3A	CS	81	1.45E-04	0.751	0.953
NRX3A	Li	190	7.37E-04	0.474	0.816
NSF	AC	136	0.005	0.086	0.412

NSF	AD	191	8.17E-04	0.47	0.816
NSF	AP	151	2.11E-04	0.704	0.938
NSF	CS	81	4.33E-04	0.598	0.896
NSF	Li	191	0.028	1.75E-05	8.75E-04
NfL	AC	138	0.003	0.033	0.243
NfL	AD	193	8.38E-06	0.914	0.996
NfL	AP	154	0.004	0.02	0.181
NfL	CS	82	0.001	0.172	0.537
NfL	Li	192	0.003	0.037	0.267
SCG2	AC	136	0.004	0.093	0.433
SCG2	AD	191	0.006	0.029	0.223
SCG2	AP	151	0.003	0.092	0.433
SCG2	CS	81	8.38E-04	0.42	0.783
SCG2	Li	191	0.035	1.59E-07	1.98E-05
SNAP25	AC	132	9.36E-05	0.807	0.961
SNAP25	AD	185	0.001	0.368	0.747
SNAP25	AP	147	4.86E-04	0.567	0.859
SNAP25	CS	79	7.91E-08	0.994	0.998
SNAP25	Li	188	0.002	0.247	0.619
STX1B	AC	136	0.008	0.027	0.215
STX1B	AD	191	9.63E-04	0.433	0.796
STX1B	AP	151	3.13E-06	0.963	0.996
STX1B	CS	81	4.15E-04	0.607	0.899
STX1B	Li	191	0.025	6.32E-05	0.002
STX7	AC	136	1.60E-04	0.737	0.945
STX7	AD	191	0.01	0.009	0.112
STX7	AP	151	0.002	0.172	0.537
STX7	CS	81	1.44E-05	0.92	0.996
STX7	Li	191	0.001	0.325	0.706
SYN1	AC	136	0.002	0.212	0.591
SYN1	AD	191	1.71E-04	0.73	0.944
SYN1	AP	151	8.30E-05	0.804	0.961
SYN1	CS	81	0.002	0.276	0.661
SYN1	Li	191	0.024	4.55E-05	0.002
SYT1	AC	132	0.004	0.096	0.437
SYT1	AD	185	3.62E-04	0.624	0.908
SYT1	AP	147	6.25E-04	0.505	0.842
SYT1	CS	79	3.43E-04	0.634	0.911
SYT1	Li	188	0.001	0.411	0.772
SYUB	AC	136	1.61E-04	0.732	0.944
SYUB	AD	191	0.008	0.015	0.141
SYUB	AP	151	9.07E-04	0.403	0.768
SYUB	CS	81	0.002	0.235	0.616
SYUB	Li	191	0.003	0.168	0.537
THY1	AC	136	0.008	0.023	0.196
THY1	AD	190	0.001	0.401	0.768
THY1	AP	150	1.22E-04	0.771	0.953
THY1	CS	81	0.002	0.231	0.616
THY1	Li	191	5.23E-04	0.559	0.857
VAMP2	AC	136	0.023	5.05E-05	0.002
VAMP2	AD	191	5.06E-04	0.552	0.854
VAMP2	AP	151	1.26E-04	0.759	0.953
VAMP2	CS	81	0.002	0.199	0.572
VAMP2	Li	191	0.031	3.10E-06	1.94E-04
VGF	AC	136	1.86E-05	0.905	0.996
VGF	AD	191	0.012	0.003	0.057
VGF	AP	151	0.007	0.014	0.141
VGF	CS	81	0.002	0.215	0.591
VGF	Li	191	0.009	0.009	0.112

Table S5. Results from expression quantitative trait loci associations, comparing summary statistics from MetaBrain with results in CSF.
Based on linear regression models. P-values are two-sided. Proteins with a significant association (nominal p-value < 0.05) are shown

								Raw CSF concentrations		Ajusted for cov_nd	
**Protein**	**Accession**	**Gene_metabrain**	**CHROM**	**POS (GRCh38)**	**rs ID**	**MetaBeta (metabrain)**	**MetaP (metabrain)**	**Std. beta**	**p-value**	**Std. beta**	**p-value**
CNTN2	Q02246	ENSG00000184144.12	1	205071887	rs11240349	0.258	9.44E-22	0.243	0.001	0.353	1.30E-05
CPLX1	O14810	ENSG00000168993.15	4	726719	rs7438761	-0.183	2.48E-10	-0.209	0.01	-0.276	0.001
CPLX2	Q6PUV4	ENSG00000145920.15	5	175877679	rs12522368	0.162	3.22E-09	0.017	0.842	0.168	0.045
NPTX1	Q15818	ENSG00000171246.6	17	80475411	rs74003912	-0.221	1.40E-10	-0.163	0.059	-0.322	2.25E-04
NPTXR	O95502	ENSG00000221890.4	22	38834735	rs1076612	-0.268	7.00E-23	-0.15	0.083	-0.174	0.027
STX7	O15400	ENSG00000079950.14	6	132473636	rs9321345	0.252	5.39E-20	0.039	0.622	0.218	0.009

Table S6. Results from L1-penalized logistic regression models

For each contrast, the top predictive features are shown with their selection frequency and mean coefficients across cross-validation folds.

contrast	feature	n_models_selected	mean_coef	se_coef
psychotic_experience	NPTX2	50	-0.803	0.039
psychotic_experience	C5	50	0.532	0.026
psychotic_experience	ALBQ	49	-0.301	0.023
psychotic_experience	CSTN1	49	-0.535	0.031
psychotic_experience	VGF	49	-0.525	0.037
psychotic_experience	C3	49	-0.138	0.011
psychotic_experience	NEUG	49	0.688	0.041
psychotic_experience	LAMP1	49	0.906	0.036
psychotic_experience	GRIA4	49	-0.453	0.024
psychotic_experience	1433Z	48	-0.305	0.022
psychotic_experience	C3b_iC3c	48	-0.223	0.015
psychotic_experience	CNTN2	46	0.111	0.012
psychotic_experience	C1q	46	0.131	0.012
psychotic_experience	NRX2A	45	-0.211	0.025
psychotic_experience	NLGNX	44	0.175	0.018
psychotic_experience	NfL	44	-0.137	0.016
psychotic_experience	SYT1	41	-0.153	0.021
psychotic_experience	NRX3A	40	-0.105	0.014
psychotic_experience	SNAP25	37	0.193	0.029
psychotic_experience	THY1	36	0.315	0.045
psychotic_experience	CNTN1	36	0.168	0.025
psychotic_experience	NLGN3	35	0.081	0.012
psychotic_experience	NCAM2	30	0.057	0.01
psychotic_experience	C4	30	-0.022	0.007
psychotic_experience	SYN1	29	0.068	0.014
psychotic_experience	SYUB	27	-0.116	0.026
psychotic_experience	NLGN2	23	0.109	0.023
psychotic_experience	NSF	23	0.027	0.01
psychotic_experience	NPTX1	22	-0.034	0.013
psychotic_experience	VAMP2	22	1.00E-02	0.008
psychotic_experience	AP2B1	21	-0.076	0.022
psychotic_experience	CTSD	21	0.007	0.005
psychotic_experience	CPLX1	20	-0.084	0.022
psychotic_experience	KCC2A	20	0.02	0.006
psychotic_experience	C4b	20	-0.01	0.01
psychotic_experience	STX7	17	0.04	0.013
psychotic_experience	CSTN3	17	-0.048	0.014
psychotic_experience	NRX1A	14	-0.051	0.017
psychotic_experience	CPLX2	13	0.053	0.02
psychotic_experience	APP	12	0.003	0.006
psychotic_experience	NPTXR	11	0.008	0.017
psychotic_experience	STX1B	10	0.011	0.009
psychotic_experience	GDIA	10	-0.002	0.006
psychotic_experience	NLGN1	10	0.008	0.009

psychotic_experience	SCG2	8	0.064	0.027
cognitive_impariment	NPTX2	50	-0.915	0.081
cognitive_impariment	C1q	50	0.233	0.021
cognitive_impariment	GDIA	47	0.421	0.052
cognitive_impariment	NfL	46	0.169	0.015
cognitive_impariment	SYUB	37	0.25	0.038
cognitive_impariment	C3b_iC3c	33	0.108	0.02
cognitive_impariment	NRX3A	31	0.291	0.05
cognitive_impariment	C3	29	-0.054	0.011
cognitive_impariment	VAMP2	28	0.084	0.024
cognitive_impariment	SYN1	26	0.155	0.031
cognitive_impariment	LAMP1	26	0.63	0.117
cognitive_impariment	NCAM2	25	-0.337	0.065
cognitive_impariment	SNAP25	25	-0.163	0.033
cognitive_impariment	CSTN1	25	-0.35	0.062
cognitive_impariment	NLGN1	23	-0.249	0.058
cognitive_impariment	STX7	23	0.251	0.055
cognitive_impariment	C4	22	-0.029	0.011
cognitive_impariment	KCC2A	22	-0.044	0.015
cognitive_impariment	NSF	21	0.118	0.031
cognitive_impariment	1433Z	20	-0.163	0.038
cognitive_impariment	NPTX1	16	0.173	0.053
cognitive_impariment	CNTN2	16	-0.046	0.014
cognitive_impariment	APP	15	-0.045	0.016
cognitive_impariment	CTSD	15	-0.056	0.019
cognitive_impariment	C4b	15	-0.035	0.016
cognitive_impariment	SCG2	15	-0.171	0.061
cognitive_impariment	CPLX1	14	-0.288	0.086
cognitive_impariment	GRIA4	13	-0.066	0.024
cognitive_impariment	VGF	13	0.23	0.083
cognitive_impariment	NLGNX	12	0.067	0.029
cognitive_impariment	C5	12	-0.044	0.02
cognitive_impariment	NLGN3	11	0.078	0.026
cognitive_impariment	THY1	10	0.11	0.046
cognitive_impariment	NRX1A	10	-0.081	0.036
cognitive_impariment	SYT1	10	0.011	0.011
cognitive_impariment	CNTN1	9	-0.083	0.03
cognitive_impariment	ALBQ	9	0.002	0.007
cognitive_impariment	CPLX2	8	0.046	0.026
cognitive_impariment	STX1B	7	-0.033	0.024
cognitive_impariment	NEUG	7	-0.019	0.027
cognitive_impariment	CSTN3	7	-0.002	0.012
cognitive_impariment	NRX2A	6	-0.012	0.023
cognitive_impariment	AP2B1	5	0.037	0.021
cognitive_impariment	NPTXR	4	-0.052	0.03
cognitive_impariment	NLGN2	4	0.005	0.016
functional_impairment	CTSD	50	0.264	0.017
functional_impairment	GDIA	50	0.596	0.042

functional_impairment	NfL	50	0.373	0.016
functional_impairment	NPTX2	50	-0.79	0.054
functional_impairment	SNAP25	49	-0.336	0.021
functional_impairment	C5	48	0.252	0.024
functional_impairment	GRIA4	48	-0.39	0.03
functional_impairment	C3	46	-0.118	0.012
functional_impairment	CNTN2	45	0.203	0.021
functional_impairment	SYUB	45	0.462	0.039
functional_impairment	CSTN1	44	-0.492	0.05
functional_impairment	VGF	43	-0.579	0.065
functional_impairment	LAMP1	43	0.462	0.042
functional_impairment	NCAM2	42	0.2	0.022
functional_impairment	ALBQ	41	-0.247	0.024
functional_impairment	VAMP2	41	-0.358	0.037
functional_impairment	NLGNX	40	-0.217	0.025
functional_impairment	C1q	39	0.116	0.015
functional_impairment	THY1	38	0.295	0.039
functional_impairment	NEUG	38	0.412	0.047
functional_impairment	CPLX2	37	-0.492	0.061
functional_impairment	NPTX1	35	0.269	0.037
functional_impairment	APP	35	-0.254	0.034
functional_impairment	NRX3A	34	-0.086	0.018
functional_impairment	C4b	33	-0.101	0.018
functional_impairment	NLGN3	33	0.308	0.043
functional_impairment	SYN1	32	0.094	0.015
functional_impairment	STX1B	32	-0.221	0.035
functional_impairment	C4	31	-0.053	0.011
functional_impairment	NSF	31	0.092	0.016
functional_impairment	CPLX1	29	0.357	0.062
functional_impairment	1433Z	28	-0.054	0.018
functional_impairment	NLGN2	28	-0.172	0.036
functional_impairment	C3b_iC3c	25	0.033	0.01
functional_impairment	NLGN1	24	-0.101	0.029
functional_impairment	KCC2A	23	0.017	0.019
functional_impairment	SYT1	22	0.096	0.023
functional_impairment	CNTN1	22	0.068	0.02
functional_impairment	NRX1A	21	0.156	0.04
functional_impairment	STX7	19	-0.052	0.019
functional_impairment	NPTXR	17	0.176	0.048
functional_impairment	NRX2A	16	-0.069	0.026
functional_impairment	CSTN3	16	0.066	0.022
functional_impairment	AP2B1	14	-0.124	0.036
functional_impairment	SCG2	9	0.01	0.025

Table S7. Results from recursive feature elimination models

For each contrast, the selected pairs are shown with their selection frequency and mean coefficients across cross-validation folds

contrast	feature_1	feature_2	n_models_selected	mean_coef_1	se_coef_1	mean_coef_2	se_coef_2
psychotic_experience	LAMP1	NPTX2	32	0.656	0.017	-1.013	0.021
psychotic_experience	NEUG	VGF	7	0.552	0.035	-0.965	0.046
psychotic_experience	GRIA4	LAMP1	5	-1.184	0.057	0.923	0.066
psychotic_experience	ALBQ	C5	3	-0.704	0.016	0.947	0.031
psychotic_experience	CNTN1	CSTN1	1	0.655	NA	-0.95	NA
psychotic_experience	NLGN2	NRX2A	1	0.511	NA	-0.809	NA
psychotic_experience	THY1	VGF	1	0.692	NA	-1.101	NA
cognitive_impariment	LAMP1	NPTX2	14	0.867	0.054	-1.053	0.053
cognitive_impariment	GDIA	NPTX2	11	0.881	0.057	-1.029	0.062
cognitive_impariment	NPTX2	SYUB	9	-1.069	0.062	0.824	0.042
cognitive_impariment	CSTN1	GDIA	8	-1.121	0.137	1.142	0.14
cognitive_impariment	NPTX2	STX7	3	-1.102	0.08	0.946	0.068
cognitive_impariment	AP2B1	NPTX2	2	1.16	0.009	-1.473	0.047
cognitive_impariment	AP2B1	CSTN1	1	1.676	NA	-1.772	NA
cognitive_impariment	CSTN1	STX7	1	-1.511	NA	1.427	NA
cognitive_impariment	NPTX2	THY1	1	-1.501	NA	1.206	NA
functional_impairment	LAMP1	NPTX2	24	1.09	0.033	-1.218	0.042
functional_impairment	CSTN1	GDIA	7	-1.157	0.168	1.146	0.166
functional_impairment	GRIA4	LAMP1	5	-1.142	0.185	1.162	0.199
functional_impairment	CPLX2	GDIA	4	-1.09	0.065	1.141	0.074
functional_impairment	CPLX1	CPLX2	3	1.281	0.235	-1.241	0.261
functional_impairment	NPTX2	THY1	3	-1.052	0.008	0.895	0.025
functional_impairment	CNTN1	NPTX2	1	1.066	NA	-1.137	NA
functional_impairment	CPLX1	VGF	1	0.787	NA	-0.911	NA
functional_impairment	NfL	SNAP25	1	0.656	NA	-0.344	NA
functional_impairment	SYUB	VGF	1	0.949	NA	-1.06	NA

Supplementary Figures

Figure S2

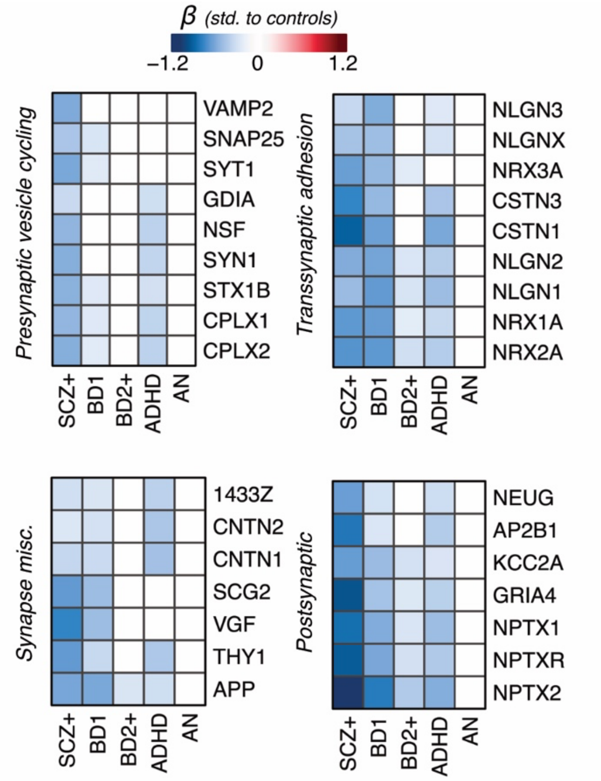

Figure S2. Case–control associations for synaptic proteins using raw CSF levels. Group sizes: controls, n=167; schizophrenia spectrum (SCZ+), n=73; bipolar disorder type 1 (BD1), n=140; bipolar disorder type 2 and related conditions (BD2+), n=179; attention-deficit/hyperactivity disorder (ADHD), n=73; anorexia nervosa (AN), n=40. Heatmaps of effect sizes (β-values, standardized to controls) from linear regression models for each disorder compared with controls, showing associations at <20% false discovery rate. The color scale reflects association strength. This figure mirrors Figure 2A but is based on raw CSF levels and does not display significance markers. Complement and neurodegeneration markers are not shown, as their results are identical to Figure 2A. Complete results are provided in Table S3.

References

1. Karlsson, L. *et al.* Cerebrospinal fluid reference proteins increase accuracy and interpretability of biomarkers for brain diseases. *Nat. Commun.* **15**, 3676 (2024).
2. Jakobsson, J. *et al.* Altered Concentrations of Amyloid Precursor Protein Metabolites in the Cerebrospinal Fluid of Patients with Bipolar Disorder. *Neuropsychopharmacology* **38**, 664–672 (2013).
3. De Klein, N. *et al.* Brain expression quantitative trait locus and network analyses reveal downstream effects and putative drivers for brain-related diseases. *Nat. Genet.* **55**, 377–388 (2023).
