# Supplementary material for: Cerebrospinal fluid biomarkers reveal transdiagnostic synaptic dysfunction across major psychiatric disorders
Source: Nat Commun. 2026 Jul 30;17:7604. doi: 10.1038/s41467-026-76187-y (PMC13424668; doi:10.1038/s41467-026-76187-y)
Supplement: Supplementary file 3 — Reporting Summary [file 41467_2026_76187_MOESM3_ESM.pdf]

nature portfolio

Corresponding author(s): Andreas Göteson

Last updated by author(s): Jun 12, 2026

Reporting Summary

Nature Portfolio wishes to improve the reproducibility of the work that we publish. This form provides structure for consistency and transparency in reporting. For further information on Nature Portfolio policies, see our Editorial Policies and the Editorial Policy Checklist.

Statistics

For all statistical analyses, confirm that the following items are present in the figure legend, table legend, main text, or Methods section.

n/a	Confirmed	
☐	☒	The exact sample size (*n*) for each experimental group/condition, given as a discrete number and unit of measurement
☐	☒	A statement on whether measurements were taken from distinct samples or whether the same sample was measured repeatedly
☐	☒	The statistical test(s) used AND whether they are one- or two-sided<br>*Only common tests should be described solely by name; describe more complex techniques in the Methods section.*
☐	☒	A description of all covariates tested
☐	☒	A description of any assumptions or corrections, such as tests of normality and adjustment for multiple comparisons
☐	☒	A full description of the statistical parameters including central tendency (e.g. means) or other basic estimates (e.g. regression coefficient) AND variation (e.g. standard deviation) or associated estimates of uncertainty (e.g. confidence intervals)
☐	☒	For null hypothesis testing, the test statistic (e.g. *F*, *t*, *r*) with confidence intervals, effect sizes, degrees of freedom and *P* value noted<br>*Give P values as exact values whenever suitable.*
☒	☐	For Bayesian analysis, information on the choice of priors and Markov chain Monte Carlo settings
☐	☒	For hierarchical and complex designs, identification of the appropriate level for tests and full reporting of outcomes
☐	☒	Estimates of effect sizes (e.g. Cohen's *d*, Pearson's *r*), indicating how they were calculated

Our web collection on statistics for biologists contains articles on many of the points above.

Software and code

Policy information about availability of computer code

Data collection	Skyline 20.1 (MacCoss Lab Software) was utilized to analyze the mass spectrometric data
Data analysis	Analysis code is available on GitHub: Göteson A., et al., Cerebrospinal fluid biomarkers reveal transdiagnostic synaptic dysfunction across major psychiatric disorders, https://github.com/andreasgoteson/2025-CSF-synaptic-biomarkers-major-psychiatric-disorders, DOI: 10.5281/zenodo.21332816, 2026.<br><br>The following software was used for statistical analyses: Python 3 (v. 3.13.1) with the following packages: pandas 2.2.3, numpy v.2.2.4, matplotlib v.3.10.1, scikit-learn v.1.6.1. R version 4.4.3 with the following libraries: pROC (v.1.18.5), ComplexHeatmap (v.2.22.0), broom (v.1.0.8), dplyr (v.1.1.4), purr (v.1.0.4), tidyr (v.1.3.1), tibble (v.3.2.1), ggplot2 (v.3.5.2).

For manuscripts utilizing custom algorithms or software that are central to the research but not yet described in published literature, software must be made available to editors and reviewers. We strongly encourage code deposition in a community repository (e.g. GitHub). See the Nature Portfolio guidelines for submitting code & software for further information.

Data

Policy information about availability of data

All manuscripts must include a data availability statement. This statement should provide the following information, where applicable:

- Accession codes, unique identifiers, or web links for publicly available datasets
- A description of any restrictions on data availability
- For clinical datasets or third party data, please ensure that the statement adheres to our policy

Summarized data are provided in the Supplementary Tables. Access to individual-level research data is regulated by specific national legislation in addition to general data protection laws and is controlled by the University of Gothenburg, which oversees all data access requests. Qualified academic researchers may request access to pseudonymized data for replication purposes, subject to legal review by the University of Gothenburg. Requests for data access should be directed to the principal investigator (ML).

Research involving human participants, their data, or biological material

Policy information about studies with human participants or human data. See also policy information about sex, gender (identity/presentation), and sexual orientation and race, ethnicity and racism.

Reporting on sex and gender	The distribution of biological sex across groups is shown in Table 1, based on self-reported information at study inclusion. Sex was entered as a covariate in the case–control models.
Reporting on race, ethnicity, or other socially relevant groupings	We did not collect or report data on race/ethnicity in this study, but race/ethnicity is not expected to be different from the demographic distributions of the general Swedish population, which is mainly of European ancestry.
Population characteristics	Study participant characteristics are detailed in Table 1, including age at sampling, biological sex, body mass index, diagnostic subtype, duration of illness, number of past hospitalizations, psychotic experience, comorbid substance abuse, and current medication.
Recruitment	Recruitment of study participants was detailed in the SBP cohort profile, please refer to DOI:10.1159/000543335. Similarly, details on the external replication sample can be found in DOI: 10.1038/s41467-022-33797-6.
Ethics oversight	The study was approved by the regional ethical review board in Stockholm, Sweden.

Note that full information on the approval of the study protocol must also be provided in the manuscript.

Field-specific reporting

Please select the one below that is the best fit for your research. If you are not sure, read the appropriate sections before making your selection.

[x] Life sciences [ ] Behavioural & social sciences [ ] Ecological, evolutionary & environmental sciences

For a reference copy of the document with all sections, see nature.com/documents/nr-reporting-summary-flat.pdf

Life sciences study design

All studies must disclose on these points even when the disclosure is negative.

Sample size	Sample size was not predetermined but based on sample availability in the included cohorts (we analyzed all samples available at the biobank)
Data exclusions	The total sample included 672 individuals with data from at least two protein analysis methods. During plate preparations, minor technical issues led to insufficient CSF volume for inclusion of all samples across all assays. Missing data were as follows: NfL (n = 3), Synapse Panel 1 (n = 4), Synapse Panel 2 (n = 3), SNAP25 and SYT1 (n = 10), and Complement Panel (n = 15). Measurements of the CSF/serum albumin quotient were performed in parallel efforts and not conducted for individuals from the ANGI cohort, resulting in additional missing values (n = 87; distributed as follows: ADHD, n=1; AN, n=40; BD1, n=19; BD2+, n=23; CON, n=1; SCZ+, n=3). No available data was excluded in statistical analysis.
Replication	Samples were analyzed once with no technical replicates; however, at the group-level, we replicated main findings using an external cohort as detailed in the main text
Randomization	N/A -- our study defined group characteristics based on clinical profile with no experimental exposures
Blinding	Laboratory analysts were blinded to the true diagnostic group of each sample. To enable stratified randomization across plates, they received a file containing limited sample characteristics, including "pseudo-groups" (numerical group labels rather than diagnostic codes). The pseudo-groups were constructed to have approximately equal sizes corresponding to the smallest diagnostic group, to minimize the risk that analysts could infer true group membership. This information was used only to ensure balanced distribution of pseudo-groups across plates.

Reporting for specific materials, systems and methods

We require information from authors about some types of materials, experimental systems and methods used in many studies. Here, indicate whether each material, system or method listed is relevant to your study. If you are not sure if a list item applies to your research, read the appropriate section before selecting a response.

Materials & experimental systems

n/a	Involved in the study
☐	☒ Antibodies
☒	☐ Eukaryotic cell lines
☒	☐ Palaeontology and archaeology
☒	☐ Animals and other organisms
☐	☒ Clinical data
☒	☐ Dual use research of concern
☒	☐ Plants

Methods

n/a	Involved in the study
☒	☐ ChIP-seq
☒	☐ Flow cytometry
☒	☐ MRI-based neuroimaging

Antibodies

Antibodies used

CSF NfL concentration was measured using an in-house enzyme-linked immunosorbent assay, as previously described (DOI: 10.1186/s13195-018-0339-1). Albumin concentrations were analyzed by immunonephelometry on a Beckman Immage Immunochemistry system (Beckman Instruments, Beckman Coulter, Brea, CA, USA). Complement proteins were measured using a multiplexed Luminex MAGPIX panel according to the manufacturer's instructions (Luminex Corp., Austin TX)

Validation

Please refer to above studies for more details

Clinical data

Policy information about clinical studies

All manuscripts should comply with the ICMJE guidelines for publication of clinical research and a completed CONSORT checklist must be included with all submissions.

Clinical trial registration

The SBP cohort is an observational clinical study registered on Researchweb (https://www.researchweb.org/is/vgr/project/39411) on January 1st, 2009 (project ID: 39411). The KaSP cohort is an observational clinical study which was not registered, but more details are provided in previous publications (DOI: 10.1038/s41467-022-33797-6).

Study protocol

N/A -- the clinical data is not from a clinical trial

Data collection

The clinical data collection has been described in detail previously (for the SBP cohort: DOI:10.1159/000543335; for the external replication sample: 10.1038/s41467-022-33797-6)

Outcomes

Diagnostic outcomes were defined according to DSM-IV criteria, please see the cohort profile (DOI:10.1159/000543335). Other clinical outcomes were not predefined in a published study protocol. For the external replication sample, please see DOI:10.1038/s41467-022-33797-6

Plants

Seed stocks

Report on the source of all seed stocks or other plant material used. If applicable, state the seed stock centre and catalogue number. If plant specimens were collected from the field, describe the collection location, date and sampling procedures.

Novel plant genotypes

Describe the methods by which all novel plant genotypes were produced. This includes those generated by transgenic approaches, gene editing, chemical/radiation-based mutagenesis and hybridization. For transgenic lines, describe the transformation method, the number of independent lines analyzed and the generation upon which experiments were performed. For gene-edited lines, describe the editor used, the endogenous sequence targeted for editing, the targeting guide RNA sequence (if applicable) and how the editor was applied.

Authentication

Describe any authentication procedures for each seed stock used or novel genotype generated. Describe any experiments used to assess the effect of a mutation and, where applicable, how potential secondary effects (e.g. second site T-DNA insertions, mosiacism, off-target gene editing) were examined.
