# Supplementary material for: Cerebrospinal fluid biomarkers reveal transdiagnostic synaptic dysfunction across major psychiatric disorders
Source: Nat Commun. 2026 Jul 30;17:7604. doi: 10.1038/s41467-026-76187-y (PMC13424668; doi:10.1038/s41467-026-76187-y)
Supplement: Supplementary file 2 — Transparent Peer Review file [file 41467_2026_76187_MOESM2_ESM.pdf]

nature portfolio

Peer Review File

Cerebrospinal fluid biomarkers reveal transdiagnostic synaptic dysfunction across major psychiatric disorders

Corresponding Author: Mr Andreas Göteson

This file contains all reviewer reports in order by version, followed by all author rebuttals in order by version.

Version 0:

Reviewer comments:

Reviewer #1

(Remarks to the Author)
This study by Göteson et al. used targeted mass spectrometry to measure low-abundance synaptic proteins in CSF from 672 individuals across major psychiatric disorders and controls, revealing most severe changes in schizophrenia, with intermediate ones in bipolar disorder and ADHD. By modeling transdiagnostic clinical features, the authors identified the LAMP1:NPTX2 ratio as a biomarker associated with psychotic experience, cognitive impairment, and functional decline. Findings were externally replicated in first-episode psychosis, supporting early and measurable synaptic pathology across psychiatric conditions. The study utilizes a large cohort, applies sophisticated methods, and supports the concept of synaptic dysfunction as a shared biological substrate across major psychiatric disorders, particularly in schizophrenia and bipolar disorder. However, there are some concerns:
• Uneven diagnostic sampling: Large BD cohorts vs smaller ADHD, AN, and SCZ groups may bias effect estimation. AN cohort is 100% females. Moreover, only a few SCZ participants completed neurocognitive assessments, limiting transdiagnostic inference.
• Case-control comparisons of CSF synapse proteins across disorders: Several proteins were significantly associated with disease but the AUCs ranged from 0.69-0.78, potentially limiting their utility as diagnostic biomarkers. The authors state that there were no significant associations with in BD2+ or AN (Line 172), but Figure 2A shows several significant proteins for BD2+ (e.g. NPTX2). Also, it is unclear if Figure 2A, which is not referred to in the text, includes adjustment for non-disease covariance or not.
• Complement components and neurodegeneration biomarkers as correlates of synaptic dysfunction: “Compared with controls, CSF NfL concentrations were significantly higher in SCZ+ (β [95%CI]=0.24 [0.01, 0.48], P=0.042), BD1 (0.25 [0.10, 0.40], P=0.001), BD2+ (0.19 [0.06, 0.32], P=0.004), and AN (0.25 [0.02, 0.48], P=0.03).” – these results seem inconsistent with Figure 2A.
• CSF biomarker ratio reflecting psychotic liability, cognitive, and functional impairment: Although they were significant, the correlations of LAMP1:NPTX2 ratio with cognitive scores were low (all R^2 < 0.1). Authors state that LAMP1:NPTX2 ratio showed a progressive increase along the ADHD-BD-SCZ spectrum (Figure 3C), but there is no statistical analysis and Figure 3C is not cited in the text.
• Although the authors conclude that the LAMP1:NPTX2 ratio reflects “synaptic dysfunction as a transdiagnostic feature,” this interpretation is limited by missing data in key diagnostic groups (“Participants with AN were excluded due to missing clinical data, and most individuals with SCZ+ had not undergone cognitive testing”).
• Medication confounding: Though variance explained was small (~1%), most participants were medicated at CSF draw, especially in BD and SCZ.
• LAMP1 was not available in the replication cohort, which limited replication.
• Figures are incorrectly cited or not cited, making it difficult to follow the manuscript, e.g.:
o Figure 1A, 1E are not referred to in the text, and 1D is cited after 1F.
o Line 126 R^2 range: 35–79% doesn’t match Figure 1C.
o Line 182, 237: incorrect figure citations.

Reviewer #2

(Remarks to the Author)
This study by Göteson et al demonstrates that synaptic dysfunction is a measurable and transdiagnostic feature of major psychiatric disorders. The paper presents a comprehensive analysis by quantifying 37 synaptic proteins using mass

spectrometry across large and clinically diverse cohorts with schizophrenia, bipolar disorder, ADHD, and anorexia nervosa. This broad investigation of a large protein panel across multiple distinct psychiatric conditions represents a significant advance in the search for transdiagnostic biomarkers. The paper identifies a novel ratio of two cerebrospinal fluid proteins, LAMP1 and NPTX2, as a robust marker. This ratio correlates with psychotic experiences, cognitive deficits, and functional impairment across different diagnostic categories. While NPTX2 and LAMP1 have been studied in other neurological contexts, their specific ratio as a proxy for synaptic pathology in major psychiatric disorders is a new finding.
The paper makes several interesting claims that appear well-founded.
1) Synaptic protein concentrations are markedly reduced in schizophrenia spectrum disorders, with intermediate reductions in bipolar disorder and ADHD. Schizophrenia spectrum shows consistent, sizable reductions across many synaptic proteins (notably NPTX2 and GRIA4), while bipolar disorder and ADHD exhibit fewer and smaller changes, consistent with an 'intermediate' pattern. Did the authors test if NPTX2 and GRIA4 are correlated in CSF?
2) The LAMP1:NPTX2 ratio serves as a transdiagnostic biomarker correlate of psychotic experience, cognitive impairment, and functional disability. The claim is supported by convergent, cross-validated feature selection showing LAMP1 and NPTX2 as top predictors across psychotic experience, cognitive impairment, and functional impairment, plus independent associations of the LAMP1:NPTX2 ratio with global functioning and multiple cognitive domains. While they were not able to validate this ratio in the second cohort, the evidence seems strong that the LAMP1:NPTX2 ratio is valuable and will be a focus of further validation and broader diagnostic assessment for cognition.
3) CSF synaptic protein levels show within-individual stability over time in healthy controls. The authors use this "non-disease covariance adjustment" to enhance detection of psychiatric disorder-related changes. It builds a composite (SYUB, STX7, NCAM2) that captures most shared variance, showing that adjustment enhances biological signal, including stronger brain eQTL associations and a first-episode psychosis NPTX2 deficit that becomes significant only after normalization to SYUB. Did the authors test whether conclusions are robust to alternative reference sets or normalization strategies? Should this be a more widely used reference or is it specific to this data set?
4) Figure 4B presents group-to-control contrasts and indicates that the effect is mainly driven by individuals later diagnosed with schizophrenia, but it does not report a direct within–first-episode comparison between schizophrenia and non-schizophrenia outcomes. Was this evaluated?
The authors may want to reference prior work implicating deficits of parvalbumin interneuron (PV-IN) function in neuropsychiatric disease-especially schizophrenia. The authors' data support this model since NPTX2 functions primarily at the excitatory synapse onto PV-IN where GRIA4 is selectively expressed. This would enhance the mechanistic interpretation of their very nice data.
Paul Worley

Reviewer #3

(Remarks to the Author)
Reviewer Comments

This manuscript addresses an important and understudied topic by investigating CSF biomarkers related to synaptic dysfunction across major psychiatric disorders. The study is unique in its robust methodology and unusually large CSF sample, which is difficult to obtain. The work is highly relevant and has strong potential impact. However, several issues limit interpretability, particularly regarding the rationale for certain analyses, clarity of data presentation, and overall structure of the results. The manuscript would benefit from additional analyses, improved clarity, and more detailed methodological explanations. Specific comments are listed below:

Rationale for focusing exclusively on synaptic proteins
It is unclear why the authors restricted the analysis to synaptic proteins. Given the comprehensive CSF proteomic dataset, this appears to be a missed opportunity to perform an unbiased assessment, especially since multiple biological mechanisms may contribute to these disorders.

Ambiguous statement in the results
The sentence "Using a data-driven approach to model transdiagnostic contrasts—psychotic experience, cognitive and functional impairment—we identified a biomarker correlate: elevated LAMP1… and reduced NPTX2…" is unclear and should be rephrased for precision and readability.

Interpretation of the replication cohort
Although the use of a replication cohort is commendable, it does not include the same diagnostic spectrum as the discovery cohort. It appears the authors may have identified not disorder-specific markers, but rather markers associated with a particular symptom dimension shared across disorders. If so, this should be clearly highlighted and the clinical utility of such symptom-related markers—especially given the invasiveness of CSF—should be discussed in more depth.

Group differences and potential confounders
The sample size is impressive, but there seem to be important differences across clinical groups (e.g., illness duration, lifetime psychosis, substance use, medication). Have these factors been controlled for, given their substantial impact on CSF proteomics? These variables should be clearly reported and discussed in the demographics section. The description of the control group also requires clarification.

Placement and definition of synapse panels
The description of the CSF synaptic protein panels is largely methodological and should be moved from the results to the methods section. Additionally, it is questionable whether CTSD and LAMP1 can be classified as synaptic proteins, as they

are primarily lysosomal markers.

Normalization strategy in controls
The control-only analysis is interesting but complex. It is unclear which reference marker or composite score was ultimately used, and whether subsequent analyses were normalized to this score. The numerous transformations may complicate clinical translation. Why not adopt a simpler approach, such as using ratios to a reference protein (commonly used in AD biomarker studies)?

Definition of cognitive impairment
The operational definition of cognitive impairment is unusual. Has it been validated?

PRS methodology
The reference or methodological details for polygenic risk score (PRS) computation are missing.

Unadjusted vs. normalized CSF levels
The authors state they report unadjusted CSF levels. Are these raw concentrations without normalization? Were comparisons corrected for multiple testing? A figure analogous to Figure 1 should be provided for unadjusted values in the Supplementary Material. Additionally, a volcano plot may improve interpretability.

Incorrect figure reference
In line 182, the figure reference does not correspond to the text.

Clarity of case–control findings
The main findings from the case–control comparisons are difficult to interpret due to the plotting choices. It is unclear which proteins show cross-diagnostic vs. disorder-specific effects. For example, the manuscript states that NPTX2 and NPTXR mark SCZ+, yet later they are presented as pleiotropic markers. Both text and figures need to be revised to clearly present:
(i) which proteins differ across pair-wise comparisons,
(ii) which are disorder-specific, and
(iii) which are shared across diagnoses.
Consider alternative plotting formats (e.g., volcano plots, forest plots, Venn diagrams).

Value of markers significant only in unadjusted analyses
I do not agree that markers significant only in unadjusted analyses are necessarily of lesser interest. Independent validation in comparable external cohorts is needed.

Diagnostic performance and potential for multivariate models
Although some single-marker AUCs reach significance, the overall performance is limited, and top markers are shared across disorders. It would be worthwhile to evaluate multivariate models earlier in the analysis, as later done for symptom dimensions. However, a power analysis should first be performed. In this context, adjusted values may not be required, as multi-marker combinations can reduce intra-individual variability.

Complement factors and NFL results
Complement and NFL findings should be plotted directly in the relevant figure. It is unclear why only the correlation between NPTX2 and C1q is shown, particularly since it is negative.

Multivariate models for symptom associations
The multivariate associations with symptom domains show wide confidence intervals, suggesting substantial heterogeneity and/or limited sample size. Are these results reliable? Could controls and non-psychiatric cases be pooled? Were these models run with adjusted or unadjusted values? This point is important because a ratio of two proteins—likely better normalizing inter-individual variability—is used later. If ratios improve performance (as shown in Fig. 4 AUCs), they should be introduced earlier in the case–control analyses.

Clarification of Figure 3d
The interpretation of Fig. 3d is not clear and needs clarification.

Cognitive performance analyses
Please include direct references to the corresponding figures. The same issues of low power and high heterogeneity (visible in wide AUC ranges) are present in both the cognitive and functional impairment analyses. How were groups defined? Are the reported R^2 values correct?

Figure 4 AUC values
Figure 4 should include the explicit AUC values. Are the AUC improvements after adding PRS statistically significant?

Replication cohort and validation
How comparable are the FEP cases to the validation cohort? This seems more like an additional analysis rather than a true validation. A proper validation in an independent cohort with similar clinical characteristics is needed. Additionally, it is unclear which specific outcome the authors intended to validate. Please verify the last panel in Figure 4.

Version 1:

Reviewer comments:

Reviewer #1

(Remarks to the Author)
Authors have addressed the major comments by toning down the claims. However, some minor issues remain:
• Fig. 1F is called before Figs 1C-E.
• Line 227: “However, NfL concentrations correlated negatively with NPTX2 and NPTXR regressed on covnd (R = -0.32 and -0.40, respectively; Figure 2C). Fig. 2C only shows NPTX2.
• Fig 2D needs statistical analysis.

Reviewer #2

(Remarks to the Author)
The authors effectively addressed my limited questions.

Reviewer #3

(Remarks to the Author)
The authors have answer most of my concerns. There are still some limitations inherent to the limited sample size and the difficulty of obtaining such cohorts. But considerijg the lack of such type of studies in the pychiatry field, and the importance of it, I think the manuscript can be accepted for publication.

Open Access This Peer Review File is licensed under a Creative Commons Attribution 4.0 International License, which permits use, sharing, adaptation, distribution and reproduction in any medium or format, as long as you give appropriate credit to the original author(s) and the source, provide a link to the Creative Commons license, and indicate if changes were made.
In cases where reviewers are anonymous, credit should be given to 'Anonymous Referee' and the source.
The images or other third party material in this Peer Review File are included in the article’s Creative Commons license, unless indicated otherwise in a credit line to the material. If material is not included in the article’s Creative Commons license and your intended use is not permitted by statutory regulation or exceeds the permitted use, you will need to obtain permission directly from the copyright holder.
To view a copy of this license, visit https://creativecommons.org/licenses/by/4.0/

Reviewer #1 (Remarks to the Author)

This study by Göteson et al. used targeted mass spectrometry to measure low-abundance synaptic proteins in CSF from 672 individuals across major psychiatric disorders and controls, revealing most severe changes in schizophrenia, with intermediate ones in bipolar disorder and ADHD. By modeling transdiagnostic clinical features, the authors identified the LAMP1:NPTX2 ratio as a biomarker associated with psychotic experience, cognitive impairment, and functional decline. Findings were externally replicated in first-episode psychosis, supporting early and measurable synaptic pathology across psychiatric conditions. The study utilizes a large cohort, applies sophisticated methods, and supports the concept of synaptic dysfunction as a shared biological substrate across major psychiatric disorders, particularly in schizophrenia and bipolar disorder.

Thank you for your careful review and insightful comments. Please find our responses below.

However, there are some concerns:

R1:1 Uneven diagnostic sampling: Large BD cohorts vs smaller ADHD, AN, and SCZ groups may bias effect estimation. AN cohort is 100% females. Moreover, only a few SCZ participants completed neurocognitive assessments, limiting transdiagnostic inference.

Thank you for raising this important limitation. The diagnostic group sizes are uneven, which we also acknowledge in the manuscript. This imbalance was not by design but reflects the availability of biobanked CSF samples across the contributing cohorts. Because CSF material from psychiatric populations is scarce, we analyzed all available material.

An alternative strategy would be to downsample the BD group to match the smaller diagnostic groups. However, this would reduce statistical power and would not necessarily improve generalizability. Instead, we present standardized effect sizes (β) for each diagnostic group relative to the same control sample, which allows comparisons across disorders despite differences in groups size. While statistical power differs between groups, effect size estimates remain more comparable. In Figure 2, we show both formally significant associations (1% FDR, marked with an asterisk) and suggestive associations (< 20% FDR) to illustrate general trends across disorders.

In the revised main text, however, only formally significant findings are interpreted. This approach avoids overinterpreting the absence of statistical significance in smaller groups, which may reflect limited statistical power rather than a true absence of biological differences. We have also made minor edits to Figure 2 to clarify the meaning of the asterisk.

For the dimensional analyses, all 505 cases were included irrespective of diagnosis. These models therefore do not depend on balanced diagnostic group sizes. Most SCZ+ participants lacked complete neurocognitive assessments, which represents a limitation. However, because few SCZ+ cases contributed cognitive data, it is unlikely that the dimensional findings are driven primarily by a SCZ-like phenotype. Instead, they likely reflect transdiagnostic patterns present across the included disorders.

The following changes have been made to the main text (altered wordings in *italics*):

- Limitations (page 16): "Diagnostic group sizes were uneven, with a majority of cases in the BD spectrum. *Further, cases with AN and SCZ+ were missing certain data modalities.* However, the broad symptom profile of BD may render it a useful proxy for psychiatric illness more generally. The AN group was small (n=40), younger than controls, and compared only to female controls. Strict multiple-testing correction may have reduced power in this subgroup, as several associations were notable (e.g., higher SYT1, lower NLGNX) but did not reach the strict 1% false discovery rate threshold. All case-control results are reported in the Supplement."

R1:2 Case-control comparisons of CSF synapse proteins across disorders: Several proteins were significantly associated with disease but the AUCs ranged from 0.69-0.78, potentially limiting their utility as diagnostic biomarkers. The authors state that there were no significant associations with in BD2+ or AN (Line 172), but Figure 2A shows several significant proteins for BD2+ (e.g. NPTX2). Also, it is unclear if Figure 2A, which is not referred to in the text, includes adjustment for non-disease covariance or not.

Thank you for raising this point concerning case–control discrimination of the biomarkers. We agree that the observed AUC values (~0.7–0.8) are modest. Given the biological heterogeneity of psychiatric disorders as defined by DSM/ICD criteria, we did not expect single CSF biomarkers to achieve very high diagnostic accuracy. Rather, our aim was to identify biologically informative signals that may contribute to future biomarker-based stratification approaches.

For context, biomarker performance in other fields has improved significantly as disease definitions have shifted from clinical syndromes to biologically defined entities (e.g., Alzheimer's disease). In psychiatric disorders, such biological reference standards are not yet established, which likely limits achievable diagnostic performance at this stage. In this context, the observed AUC values should not be viewed as discouraging but rather as an early step in biomarker development.

As for Figure 2A, we kindly refer the reviewer to R1:1, which clarifies the distinction between suggestive and formally significant associations. Synaptic protein concentrations were adjusted for non-disease covariance in the figure, as indicated in the legend. We apologize for not explicitly referencing Figure 2A in the previous version of the manuscript. This has now been corrected, and the figure is clearly cited in the revised main text.

R1:3 Complement components and neurodegeneration biomarkers as correlates of synaptic dysfunction: "Compared with controls, CSF NfL concentrations were significantly higher in SCZ+ (β [95%CI]=0.24 [0.01, 0.48], P=0.042), BD1 (0.25 [0.10, 0.40], P=0.001), BD2+ (0.19 [0.06, 0.32], P=0.004), and AN (0.25 [0.02, 0.48], P=0.03)." – these results seem inconsistent with Figure 2A.

Thank you for noting this inconsistency. Issues related to Figure 2A are addressed in R1:1, but we agree that the reporting of statistical significance for NfL was inconsistent in the original submission. In the previous version, nominal significance ($P < 0.05$) was used when describing NfL results, whereas synaptic and complement proteins were interpreted using the 1% FDR threshold. We have now revised the manuscript to apply the same 1% FDR threshold to define formal statistical significance throughout the Results section. For transparency, we still report NfL effect size estimates and nominal *P*-values for all case groups, while clearly indicating which findings remain significant after multiple-testing correction. The main text and figures are now consistent in this regard.

In the initial submission, we included post hoc analyses within the SCZ+ group suggesting that higher NfL values were primarily driven by individuals with schizophrenia rather than other psychotic disorders. Because the overall SCZ+ group result was not significant after multiple-testing correction, these post hoc analyses have been removed.

The following changes have been made to the main text (altered wording in *italics*):

- Results (page 9): *"Compared with controls, CSF NfL concentrations were higher in BD1 (0.25 [0.10, 0.40], P=0.001), with similar effect size estimates in SCZ+ (β [95% CI]=0.24 [0.01, 0.48], P=0.042), BD2+ (0.19 [0.06, 0.32], P=0.004), and AN (0.25 [0.02, 0.48], P=0.03), but only BD1 remained statistically significant after multiple-testing correction. Cases with ADHD did not differ from controls (0.15 [-0.02, 0.31], P=0.078)."*

R1:4 CSF biomarker ratio reflecting psychotic liability, cognitive, and functional impairment: Although they were significant, the correlations of LAMP1:NPTX2 ratio with cognitive scores were low (all R^2 < 0.1). Authors state that LAMP1:NPTX2 ratio showed a progressive increase along the ADHD-BD-SCZ spectrum (Figure 3C), but there is no statistical analysis and Figure 3C is not cited in the text.

Thank you for this comment. We agree that the correlations between the LAMP1:NPTX2 ratio and cognitive scores are modest and should not be interpreted as explaining a large proportion of cognitive variance. The reported R^2 values represent the variance explained by the biomarker ratio beyond age and sex. Inspection of the scatter plots (Figure 3G) suggests that the association is primarily driven by individuals with marked cognitive impairment, rather than reflecting a linear association across the full range of cognitive performance, as noted in the main text. The relatively low R^2 values are therefore expected given the heterogeneity of cognitive performance in this cohort.

In response to related comments (see R3:17), we refined the models by estimating age and sex effects in controls only and residualizing the biomarker ratio and cognitive scores accordingly. This led to only minimal changes in R^2 values and did not alter the overall interpretation.

We also appreciate the suggestion to statistically evaluate the apparent increase in LAMP1:NPTX2 across diagnostic groups. We have now formally tested this trend and report the result in the revised manuscript. For consistency, a similar analysis was done for global assessment of functioning (GAF-F).

We apologize for not explicitly referencing Figure 3C in the previous version. This has now been corrected, and the figure is cited in the Results section.

The following changes have been made to the main text (altered wording in *italics*):

- Results (page 10): "In the full cohort, this ratio showed a progressive increase along the ADHD-BD-SCZ spectrum, with and without psychotic experience (*P for trend<0.05*; Figure 3C), suggesting relevance to psychotic liability that extend beyond categorical diagnosis."
- Methods (page 21): "Tests for trend across ordered groups were performed using linear regression models."

R1:5 Although the authors conclude that the LAMP1:NPTX2 ratio reflects "synaptic dysfunction as a transdiagnostic feature," this interpretation is limited by missing data in key diagnostic groups ("Participants with AN were excluded due to missing clinical data, and most individuals with SCZ+ had not undergone cognitive testing").

Thank you for this important remark. We agree that the term "transdiagnostic" could be interpreted too broadly, given that participants with AN were excluded from the dimensional models and that most individuals with SCZ+ did not complete cognitive testing. Our intention was not to imply that the LAMP1:NPTX2 ratio generalizes across all psychiatric disorders, but rather that it reflects dimensions of psychotic liability, cognitive impairment, and chronic functional deficits that cut across diagnostic categories represented in this cohort.

We have revised the manuscript to clarify this point and now use more precise wording, emphasizing that the findings apply to certain psychiatric disorders and dimensional constructs within this dataset.

The following changes have been made to the main text (altered wordings in *italics*):

- Abstract (page 2): "These results support synaptic pathology as a measurable and transdiagnostic feature *across several psychiatric disorders* and highlight the potential of integrating fluid and genetic biomarkers"

- Results (page 11): “Together, these results suggest a shared underlying biology across psychotic liability, cognitive deficits, and functional impairment, implicating synaptic dysfunction ~~as a transdiagnostic feature~~”.
- Discussion (page 15): “These findings indicate that synaptic dysfunction represents a measurable and clinically relevant pathology in *several* psychiatric disorders.”
- Limitations (page 16): “*Cases with AN and SCZ+ were missing certain data modalities.”*
- Conclusion (page 17): “This study shows that synaptic dysfunction is detectable in CSF across *several* major psychiatric disorders and relates to clinically relevant outcomes.”

R1:6 Medication confounding: Though variance explained was small (~1%), most participants were medicated at CSF draw, especially in BD and SCZ.

Thank you for raising this important point. Most cases were receiving psychotropic medication at the time of CSF sampling, which is an inherent limitation in naturalistic clinical cohorts. Because treatment is closely tied to symptom severity and diagnosis, it is difficult to disentangle medication effects from disease-related biology. As no controls were exposed to medication, case–control analyses cannot be directly adjusted for drug exposure.

To evaluate potential medication effects, we estimated the variance in protein concentrations attributable to medication exposure using linear regression models adjusting for age, sex, diagnostic group, and cov_{nd}. Because the 1% threshold may appear arbitrary, we refined these analyses by adding an inference test to evaluate whether including medication improved model fit. In doing so, we also corrected a minor data processing error that led to negative R^2 values in a small number of associations, and grouped first- and second-generation antipsychotics to obtain a more robust estimate, as first-generation antipsychotics were relatively rare (n=16 exposed). Analyses were corrected for multiple comparisons using the Benjamini–Hochberg false discovery rate.

The overall interpretation remained unchanged. Among the top biomarker candidates, only GRIA4 showed a modest association with antipsychotic exposure (R^2=0.018), suggesting that medication is unlikely to drive the main findings. The most prominent medication-related effects involved lithium and presynaptic vesicle cycling proteins (e.g., CPLX1, CPLX2), but these were not central to the primary case–control results.

Notably, a key finding (reduced NPTX2) was also observed in the independent first-episode psychosis cohort, where participants were sampled at illness onset and therefore had limited cumulative exposure to psychotropic medication.

While these analyses are informative, they cannot fully account for medication as a confound. We therefore interpret the present results as arising from naturalistic clinical samples and acknowledge medication exposure as an important limitation. Biomarker validation in future cohorts—including drug-naïve populations—will be necessary to fully evaluate the clinical utility of these findings.

The following changes have been made to the main text (altered wordings in italics):

- Results (pages 12–13): "*To evaluate the effect of medication on case–control–associated proteins*, we used linear regression models to estimate the proportion of variance in protein concentrations attributable to medication (Table S4). Among the top-performing biomarkers, only GRIA4 showed a modest association with *antipsychotics (R^2=0.018)*, suggesting that medication is unlikely to be a major driver of the observed case–control differences. *The most prominent medication-related effects involved lithium and presynaptic vesicle cycling proteins (e.g., CPLX1, CPLX2), which were not central to the primary case–control results.*"
- Limitations (page 16): "*This study represents an early stage of biomarker discovery. The data arise from naturalistic clinical cohorts, where factors such as medication exposure and other clinical characteristics cannot be fully disentangled from disease-related biology. Independent validation in external cohorts—and potentially translation to less invasive modalities—will therefore be necessary before clinical application can be considered*."
- Table S4 was updated accordingly

R1:7 LAMP1 was not available in the replication cohort, which limited replication.
Thank you for pointing out this limitation. LAMP1 was not available in the external FEP cohort, which restricted direct replication of the LAMP1:NPTX2 ratio. However, the main signal in the ratio appears to be driven by reduced NPTX2. In the multivariate models, LAMP1 and NPTX2 contributed with similar but opposing weights, while LAMP1 alone was not associated with case–control status in the primary analyses.

Consistent with this interpretation, expressing NPTX2 relative to other stable proteins (e.g., SYUB) produced a similar signal as LAMP1:NPTX2 in the main cohort (Figure 3E). We therefore interpret the external analysis primarily as an extension of the NPTX2 finding rather than a full validation of the LAMP1:NPTX2 ratio. This clarification has been added to the Discussion.

The following changes have been made to the main text (altered wordings in *italics*):

- Discussion (page 16): "Notably, while LAMP1 was not associated with SCZ+ or BD in our data (only a moderate decrease in ADHD), the LAMP1:NPTX2 ratio improved case-control stratification relative to NPTX2 alone. *A similar improvement with SYUB:NPTX2 suggests that LAMP1 mainly contributes through shared covariance rather than mechanism-specific effects*".

R1:8 Figures are incorrectly cited or not cited, making it difficult to follow the manuscript, e.g.:
o Figure 1A, 1E are not referred to in the text, and 1D is cited after 1F.
o Line 126 R^2 range: 35–79% doesn't match Figure 1C.
o Line 182, 237: incorrect figure citations.

Thank you for pointing to these mistakes, which have now been corrected.

Reviewer #2 (Remarks to the Author)

This study by Göteson et al demonstrates that synaptic dysfunction is a measurable and transdiagnostic feature of major psychiatric disorders. The paper presents a comprehensive analysis by quantifying 37 synaptic proteins using mass spectrometry across large and clinically diverse cohorts with schizophrenia, bipolar disorder, ADHD, and anorexia nervosa. This broad investigation of a large protein panel across multiple distinct psychiatric conditions represents a significant advance in the search for transdiagnostic biomarkers. The paper identifies a novel ratio of two cerebrospinal fluid proteins, LAMP1 and NPTX2, as a robust marker. This ratio correlates with psychotic experiences, cognitive deficits, and functional impairment across different diagnostic categories. While NPTX2 and LAMP1 have been studied in other neurological contexts, their specific ratio as a proxy for synaptic pathology in major psychiatric disorders is a new finding.

Thank you for your encouraging summary and careful review. Please find our responses below.

The paper makes several interesting claims that appear well-founded.

R2:1 Synaptic protein concentrations are markedly reduced in schizophrenia spectrum disorders, with intermediate reductions in bipolar disorder and ADHD. Schizophrenia spectrum shows consistent, sizable reductions across many synaptic proteins (notably NPTX2 and GRIA4), while bipolar disorder and ADHD exhibit fewer and smaller changes, consistent with an 'intermediate' pattern. Did the authors test if NPTX2 and GRIA4 are correlated in CSF?

Yes, we evaluated this correlation, which is illustrated in Figure 2B. The Pearson correlation coefficient was r=0.78 for raw CSF abundances and r=0.48 after adjustment for non-disease covariance (cov_{nd}). These results suggest that the markers share some covariance but also capture partially distinct biological signals, consistent with their different association patterns across diagnostic groups (Figure 2A).

R2:2 The LAMP1:NPTX2 ratio serves as a transdiagnostic biomarker correlate of psychotic experience, cognitive impairment, and functional disability. The claim is supported by convergent, cross-validated feature selection showing LAMP1 and NPTX2 as top predictors across psychotic experience, cognitive impairment, and functional impairment, plus independent associations of the LAMP1:NPTX2 ratio with global functioning and multiple cognitive domains. While they were not able to validate this ratio in the second cohort, the evidence seems strong that the LAMP1:NPTX2 ratio is valuable and will be a focus of further validation and broader diagnostic assessment for cognition.

Thank you for this positive summary of our findings. We agree with the reviewer that the LAMP1:NPTX2 ratio represents a promising candidate biomarker and should be the focus of further validation and broader diagnostic assessment across psychiatric disorders and related cognitive traits.

R2:3 CSF synaptic protein levels show within-individual stability over time in healthy controls. The authors use this "non-disease covariance adjustment" to enhance detection of psychiatric disorder-related changes. It builds a composite (SYUB, STX7, NCAM2) that captures most shared variance, showing that adjustment enhances biological signal, including stronger brain eQTL associations and a first-episode psychosis NPTX2 deficit that becomes significant only after normalization to SYUB. Did the authors test whether conclusions are robust to alternative reference sets or normalization strategies? Should this be a more widely used reference or is it specific to this data set?

Thank you for raising this point. Several reviewers commented on this topic, and we have clarified and slightly reframed the presentation in the revised manuscript. However, the key principles remain unchanged.

Our dataset was not designed to define a broadly applicable CSF reference standard. While others have attempted this using large-scale unbiased proteomic datasets (e.g., Karlsson et al., Nat Com 2024), there is currently no consensus reference strategy in the field. In untargeted proteome-wide datasets, global metrics such as the overall protein mean or PC1 are increasingly used, but this approach is not directly transferable to targeted panels.

Here, we defined theoretical characteristics of an "ideal" reference protein and systematically ranked the measured synaptic proteins against these criteria. The three highest-ranked proteins were combined into a composite score to approximate non-disease covariance (now referred to as cov_{nd}; see R3:6). A composite was chosen to reduce dependence on a single assay. The ranking did not yield a strict cutoff, and we evaluated alternative setups including up to five top-ranked proteins. These alternative composites were highly correlated (all $r > 0.99$) and did not materially change the results, indicating that conclusions were robust to reasonable variation in reference composition.

The cov_{nd} reference is therefore constructed for this dataset and should not be interpreted as a universally applicable standard. Establishing generalizable CSF reference candidates across brain disorders remains an open challenge. Such work will likely need to bridge data modalities — including longitudinal CSF, genomics, and post-mortem cortices — across a wide array of brain disorders.

A central contribution of this study is the demonstration that synaptic protein levels are largely stable within individuals over time in relatively young healthy controls. The individual factor was the dominant source of variability, by far exceeding the contribution of assay variability, age, sex, and timepoint. This pattern supports the rationale for accounting for non-disease covariance in case-control studies, particularly when effect sizes are expected to be modest — such as in early stages of neurodegeneration or in psychiatric disorders.

In response to this and related comments, we have clarified the relevant sections of the main text. The most relevant change with respect to this question is found the Discussion (detailed below). Please also refer to R3:6 and R3:12 for other related changes.

- Discussion (page 16): "*Longitudinal analyses indicate that individually determined covariance is a stable and dominant source of variability in CSF synaptic proteins in the absence of disease and advanced age*. Accounting for such non-disease covariance may enhance signal-to-noise in CSF biomarker studies. Notably, in our cohort sampled at illness onset—a critical window for biomarker-based diagnostics—NPTX2 reductions were only detectable after such adjustment, with unadjusted models narrowly missing statistical significance. *However, the specific reference constructed in this study (cov_{nd}) was not intended for general use. Further work will be needed to define broadly applicable reference strategies*.

R2:4 Figure 4B presents group-to-control contrasts and indicates that the effect is mainly driven by individuals later diagnosed with schizophrenia, but it does not report a direct within–first-episode comparison between schizophrenia and non-schizophrenia outcomes. Was this evaluated?

The requested comparison had not been performed previously but is clinically relevant. We have now conducted this analysis, which is included in the revised manuscript. There was no statistically significant difference between FEP-SCZ and FEP-nSCZ: β (95% CI) = -0.27 (-0.79–0.25); *P* = 0.30.

The following changes have been made in the main text (altered wording in *italics*):

- Results (page 14): "This effect *seemed* primarily driven by individuals later diagnosed with schizophrenia (FEP-SCZ; β [95% CI] *vs controls* = -0.56 [-0.97, -0.14], *P* < 0.01), rather than those with non-schizophrenia outcomes (FEP-nSCZ; β [95% CI] *vs controls* = -0.25 [-0.73, 0.24], *P* = 0.31; Figure 4B), *although FEP-SCZ and FEP-nSCZ did not differ statistically (FEP-SCZ vs FEP-nSCZ: β [95% CI] = -0.27 [-0.79–0.25]; P = 0.30).*"

R2:5 The authors may want to reference prior work implicating deficits of parvalbumin interneuron (PV-IN) function in neuropsychiatric disease-especially schizophrenia. The authors' data support this model since NPTX2 functions primarily at the excitatory synapse onto PV-IN where GRIA4 is selectively expressed. This would enhance the mechanistic interpretation of their very nice data.

Thank you for this helpful suggestion. We agree that the role of parvalbumin interneurons is highly relevant to the interpretation of our findings. NPTX2 has been shown to regulate excitatory synapse maturation onto these interneurons, where AMPA receptors containing GRIA4 are expressed. We have now added a short paragraph in the Discussion linking the observed NPTX2 and GRIA4 alterations to models of excitation–inhibition imbalance in psychiatric disorders. We also updated the 'postsynaptic' category label in Figure 2 to 'postsynaptic specialization'.

The following changes have been made to the main text (altered wordings in *italics*):

- Discussion (page 15): "*Specifically, NPTX2 interacts with NPTXR and GRIA4 on parvalbumin-expressing interneurons to regulate excitation-inhibition balance,*

which is critical for cognitive processes and potentially disrupted in certain neuropsychiatric disorders."

Paul Worley

Reviewer #3 (Remarks to the Author)

This manuscript addresses an important and understudied topic by investigating CSF biomarkers related to synaptic dysfunction across major psychiatric disorders. The study is unique in its robust methodology and unusually large CSF sample, which is difficult to obtain. The work is highly relevant and has strong potential impact. However, several issues limit interpretability, particularly regarding the rationale for certain analyses, clarity of data presentation, and overall structure of the results. The manuscript would benefit from additional analyses, improved clarity, and more detailed methodological explanations. Specific comments are listed below:

Thank you for your constructive and detailed review. Please find our responses below.

R3:1 Rationale for focusing exclusively on synaptic proteins
It is unclear why the authors restricted the analysis to synaptic proteins. Given the comprehensive CSF proteomic dataset, this appears to be a missed opportunity to perform an unbiased assessment, especially since multiple biological mechanisms may contribute to these disorders.

Thank you for this comment. We agree that multiple biological mechanisms likely contribute to these disorders, and that only some of these may be captured by the biomarkers studied here. Our focus on synaptic proteins was motivated by the substantial body of evidence implicating synaptic dysfunction in the pathophysiology of major psychiatric disorders, as summarized in the Introduction.

We would like to clarify that the experimental approach in this study did not generate an unbiased proteomic dataset from which synaptic targets were subsequently selected. Rather, the study used a predefined targeted LC-MS/MS panel designed to quantify a set of low-abundant synaptic proteins. Targeted mass spectrometry panels offer advantages in sensitivity and quantification for specific low-abundant proteins compared with discovery-based proteomic methods.

Broader, unbiased CSF proteomic profiling could certainly provide information on other biological pathways, but such analyses were outside the scope of the present study.

R3:2 Ambiguous statement in the results
The sentence "Using a data-driven approach to model transdiagnostic contrasts—psychotic experience, cognitive and functional impairment—we identified a biomarker correlate: elevated LAMP1… and reduced NPTX2…" is unclear and should be rephrased for precision and readability.

Thank you for pointing out this ambiguity. We agree that the phrasing could be clearer and have revised the sentence accordingly.

The following relevant change has been made to the main text (altered wording in *italics*):

- Abstract (page 2): "Using a data-driven approach to model transdiagnostic contrasts—psychotic experience, cognitive and functional impairment—*a shared two-biomarker signature emerged*: elevated LAMP1 …"

R3:3 Interpretation of the replication cohort
Although the use of a replication cohort is commendable, it does not include the same diagnostic spectrum as the discovery cohort. It appears the authors may have identified not disorder-specific markers, but rather markers associated with a particular symptom dimension shared across disorders. If so, this should be clearly highlighted and the clinical utility of such symptom-related markers—especially given the invasiveness of CSF—should be discussed in more depth.

We acknowledge that the external cohort does not represent a direct replication cohort but rather an extension of the main findings, although it includes both new cases and new controls. We have revised the main text to reflect this distinction more clearly.

The following changes have been made to the main text (altered wordings in *italics*):

- Abstract (page 2): "Key findings were *extended to* an independent cohort at first-episode psychosis"
- Introduction (page 4): "Key findings were *extended to* an independent cohort of individuals with first–episode psychosis (FEP) and controls"
- Results (page 13, section heading): "Synaptic dysfunction is detectable at first-episode psychosis"
- Results (13): "Finally, to evaluate the relevance of our findings at *psychotic illness* onset, …"
- Conclusions (page 17): "Key results were *extended to* FEP and corroborate prior evidence that"

We also agree with the reviewer that the biomarkers do not appear to be disorder-specific, as discussed in the initial paragraphs of the Discussion. This is not unexpected, as CSF biomarkers may reflect biological processes that cut across clinical syndromes as defined by DSM or ICD criteria. Further work is needed to clarify the clinical significance of the findings. At present, we view the results as a promising step toward biologically informed stratification in psychiatry, but one that requires further validation. We also acknowledge the invasiveness of CSF sampling as limitation in the manuscript.

The following changes have been made to the main text (altered wordings in *italics*):

- Discussion (page 15): "*The biomarker associations cut across diagnostic categories and appear to reflect underlying symptom dimensions rather than*

diagnostic specificity. Some proteins nevertheless showed stronger signals in certain groups; for example, GRIA4 was most clearly reduced in SCZ+, whereas SYT1 was mainly elevated in ADHD. Whether such patterns reflect clinically meaningful subdimensions remains to be determined."

- Limitations (page 16): "*This study represents an early stage of biomarker discovery, with limitations to consider. The data arise from naturalistic clinical cohorts, where factors such as medication exposure and other clinical characteristics cannot be fully disentangled from disease-related biology. Independent validation in external cohorts—and potentially translation to less invasive modalities—will therefore be necessary before clinical application can be considered.*"

R3:4 Group differences and potential confounders
The sample size is impressive, but there seem to be important differences across clinical groups (e.g., illness duration, lifetime psychosis, substance use, medication). Have these factors been controlled for, given their substantial impact on CSF proteomics? These variables should be clearly reported and discussed in the demographics section. The description of the control group also requires clarification.

Thank you for this important comment. The cohort reflects naturalistic clinical populations and is therefore heterogeneous with respect to illness duration, lifetime psychosis, substance use, and medication exposure. These variables were not included as covariates in the primary models because many are closely linked to the underlying illness and treatment decisions, making them difficult to interpret as independent confounders.

Evidence on how these factors influence CSF proteomics in psychiatric disorders remains limited. Moreover, controlling for some of these variables would require specific assumptions about disease biology. For example, adjusting for illness duration would implicitly assume a progressive molecular disease process, which has not been established for these psychiatric conditions. Comorbid substance use disorders were also relatively uncommon in the cohort (< 10–20%), making it unlikely that they materially influence the main findings. With respect to medication, this remains a limitation of our study design and is discussed in more detail in R1:6.

Details of the control group are provided in the referenced cohort profile and summarized in the Methods section of the manuscript. We have added a reference to the published cohort profile in the relevant section.

To address the reviewer's concern, we have clarified the limitations related to naturalistic cohorts and clinical heterogeneity in the manuscript.

The following changes have been made to the main text (altered wordings in *italics*):

- Limitations (page 16): "*This study represents an early stage of biomarker discovery. The data arise from naturalistic clinical cohorts, where factors such as medication exposure and other clinical characteristics cannot be fully disentangled from disease-related biology. Independent validation in external*

cohorts—and potentially translation to less invasive modalities—will therefore be necessary before clinical application can be considered."

R3:5 Placement and definition of synapse panels
The description of the CSF synaptic protein panels is largely methodological and should be moved from the results to the methods section. Additionally, it is questionable whether CTSD and LAMP1 can be classified as synaptic proteins, as they are primarily lysosomal markers.

Thank you for this suggestion. We agree that describing the synaptic protein panels could logically be placed in the Methods section. However, as the journal format places the Methods section after the Discussion, we chose to briefly introduce the studied proteins in the Results to aid readability before presenting the findings. We have therefore retained but would be happy to revise it if the editor prefers otherwise.

Regarding CTSD and LAMP1, we acknowledge that these proteins are not exclusively synaptic. However, both proteins are indexed in SynGO, an expert-curated consensus ontology of synaptic genes and proteins and were therefore included within the broader synaptic framework used in this study. This last part was clarified in the main text (altered wordings in *italics*): "…, and *synapse-expressed* endolysosomal *proteins* (CTSD, LAMP1)".

R3:6 Normalization strategy in controls
The control-only analysis is interesting but complex. It is unclear which reference marker or composite score was ultimately used, and whether subsequent analyses were normalized to this score. The numerous transformations may complicate clinical translation. Why not adopt a simpler approach, such as using ratios to a reference protein (commonly used in AD biomarker studies)?

Thank you for this comment. We agree that this part of the analysis may seem complex, and we appreciate the opportunity to clarify the rationale and presentation.

First, regarding complexity. We acknowledge that the original presentation was dense and have revised the main text to simplify the language, with additional technical details moved to the Supplementary Material. In the revised version, we emphasize the key observation underlying this approach: that variability in CSF synaptic protein concentrations is largely determined by stable, individual-specific covariance in the absence of disease and advanced age.

Second, regarding the reference used. A single composite reference was used in the main cohort analyses. We now refer to this consistently as cov_{nd}, and the manuscript has been revised to define and apply this term clearly throughout. The cov_{nd} reference was derived from baseline (T1) concentrations of three proteins (SYUB, STX7, NCAM2) using a composite score, with the aim of approximating this broader covariance structure. This reference explained a substantial proportion of variance in synaptic protein concentrations, accounting for 58% at T1 and 49% at T2, sampled 6.5 years later. These results indicate that a large component of CSF synaptic protein variability is stable over time, although the physiological mechanisms underlying this covariance remain to be determined.

In the external FEP cohort, only a limited subset of tryptic peptides was available and the full synaptic panel was not measured. Therefore, cov_{nd} could not be constructed in that dataset. Instead, SYUB—one component of cov_{nd} and unaltered between cases and controls—was used as a proxy reference for non-disease covariance.

Third, regarding clinical translation and the use of simple ratios. We agree that biomarker ratios are intuitive and widely used in other fields, such as Alzheimer's disease research. However, those applications typically rely on established disease biology and validated "housekeeping" markers. In psychiatric disorders, no such ground truth currently exists. Introducing all possible pairwise ratios at the discovery stage would substantially expand the search space (n(n–1)/2 combinations), increasing the risk of overfitting and poor generalizability. Instead, we focused on the covariance structure observed in the absence of disease and used reference markers to attenuate this structured non-disease variability, thereby improving signal-to-noise in detecting disease-related biomarker changes.

An alternative strategy would be to rely on data-driven models with embedded regularization, as applied in later analyses where the LAMP1:NPTX2 ratio was identified. However, this approach was not feasible in the main case-control analyses due to limited statistical power in some diagnostic groups.

The following changes have been made to the main text:

- Results: the relevant section has been updated with more direct interpretations, which we avoid pasting here to avoid clutter.
- Results/Methods: we have introduced the term cov_{nd} consistently
- Two panels of Figure 1 were updated for clarity
- Discussion: see R2:4

R3:7 Definition of cognitive impairment
The operational definition of cognitive impairment is unusual. Has it been validated?

Thank you for this remark. The definition of cognitive impairment was developed for this cohort to integrate performance across the full neuropsychological battery into a single metric and has not been externally validated. The definition follows a common statistical convention in which approximately 95% of observations in a normally distributed trait fall within ±2 SD of the mean, and performance beyond this range (i.e., below −2 SD relative to controls) may be considered indicative of abnormality. This operational definition was chosen to capture clear global impairment rather than subtle variation within the normal range.

As a sensitivity check, we examined performance across all individual cognitive tests (WAIS, D-KEFS, RCFT) in participants classified as cognitively normal or impaired. After scaling each test to the control mean for comparability, individuals defined as

cognitively impaired consistently performed worse across the full test battery (see Figure below). While this does not constitute formal external validation, it supports that the operational definition captures a subgroup with broadly reduced cognitive performance.

This figure and clarification have been added to the Supplementary Material.

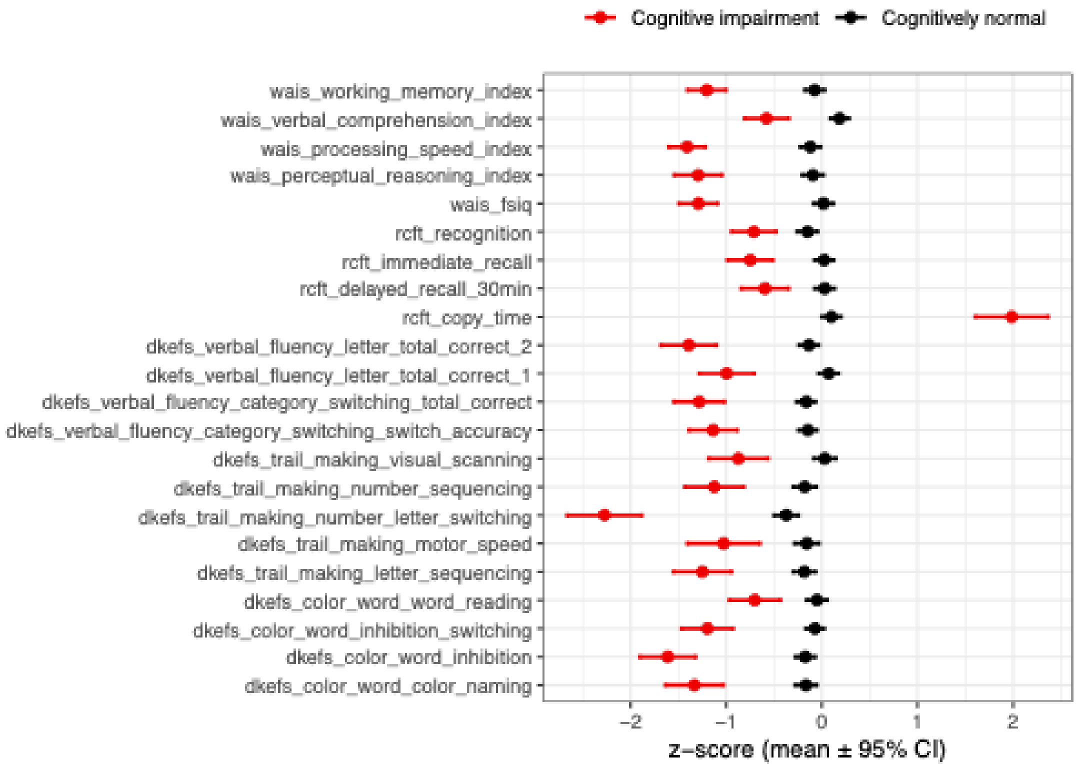

R3:8 PRS methodology
The reference or methodological details for polygenic risk score (PRS) computation are missing.

Thank you for identifying this omission. The reference describing the PRS methodology has now been added to the manuscript.

R3:9 Unadjusted vs. normalized CSF levels
The authors state they report unadjusted CSF levels. Are these raw concentrations without normalization? Were comparisons corrected for multiple testing? A figure analogous to Figure 1 should be provided for unadjusted values in the Supplementary Material. Additionally, a volcano plot may improve interpretability.

Thank you for this suggestion. By “unadjusted CSF levels,” we refer to raw CSF concentrations without normalization to cov_{nd}. We have now changed the terminology accordingly. Statistical analyses were corrected for multiple testing using the Benjamini–Hochberg false discovery rate, as described in the Methods.

To improve transparency, we have added the requested figure showing case–control associations based on raw CSF concentrations for synaptic proteins to the Supplement (Figure S1). Complete case–control results, both raw and cov_{nd}-adjusted, are provided in the Supplementary Tables. Volcano plots are addressed in R3:11.

R3:10 Incorrect figure reference
In line 182, the figure reference does not correspond to the text.

Thank you for finding this mistake, which has now been corrected.

R3:11 Clarity of case–control findings
The main findings from the case–control comparisons are difficult to interpret due to the plotting choices. It is unclear which proteins show cross-diagnostic vs. disorder-specific effects. For example, the manuscript states that NPTX2 and NPTXR mark SCZ+, yet later they are presented as pleiotropic markers. Both text and figures need to be revised to clearly present:
(i) which proteins differ across pair-wise comparisons,
(ii) which are disorder-specific, and
(iii) which are shared across diagnoses.
Consider alternative plotting formats (e.g., volcano plots, forest plots, Venn diagrams).

Thank you for this important comment. The distinction between shared versus relatively enriched effects is central to the interpretation of our results.

In this study, we aimed to balance two considerations. On the one hand, we wanted to display patterns and trends across disorders within a unified framework. On the other hand, we sought to avoid overinterpreting absence of statistical significance in smaller diagnostic groups, where power is limited. Because group sizes are imbalanced, binary overlap of statistically significant findings (e.g., Venn diagrams) can be misleading. A protein may appear “specific” to one group simply because that group has higher statistical power.

For this reason, we prioritized standardized effect sizes (β-values) displayed simultaneously across disorders, with formal significance (1% FDR) clearly marked. β-values were standardized to the same control group, enabling direct comparison across disorders. Using this approach, most synaptic protein alterations show consistent direction of effect across multiple groups. Markers such as NPTX2 and NPTXR exhibit the largest effect sizes in SCZ+, but similar directional changes are observed in other groups. We have revised the Results and Discussion to clarify that these findings reflect relative enrichment rather than disorder-specific alterations.

We considered alternative plotting approaches. Volcano plots illustrate within-disorder association strength but do not facilitate cross-disorder comparison of effect sizes. Forest plots allow cross-disorder comparison, but become visually dense with five case–control contrasts and >30 proteins, reducing interpretability. Venn diagrams emphasize overlap of statistically significant findings but risk overinterpreting non-significance in smaller groups as biological absence. Given these considerations, we retained the heatmap format and clarified interpretation in the text.

The following changes have been made to the main text (altered wordings in *italics*)

- Results (page 7): "*The strongest associations were more clearly differentiated in analyses adjusted for cov_{nd} (Figure 2A)*. In SCZ+, *large reductions were observed* for proteins involved in glutamate signaling, including the α-Amino-3-hydroxy-5-methyl-4-isoxazolepropionic acid (AMPA) receptor subunit GRIA4, neuronal pentraxins (NPTX2, NPTXR), and AP2B1. In ADHD, lower levels of lysosomal proteins (CTSD, LAMP1) and higher levels of SYT1 were observed (Figure 2D). BD1 and BD2+ shared lower levels of neurexin–neuroligin family proteins, involved in transsynaptic adhesion. *However, most associations were present in more than one disorder (e.g., CSTN1, NPTX2, NPTXR), with minor variation in effect size rather than categorical diagnostic specificity*. Lastly, certain *associations identified using raw CSF abundances (e.g., CNTN2, CPLX2) were attenuated after cov_{nd}, suggesting limited specificity beyond the shared covariance structure."*

R3:12 Value of markers significant only in unadjusted analyses
I do not agree that markers significant only in unadjusted analyses are necessarily of lesser interest. Independent validation in comparable external cohorts is needed.

We agree that results from unadjusted analyses are relevant, and therefore both raw and adjusted results are presented in the main text and Supplementary Tables. Our intention is not to dismiss findings based on raw CSF concentrations, but to evaluate whether accounting for non-disease covariance alters the strength and consistency of associations. The longitudinal control data show that a substantial proportion of variance reflects stable, individually determined covariance, which may obscure smaller disease-related effects. In our data, adjustment strengthened certain genetic associations and enabled detection of NPTX2 reductions in the external cohort.

Regarding a matched independent validation cohort, this is addressed in detail in our response to R3:19.

R3:13 Diagnostic performance and potential for multivariate models
Although some single-marker AUCs reach significance, the overall performance is limited, and top markers are shared across disorders. It would be worthwhile to evaluate multivariate models earlier in the analysis, as later done for symptom dimensions. However, a power analysis should first be performed. In this context, adjusted values may not be required, as multi-marker combinations can reduce intra-individual variability.

Thank you for these suggestions. As discussed in R1:1 and R3:11, the diagnostic groups in our cohort are uneven, with limited statistical power in some groups (notably AN, n=40). For this reason, we prioritized statistical approaches that could be applied consistently across all diagnostic comparisons. Resampling-based multivariate models would be unreliable in the smaller groups and prone to overfitting.

Regarding the diagnostic performance of single biomarkers, we agree that the observed AUC values do not indicate complete discrimination. However, given the biological

heterogeneity of psychiatric disorders, we do not expect single biomarkers to achieve full diagnostic separation. Instead, our aim was to identify biologically informative signals and patterns of association across disorders rather than optimize diagnostic classifiers for categorical diagnoses as defined according to the DSM. Please also refer to R1:2.

Multivariate models were applied in the latter part of the study to predict clinically meaningful dimensions—psychotic experience, cognitive impairment, and functional impairment—which have higher statistical power and may more closely reflect underlying neurobiological variation than diagnostic categories.

R3:14 Complement factors and NFL results
Complement and NFL findings should be plotted directly in the relevant figure. It is unclear why only the correlation between NPTX2 and C1q is shown, particularly since it is negative.

Thank you for this comment. Complement components and NfL results are included in Figure 2 and Table S3, and their case–control associations are reported alongside synaptic proteins. The specific correlation between NPTX2 and C1q was highlighted because these proteins have been experimentally linked, with NPTX2 shown to inhibit complement activity in the mammalian brain. The absence of a positive correlation in CSF therefore provides relevant context to hypotheses regarding complement-dependent synaptic pruning.

R3:15 Multivariate models for symptom associations
The multivariate associations with symptom domains show wide confidence intervals, suggesting substantial heterogeneity and/or limited sample size. Are these results reliable? Could controls and non-psychiatric cases be pooled? Were these models run with adjusted or unadjusted values? This point is important because a ratio of two proteins—likely better normalizing inter-individual variability—is used later. If ratios improve performance (as shown in Fig. 4 AUCs), they should be introduced earlier in the case–control analyses

Thank you for raising this concern. In response, we have refined the modeling framework to improve generalizability.

First, we clarify the original model specification. The models were trained as binary classifiers. For example, in the psychosis model, cases with psychotic experience were labeled as “cases,” whereas both healthy controls and cases without psychotic experience were grouped together as “controls.” When assessing model performance, we reported AUC values for both case–control and case–case comparisons to provide a transparent view of performance. As expected, separating cases from controls yielded higher AUC values, whereas case–case discrimination was more modest. The models were trained using raw CSF concentrations, as described in the main text. The rationale for not introducing biomarker ratios earlier in the discovery stage is discussed in R3:6 and remains unchanged.

Second, we updated the modeling approach to improve robustness. The original five-by-five-fold nested cross-validation was extended to a repeated nested cross-validation

framework. Specifically, the same structure was repeated ten times with different random seeds, resulting in 50 sets of held-out predictions (10 repeats × 5 outer folds). We now report mean (SD) AUC across outer folds, rather than the range from a single split.

Third, to avoid information leakage, recursive feature elimination was incorporated within the cross-validation framework. This yields 50 independent selections of biomarker pairs, allowing assessment of selection stability across folds.

For each contrast (psychotic experience, cognitive impairment, and functional impairment), we now report:

- mean (SD) AUC for case–control and case–case comparisons across outer folds
- summarized model coefficients from the L1-regularized models (Table S6)
- frequency of biomarker pair selection and corresponding coefficients (Table S7)

These changes are expected to result in more stable estimates across data splits. The main conclusions were unchanged. Synaptic biomarkers predicted the clinical contrasts, and the LAMP1:NPTX2 ratio performed similarly to the full multi-biomarker models. Across folds, LAMP1:NPTX2 was consistently selected for psychotic experience and was also the most frequently selected pair for functional impairment and cognitive impairment.

During revision, we also re-evaluated the clinical annotations by comparing interview data with register data. The definition of cognitive impairment and functional impairment was not changed, but the definition of psychotic experience was refined. In the updated definition, psychotic experience required consistent evidence across data sources (e.g., concordant information from interview and register data, or repeated register-based psychotic episodes). Cases with ambiguous or conflicting information (e.g., a single register event without support from interview data) were not classified as positive. This more specific definition was implemented to improve the reliability of the outcome, as requested by the reviewer. Table 1 was updated accordingly.

In this process, two SCZ+ cases did not meet the updated definition of psychotic experience. These cases had diagnoses F06.1 (ICD-10) / 293.89 (DSM-5) and F99 (ICD-10) / 300.9 (DSM-5). Both were recruited through the psychosis cohort and followed in specialized psychosis services. They were retained within the SCZ+ group but not classified as having psychotic experience in the modeling. Subdiagnoses in Table 1 were updated accordingly.

Lastly, we clarified the dataset used in these models. The models included all synaptic biomarkers (including synapse-expressed endolysosomal proteins), as well as NfL, CSF/serum albumin quotient, complement components related to synaptic remodeling (C1q, C3, C3b/iC3c, C4, C4b), and C5. This was not a change from the original submission but an inadvertent omission in the Methods description, which has now been corrected.

In summary, these updates improve the robustness of the analyses without changing the overall interpretation.

The following changes have been made to the main text:

- The Methods and Results sections were updated to reflect the revised modeling approach described above.
- Figure 3 was updated accordingly.
- A brief description of the clinical definitions of all transdiagnostic contrasts (not only cognitive impairment) has been added to the Methods. The passage now reads:

  "Psychotic experience was defined using combined interview and national register data, requiring either concordant evidence or multiple hospitalizations with discharge diagnoses of psychotic episodes. Cognitive impairment was assessed in a subset of participants who completed a comprehensive neuropsychological test battery, as described previously. Cognitive test data from the Wechsler Adult Intelligence Scale, the Delis-Kaplan Executive Function System, and the Rey Complex Figure Test were clustered, and data from controls were used to estimate the mean and standard deviation of cognitive performance. Cases scoring more than 1.96 standard deviations below the control mean on the first two principal components were classified as cognitively impaired. Extended details are available in the Supplementary Material. Functional impairment was defined as described above, requiring long-term sick leave in at least 80% of the observation period. Individuals receiving old-age pension were excluded from this analysis."

R3:16 Clarification of Figure 3d
The interpretation of Fig. 3d is not clear and needs clarification.

Thank you for identifying this mistake. The axis labels have now been corrected.

R3:17 Cognitive performance analyses
Please include direct references to the corresponding figures. The same issues of low power and high heterogeneity (visible in wide AUC ranges) are present in both the cognitive and functional impairment analyses. How were groups defined? Are the reported R^2 values correct?

Thank you for highlighting the need for direct figure references, which have now been added in the revised manuscript. The definition of the cognitive groups is described in the Methods including Supplementary Methods.

The relatively low R^2 values likely reflect the limited fit of a linear model. Inspection of the scatter plots suggests that the association is primarily driven by individuals with marked cognitive impairment rather than a linear relationship across the full range of cognitive performance (see also R1:4). The reported R^2 values are therefore correct and reflect heterogeneity in cognitive performance in this clinical population.

Because both protein concentrations and cognitive scores are influenced by age and sex, the reported R^2 values represent the incremental variance explained by the biomarker ratio beyond these covariates. To improve interpretability, we refined the analysis by estimating age and sex effects in the control group only and residualizing both the biomarker ratio and cognitive scores accordingly. This avoids potential influence of disease-related effects when adjusting for covariates. As expected, this led to only marginal changes in the reported R^2 values (e.g., general cognitive ability from 2.9% to 3.1% and executive function from 6.2% to 7.0%) and did not alter the overall interpretation. Nevertheless, we consider this approach more appropriate and have retained the updated values in the revised manuscript.

The following changes were made to the main text (altered wordings in *italics*):

- The statistics in the main text and corresponding figure have been updated accordingly.
- Methods (page 21): "*Variance explained in cognitive scores was estimated using linear regression models after residualizing both biomarker and cognitive variables for age and sex based on the control group*."

R3:18 Figure 4 AUC values
Figure 4 should include the explicit AUC values. Are the AUC improvements after adding PRS statistically significant?

We agree that including explicit AUC values in the figure improves interpretability, and these values have now been added.

We also appreciate the suggestion to formally test whether the improvement in model performance after adding PGS is statistically significant and we have compared ROC curves using DeLong's test.

During preparation of these analyses, we identified a minor data processing error related to how genetic and CSF data were combined. This affected the effective sample sizes (SCZ+: n=44→47; BD1: n=87→84), partly due to misclassification of cases with schizoaffective disorder. Reanalysis led to small changes in AUC values but did not alter the overall interpretation. Updated AUCs for LAMP1:NPTX2 versus LAMP1:NPTX2 combined with PGS were 0.80 and 0.84 for SCZ+, 0.75 and 0.81 for BD1, and 0.61 and 0.66 for BD2+, respectively. We apologize for this error, which has now been corrected, and the figure has been updated accordingly.

We compared ROC curves using DeLong's test. While the combined model showed numerically higher AUCs, the improvement did not reach conventional statistical significance in SCZ+ ($p = 0.07$), BD1 ($p = 0.07$), or BD2+ ($p = 0.11$). However, this absence of statistical significance should be interpreted with caution given the limited sample size, and we still consider the results to support future development of combined fluid and genetic biomarkers.

We refrained from directly comparing AUCs for the biomarker vs. the PGS, as such comparisons would be biased in favor of the biomarker, which was selected in these data.

The following changes were made to the main text (altered wordings in *italics*):

- Results (page 13): “Genetic data were available for 230 cases (*47* SCZ, *84* BD1, 97 BD2+) and 166 controls”
- Results (page 13): “However, combining LAMP1:NPTX2 with PGS-BD or PGS-SCZ *modestly improved* case–control discrimination, *although AUC improvements were not statistically significant (P=0.07–0.11)*: AUCs increased from *0.80 to 0.84* for SCZ, from *0.75 to 0.81* for BD1, and from *0.61 to 0.66* for BD2+ (Figure 4A). These findings *support further development* of combined genetic and fluid biomarkers for diagnostic stratification in psychiatric disorders.”
- Methods (page 21): “*ROC curves were derived from predicted probabilities, and AUCs were compared using DeLong’s test.*”

R3:19 Replication cohort and validation
How comparable are the FEP cases to the validation cohort? This seems more like an additional analysis rather than a true validation. A proper validation in an independent cohort with similar clinical characteristics is needed. Additionally, it is unclear which specific outcome the authors intended to validate. Please verify the last panel in Figure 4.

Thank you for this question. The external FEP cohort is comparable to the main cohort in that both were recruited from clinical psychiatric services using similar strategies, and both include cases and controls assessed within a structured research framework. The FEP cohort specifically targeted individuals at illness onset, resulting in a younger population (mean age=27, 46% female), although there was no significant case–control difference in age or sex within that cohort. We have added this demographic information to the main text.

We agree that this is not a formal replication cohort, as the clinical profiles differ and only a subset of biomarkers was available (NPTX2, SYUB). Accordingly, we have revised the manuscript to describe this as an extension rather than a replication (see response to R3:3). We do not claim replication of all findings, but we extend the observation that reduced NPTX2—particularly when adjusted for non-disease covariance—is detectable in psychotic disorders at illness onset. Similar associations between NPTX2 and recent-onset psychosis have also been reported in independent samples (Xiao et al., Sci Adv 2021).

Access to biobanked CSF material in psychiatric populations is limited, which constrains availability of fully matched replication cohorts. Nevertheless, the inclusion of an independent cohort with new cases and controls strengthens the robustness of the main finding.

The following changes have been made to the main text (altered wordings in *italics*):

- Results (page 13): "…, we validated synaptic biomarkers in an independent cohort of individuals with first-episode psychosis (FEP; n=60 *(25 female); mean age=28)* and controls (n=45 *(23 female); mean age=26)*."

Reviewer #1 (Remarks to the Author)

Authors have addressed the major comments by toning down the claims. However, some minor issues remain:
• Fig. 1F is called before Figs 1C-E.
• Line 227: "However, NfL concentrations correlated negatively with NPTX2 and NPTXR regressed on covnd (R = -0.32 and -0.40, respectively; Figure 2C). Fig. 2C only shows NPTX2.
• Fig 2D needs statistical analysis.

Thank you again for the comprehensive review. We have reordered the panels in Figure 1 so that they are cited sequentially, and added symbols indicating statistical significance thresholds to Figure 2D.

Regarding Figure 2C, we chose to show NPTX2 because it is a major focus of the manuscript. The corresponding association for NPTXR was similar (see figure below) and is therefore not shown. For clarity, we have revised the text to specify that Figure 2C displays the NPTX2 association.

The following change was made to the main text, with altered wording in italics:

- Results (page 8): "However, NfL concentrations correlated negatively with NPTX2 and NPTXR regressed on covnd (R = -0.32 and -0.40, respectively; Figure 2C shows NPTX2)."

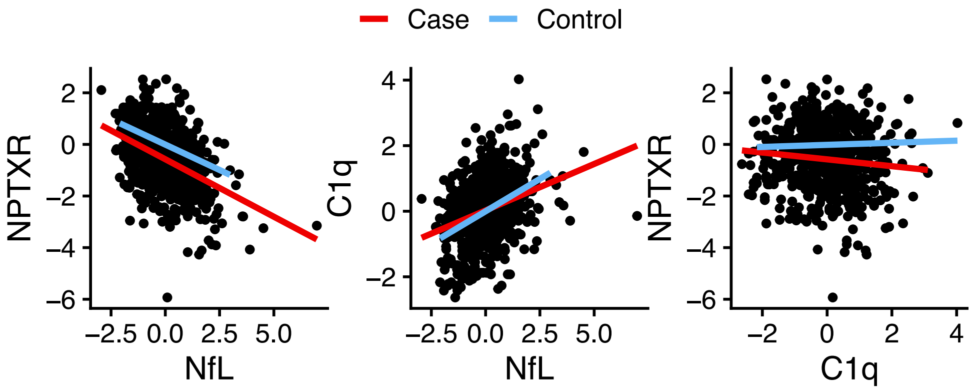

Figure 1. Pairwise correlation plots showing NfL, NPTXR, and C1q. Regression lines are shown separately for cases (red) and controls (blue). This figure is identical to Figure 2C, but with NPTX2 replaced by NPTXR.

Reviewer #2 (Remarks to the Author)

The authors effectively addressed my limited questions.

Thank you

Reviewer #3 (Remarks to the Author)

The authors have answer most of my concerns. There are still some limitations inherent to the limited sample size and the difficulty of obtaining such cohorts. But considerijg the lack of such type of studies in the pychiatry field, and the importance of it, I think the manuscript can be accepted for publication.

Thank you
